# Resin Glycosides from Convolvulaceae Family: An Update

**DOI:** 10.3390/molecules27238161

**Published:** 2022-11-23

**Authors:** Rani Maharani, Mohamad Fajar, Unang Supratman

**Affiliations:** 1 Department of Chemistry, Faculty of Mathematics and Natural Sciences, Universitas Padjadjaran, Jatinangor, Sumedang 45363, Indonesia; 2 Central Laboratory, Universitas Padjadjaran, Jatinangor, Sumedang 45363, Indonesia; 3 Centre of Natural Products Chemistry and Synthesis Studies, Faculty of Mathematics and Natural Sciences, Universitas Padjadjaran, Jatinangor, Sumedang 45363, Indonesia

**Keywords:** resin glycosides, convolvulaceae, oligoglycosidic acid, oligosaccharides, organic acids

## Abstract

Resin glycoside is a type of secondary metabolite isolated commonly from the Convolvulaceae family. It consists of oligosaccharides conjugated to organic acids with a larger percentage having a macrocyclic structure. The resin glycosides reported in this review is classified mostly based on the number of sugar units constructing the structure, which is correlated to the biological properties of the compounds. According to preliminary reviews, the protocols to isolate the compounds are not straightforward and require a special technique. Additionally, the structural determination of the isolated compounds needs to minimize the structure for the elucidation to become easier. Even though resin glycosides have a complicated structural skeleton, several total syntheses of the compounds have been reported in articles published from 2010 to date. This review is an update on the prior studies of the resin glycosides reported in 2010 and 2017. The review includes the classification, isolation techniques, structural determination, biological properties, and total synthesis of the resin glycosides.

## 1. Introduction

Resin glycosides are a class of natural products comprising oligosaccharides and organic acids, which are mostly obtained from the plants of the Convolvulaceae family [1,2]. The structures of these compounds are intramolecular macrocyclic esters formed through an intramolecular cyclization of the oligoglycosidic acid containing hydroxy fatty acids. However, several resin glycosides have acyclic aglycone [3,4,5] instead of forming macrocyclic structures, which are mostly acylated at the sugar moiety with varied organic acids [6,7,8,9,10]. The classification of this compound is based on the various number of sugar units inherent in the structure, which also form an ester-type dimer that makes the categorization process even larger. The unique structure of resin glycosides is also related to the biological properties of the compounds. Resin glycosides have broad biological properties, such as cytotoxic activities, MDR modulating properties, α-glucosidase inhibitory potential, antimicrobial activity, anti-metastatic activity, antiproliferative activity, neuroprotective and anticonvulsant activities [10,11,12,13,14,15,16]. Others include a sedative effect and GABA release, anti-inflammatory, antidepressant effect, anti-diarrhea, anti-bacteria, antimalaria, and downregulation of aquaporin 3 [15,17,18,19,20,21,22]. The isolation and characterization of this compound are somehow not straightforward. Therefore, special protocols are required to make successful isolation [23,24]. Mostly, the simplification of the resin glycoside structure through hydrolysis is required prior to the structural elucidation. Even though the structure of resin glycoside itself seems complicated, several total syntheses with numerous synthetic strategies have been reported and described in the present review.

A review of resin glycosides from the Convolvulaceae family in this review is an update to the prior review published in 2010 and 2017 [1,2]. The review in 2010 described many aspects of resin glycosides from the Convolvulaceae, including ethnobotanical aspects, structural diversity, isolation techniques, strategies for synthesis, and biological significance. The discussion in the 2017 review is not as large as the review in 2010. The review discussed two sections only, which are isolation and structural elucidation, and limited the discussion to four species of plants only. Recently, in 2022, a similar review regarding the update of resin glycosides from Convolvulaceae was reported [25]. The review discussed two sections, which are the structural diversity and biological properties of the resin glycosides. The present review described a comprehensive explanation of the classification, isolation, biological properties, structural determination, and total synthesis of resin glycosides of Convolvulaceae obtained from articles published from 2010 until 2022.

## 2. Classification

This section classified resin glycosides into eight groups, namely, monosaccharides, trisaccharides, tetrasaccharides, pentasaccharides, hexasaccharides, heptasaccharides, bidesmoside, and an ester-type dimer. The classification was mainly based on the number of sugars present in the structure, except for the last two-groups. The resin glycosides in the heptasaccharides and bidesmoside group were not reported in the 2010 review. Table 1 is a list of the classified resin glycosides.

### 2.1. Monosaccharides

**Figure 1:**
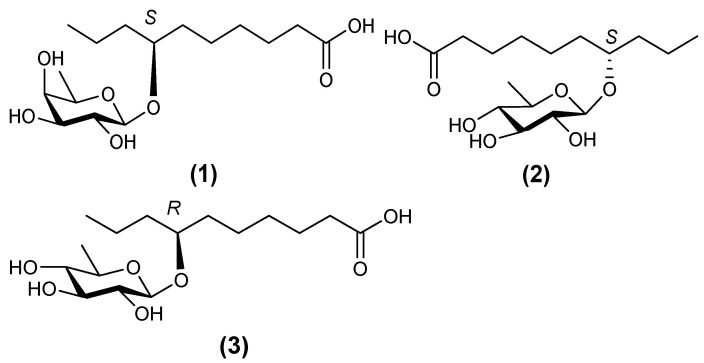
Structures of monosaccharide-based resin glycosides **1**–**3**.

The monosaccharide group, which consists of an aglycone as a decanoic acid (C-10) and one sugar group of either quinovose or fucose attached to C-7 of the decanoic acid, has the simplest structure. Fucose sugar is found in the compound of maltifidinic acid E (**1**) (Figure 1) isolated from the *Quamoclit × multifida* plant [26]. Meanwhile, the quinovose sugar is located in the compound of quamoclinic acid B (**2**) and operculinic acid K (**3**) (Figure 1) from the *Quamoclit pennata* [27] and *Operculina macrocarpa* plants [28]. Both quamoclinic acid B (**2**) and operculinic acid K (**3**) are diastereoisomers with methine carbon at C-7. The difference was found by measuring their optical rotation, where the compound (**2**) and compound (**3**) were [α${]}_{D}^{22}$−20.0 and [α${]}_{D}^{22}$−39.4, respectively. Furthermore, the acid hydrolysis of compound (**3**) produces a levo aglycone (a levorotatory aglycone) with an optical rotation of [α${]}_{D}^{22}$−3.3, supporting the presence of the *R* configuration at C-7. Based on these data, compound (**3**) was characterized as 7*R*-hydroxydecanoic acid 7-*O*-β-D-quinovopyranoside. This compound is rarely found as an aglycone or acyl side chain in the glycosidic resins of Convolvulaceae [28].

### 2.2. Trisaccharides

**Figure 2:**
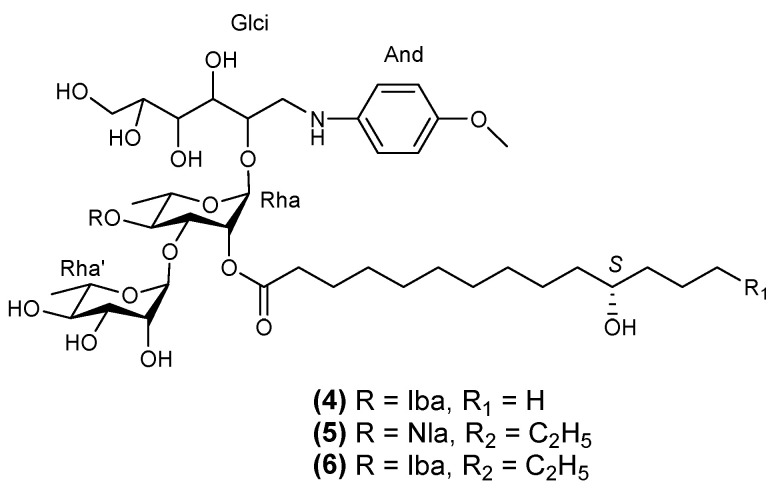
Structures of trisaccharide-based resin glycosides **4**–**6**.

Cuses-5–7 (**4**–**6**) (Figure 2) were trisaccharide-type resin glycosides isolated from the *Cuscuta chinensis* plant, where the three compounds are the result of a reductive amination reaction of a trisaccharide glycoside resin with *p*-anisidine [4]. The reaction was carried out because the glucose in the first sugar unit group on the anomeric carbon was not protected, easily underwent tautomerization, and was difficult to purify. For phytochemical studies, the ethanol fraction containing the resin glycoside was reduced with *p*-anisidine in order to protect the reducing sugar, thereby leading to an aminoalditol derivative grouped into disaccharide-type resin glycoside.

**Figure 3:**
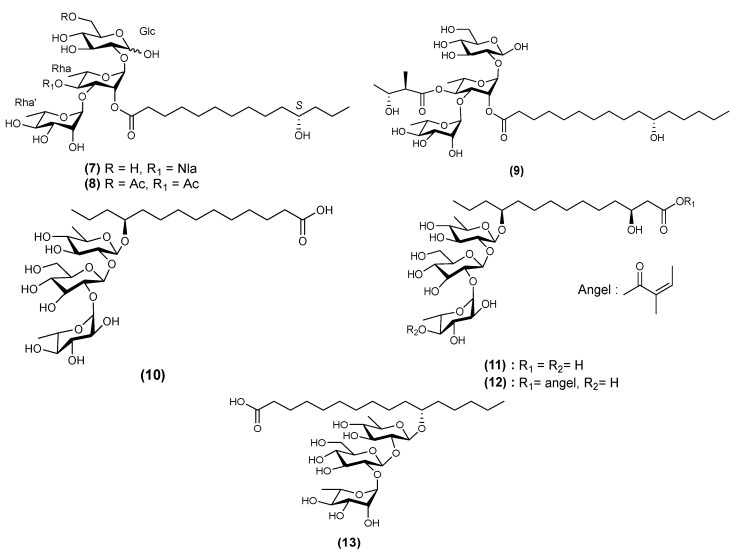
Structures of trisaccharide-based resin glycosides **7**–**13**.

Cuses-3 (**7**) and cuses-4 (**8**) (Figure 3) are trisaccharide-type resin glycosides isolated from *Cuscuta chinensis* [4]. The glucose, as the first sugar unit, was exposed and unprotected, which led to tautomerism, resulting in α and β-anomers. Similarly, dichondrin C (**9**) (Figure 3), a trisaccharide-based resin glycoside isolated from the *Dichondra repens*, was unprotected glucose in the first sugar unit. In its structure, the aglycone was attached to the second sugar unit, rhamnoside [3]. Poranic acid A-B (**10**–**11**) and A (**12**), isolated from *Porana duclouxii* [5], and stansoic acid A (**13**) (Figure 3), obtained from *Ipomoea stans* [16], showed a different structure. The trisaccharide has an aglycone attached to the first sugar unit group, hence, there is no unprotected sugar unit in the structure, and it resists tautomerism. The last four compounds have a sugar skeleton of 11-*O*-α-Rha-(1 → 2)-*O*-β-Glc-(1 → 2)-*O*-β-Qui-(1 → 11)-aglycone.

### 2.3. Tetrasaccharides

**Figure 4:**
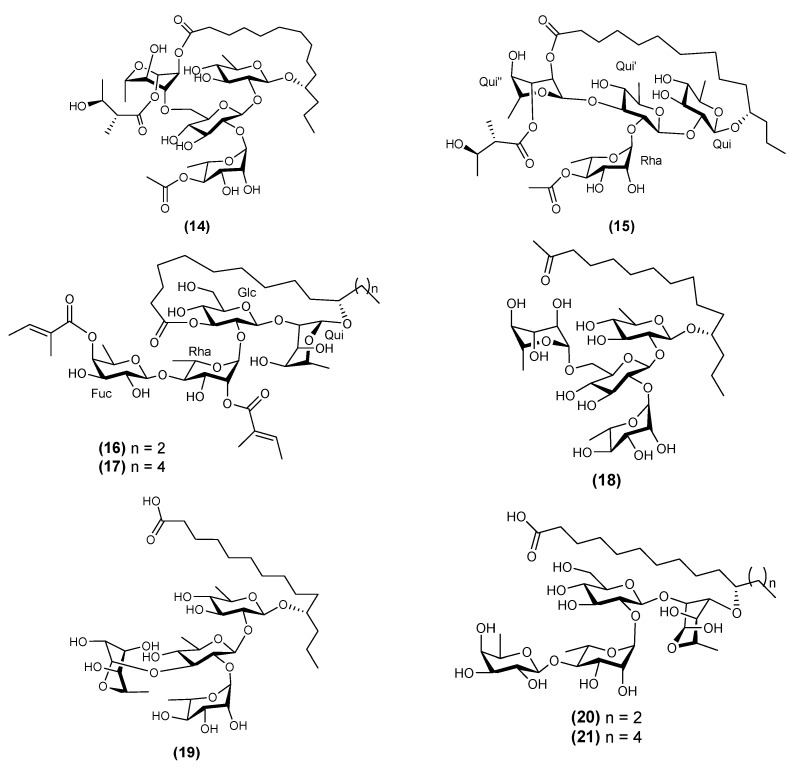

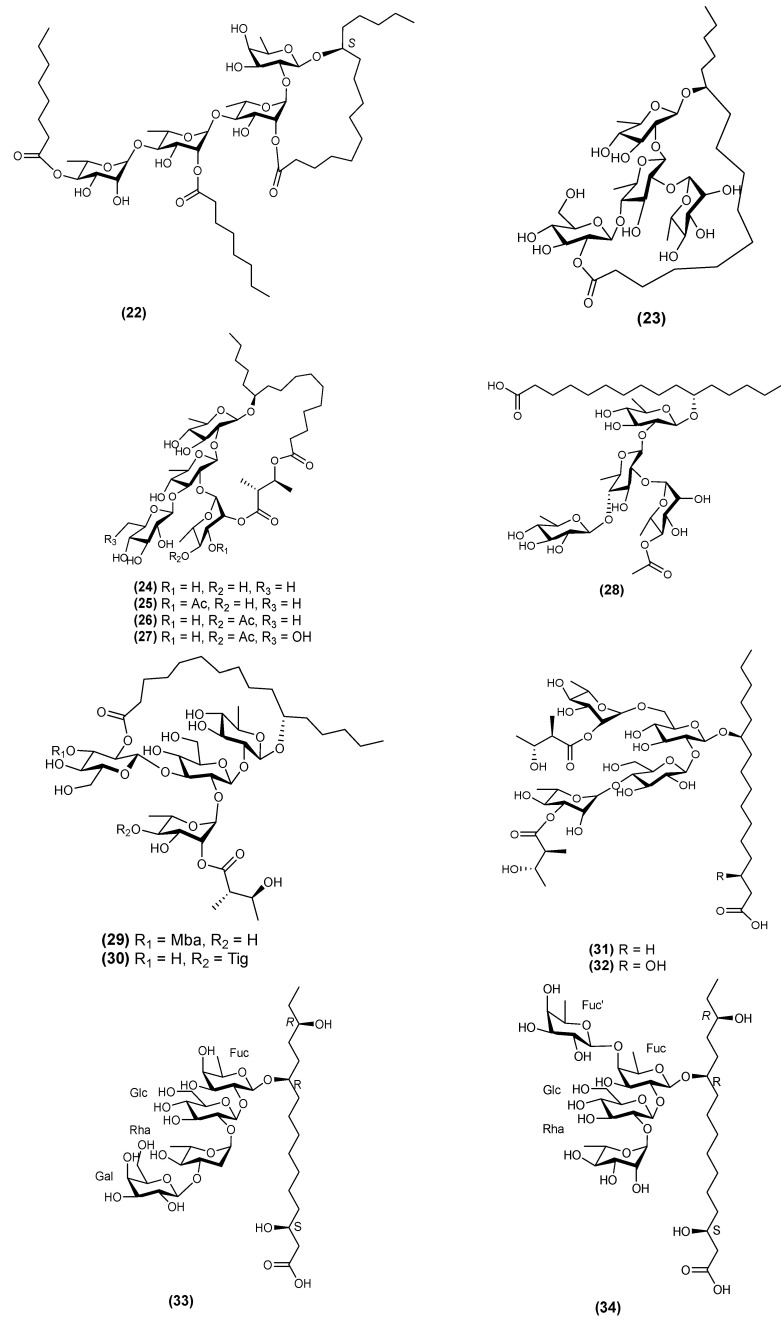

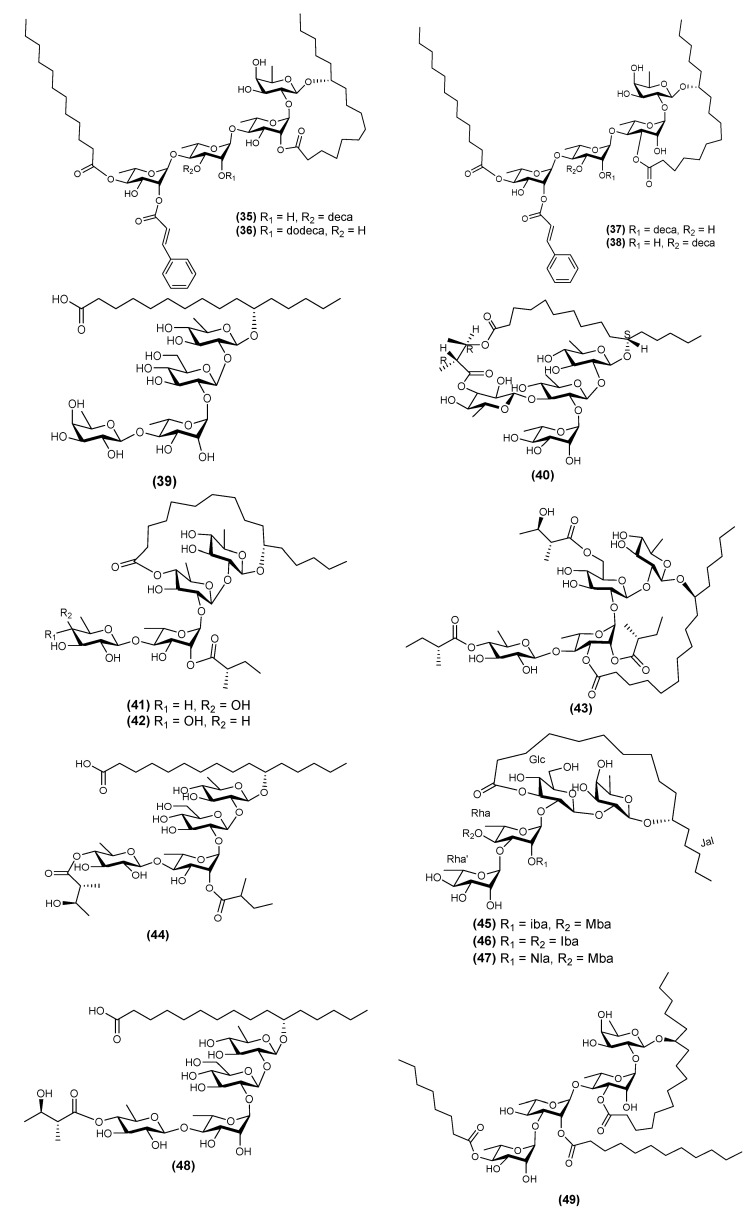
Structures of tetrasaccharide-based resin glycosides **14**–**49**.

Four macrocyclic aglycones of resin glycoside, albinoside VI–IX (**14**–**17**), and four of their glycosidic acids, albinosinic acid D g (**18**–**21**) were isolated from *Ipomoea alba* (Figure 4) [7]. All were categorized as tetrasaccharide-type resin glycosides, which are unique with varying structural skeletons. These include 11*S*-hydroxytetradecanoic acid 11-*O*-α-L-Rha-(1 → 2)-*O*-[β-D-Qui-(1 → 6)]-*O*-β-D-Glc-(1 → 2)-*O*-β-D-Qui of compound (**14**) with its glycosidic acid (**18**), then 11*S*-hydroxytetradecanoic acid-11*S*-hydroxytetradecanoic acid 11-*O*-α-L-Rha-(1 → 2)-*O*-[β-D-Qui-(1 → 3)]-*O*-β-D-Qui-(1 → 2)-*O*-β-D-Qui of compound (**15**) with its glycosidic acid (**19**), 11*S*-hydroxytetradecanoic acid 11-*O*-β-D-Fuc-(1 → 4)-*O*-α-L-Rha-(1 → 2)-*O*-β-D-Glc-(1 → 2)-*O*-β-D-Qui of compound (**16**) with its glycosidic acid (**20**), and lastly, 11*S*-hydroxyhexadecanoic acid 11-O-β-D-Fuc-(1 → 4)-*O*-α-L-Rha-(1 → 2)-*O*-β-D-Glc-(1 → 2)-*O*-β-D-Qui of compound (**17**) with its glycosidic acid (**21**).

Aquaterin XI (**22**) (Figure 4)is a tetrasaccharide-type resin glycoside with a linear chain consisting of three rhamnosyl sugar units and one fucosyl sugar unit [29]. It also has a structural skeleton of 11*S*-hydroxyhexadecanoic acid 11-*O*-α-rhamnopyranosyl-(1 → 4)-*O*-α-rhamnopyranosyl-(1 → 4)-*O*-α-rhamnopyranosyl-(1 → 2)-*O*-β-fucopyranoside-(1,2″-lactone). This compound was successfully isolated from *Ipomoea aquatica*. 

The oligosaccharide core of calonyctin E (**23**) (Figure 4) was determined as rhamnosyl-(1 → 2)-[glucosyl-(1 → 3)]-quinosyl-(1 → 2)-quinosyl with 11*S*-jalapinolic acid (1,3″-lactone) as aglycone [23]. Calonyctin F-H (**24**–**26**) (Figure 4) almost have the same aglycone and oligosaccharide core, except the sugar unit D-glucose, which was changed to D-quinovose for the skeleton to become 11-*O*-α-L-rhamnosyl-(1 → 2)-*O*-[β-D-quinosyl-(1 → 3)]-*O*-β-D-quinosyl-(1 → 2)-*O*-β-D-quinovopyranoside. Calonyctin I (**27**) (Figure 4) has the same oligosaccharide core and aglycone as compound **23** and similarities with compounds **24**–**26** in which the aglycone is attached to a nilic acid (Nla) group. Calonyctin J (**28**) (Figure 4) is a glycosidic acid with the same oligosaccharide core and aglycone as compounds (**24**–**26**). Meanwhile, compounds (**23**–**28**) were isolated from *Ipomoea muricata*.

Calysolin I (**29**) and calysolin X (**30**) (Figure 4) are tetrasaccharide-based resin glycosides with an oligosaccharide core of 11-*O*-α-L-rhamnosyl-(1 → 2)-*O*-[β-D-glucosyl-(1 → 3)]-*O*-β-D-glucosyl-(1 → 2)-*O*-β-D-quinovopyranosyl and aglycone of 11*S*-jalapinolic acid (1,2″‴) [30,31]. Other series of calysolin compounds are widely distributed as various types of pentasaccharides, hexasaccharides, and heptasaccharides, isolated from *Calystegia soldanella*. 

Dichondrins A-B (**31**–**32**) (Figure 4), isolated from *Dichondrin repens*, have the same sugar sequence of 11-*O*-α-L-rhamnosyl-(1 → 4)-*O*-β-D-glucosyl-(1 → 2)-[*O*-α-L-rhamnosyl-(1 → 6)]-*O*-β-D-glucopyranoside with 11*S*-jalapinolic acid as an aglycone [3]. Dichondrin B (**32**) contains an additional OH group at C-3 of the aglycone (3,11-jalapinolic acid). Mosher method was used to determine the absolute configuration (Δδ = δ_S_ – δ_R_, Δδ_16*H*_ = −0.011, Δδ_2Ha_ = +0.02), and it revealed that the aglycone has an S configuration. Based on the data, the aglycone was determined as 3*S*,11*S*-jalapinolic acid.

The aglycones present in evolvulic acid B-C (**33**–**34**) (Figure 4) have similarities due to the presence of trihydroxy jalapinolic acid at positions C-3, C-11, and C-14 [32]. The absolute configuration of 3*S*,11*R*,14*R* was determined using the Mosher ester method. The aglycone was determined as 3*S*,11*R*,14*R*-trihydroxyhexadecanoic acid (3*S*,11*R*, and 14*R*-jalapinolic acid). Evolvulic acid B (**33**) has an oligosaccharide core of 11-*O*-β-D-galactosyl-(1 → 3)-*O*-α-L-rhamnosyl-(1 → 2)-*O*-β-D-glucosyl-(1 → 2)-*O*-β-D-fucopyranoside. Meanwhile, evolvulic acid C (**34**) has a very different oligosaccharide skeleton core, which is 11-*O*-α-L-rhamnosyl-(1 → 3)-*O*-β-D-glucosyl-(1 → 2)-[*O*-β-D-fucosyl-(1 → 2)]-*O*-β-D-fucopyranoside. Both compounds were isolated from *Evolvulus alsinoides*.

The resin glycosides (**35**–**38**) (Figure 4) isolated from aerial parts of *Ipomoea batatas* [18] have structures similar to the oligosaccharide core, which is 11-*O*-α-L-rhamnopyranosyl-(1 → 4)-*O*-α-L-rhamnopyranosyl-(1 → 4)-*O*-α-L-rhamnopyranosyl (1 → 4)-*O*-β-D-fucopyranoside. However, there are some differences, particularly in the position of the macrocyclic aglycone ester bonds. Ipomotaoside A-B (**35**–**36**) has a jalapinolic acid aglycone at the position of the 1,2″-ester. Meanwhile, ipomotaoside C-D (**37**–**38**) has the ester bond at 1,3″.

The structure of muricatin D (**39**) (Figure 4) isolated from *Ipomoea muricata* [33] was determined as 11*S*-jalapinolic acid 11-*O*-β-D-fucopyranosyl-(1 → 4)-*O*-α-L-rhamnopyranosyl-(1 → 2)-*O*-β-D-glucopyranosyl-(1 → 2)-β-D-quinovopyranoside. It was reported to be similar to compound (**21**) and inconsistent with ^1^H- and ^13^C-NMR chemical shifts of **39**. Muricatin IX–XI (**40**–**42**) (Figure 4) [34] was also isolated from the same plant species. All resin glycosides have the same oligosaccharide core as muricatin I–VI [72] with three sugar units, namely quinovose (qui′), quinovose (qui″) and rhamnose (rha‴). The fourth unit can be either quinovose or fucose (qui″‴ or fuc″‴) with the presence of an aglycone known as jalapinolic acid. In muricatin IX (**40**), the fourth sugar unit, quinovose (qui″‴), was attached to the second, quinovose (qui″). Similarly, the jalapinolic acid ester of **40** was attached to nilic acid (Nla) at C-3. However, this is in contrast to muricatin X–XI (**41**–**42**) in which the fourth sugar unit quinovose or fucose (qui″‴ or fuc″‴) is attached to rhamnose (rha‴) with the aglycone, which is attached to the qui″ at C-3 (ester-1.3″).

There are a least two tetrasaccharide-type resin glycosides obtained from *Ipomoea stans*, namely, stansin 6 (**43**) [10,35] and stansinic acid I (**44**) (Figure 4) [16]. Both compounds have a similar oligosaccharide core, 11-*O*-β-D-quinovopyranosyl-(1 → 4)-*O*-α-L-rhamnopyranosyl-(1 → 2)-*O*-β-D-glucopyranosyl-(1 → 2)-*O*-β-D-quinopyranoside, with a varying aglycone moiety. Stansin 6 (**43**) has a macrocylic aglycone (1,3‴-ester), while stansinic acid I (**44**) contain an acyclic aglycone.

The other series of tricolorins was also found from *Ipomoea tricolor*, which are tricolorin K-M (**45**–**47**) (Figure 4) [12]. These resin glycosides share the same oligosaccharide framework as tricolorins A-D [73], which is 11-*O*-α-L-rhamnosyl-(1 → 3)-*O*-α-L-rhamnosyl-(1 → 2)-*O*-β-D-glucosyl-(1 → 2)-*O*-β-D-fucopyranoside, with a macrocyclic aglycone that is bound to the second sugar unit (glu″) at C-3 (ester-1,3″). The structural difference is only found in the placement of nilic acid (Nla) and methyl butyric acid (Mba) at C-2 and C-3 in the third sugar unit (rha‴).

The tetrasaccharide-based resin glycoside, stansinic acid I (**44**), has a structure of 11-*O*-β-D-Qui-(1 → 4)-*O*-α-L-Rha-(1 → 2)-*O*-β-D-Glc-(1 → 2)-*O*-β-D-Qui with acyclic jalapinolic acid, which acts as the aglycone [17]. The structure is similar to that of tyrianthinic acid VI (**48**) (Figure 4). This compound was obtained from *Ipomoea tyrianthina*, where the difference was only in the absence of the methyl butyric acid (Mba) group at C-2 in the second sugar unit (Rha‴).

Wolcottinoside I (**49**) (Figure 4) isolated from *Ipomoea wolcottiana* [8] has a similar structure to ipomotaoside C-D compound (**37**–**38**) obtained from *Ipomoea batatas* [18]. The difference between both compounds is the absence of a dodecanoyl side group (Dodeca) at C-2 in the third sugar unit Rha‴ and an octanoyl group (Octa) at C-4 in the fourth sugar unit Rha.

### 2.4. Pentasaccharides

**Figure 5:**
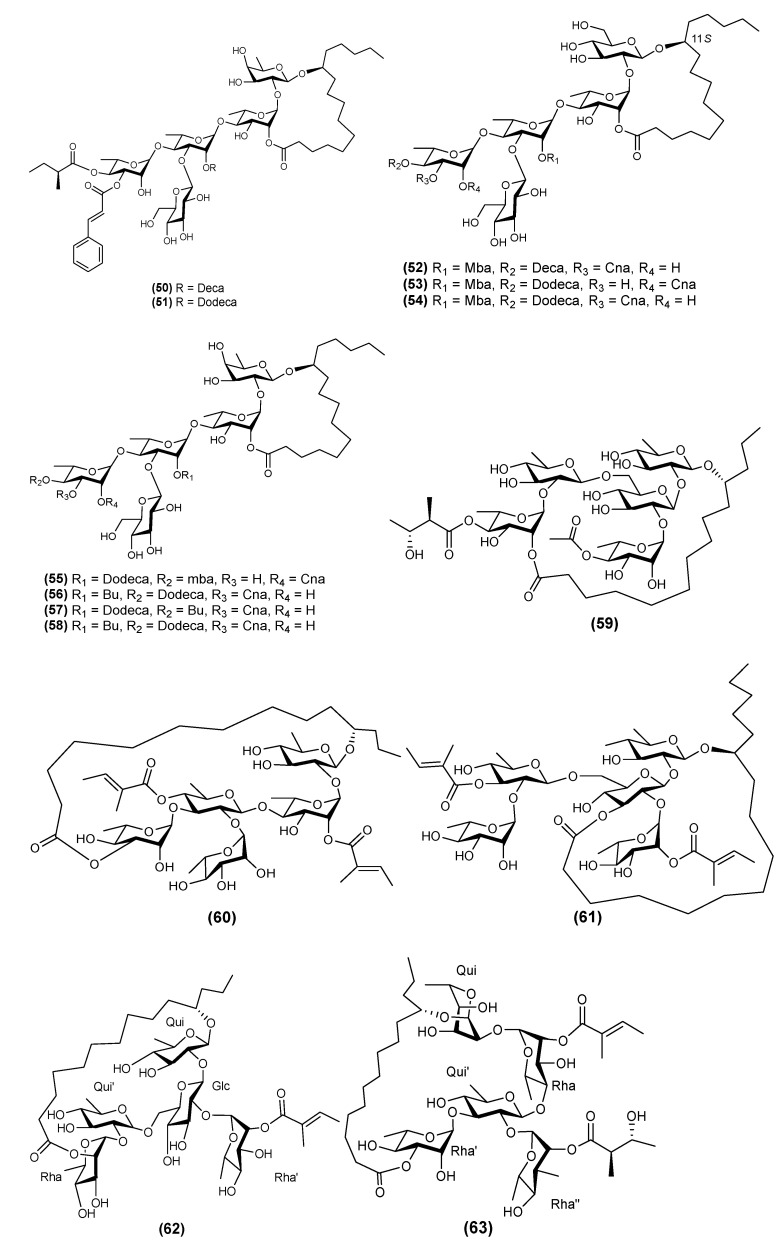

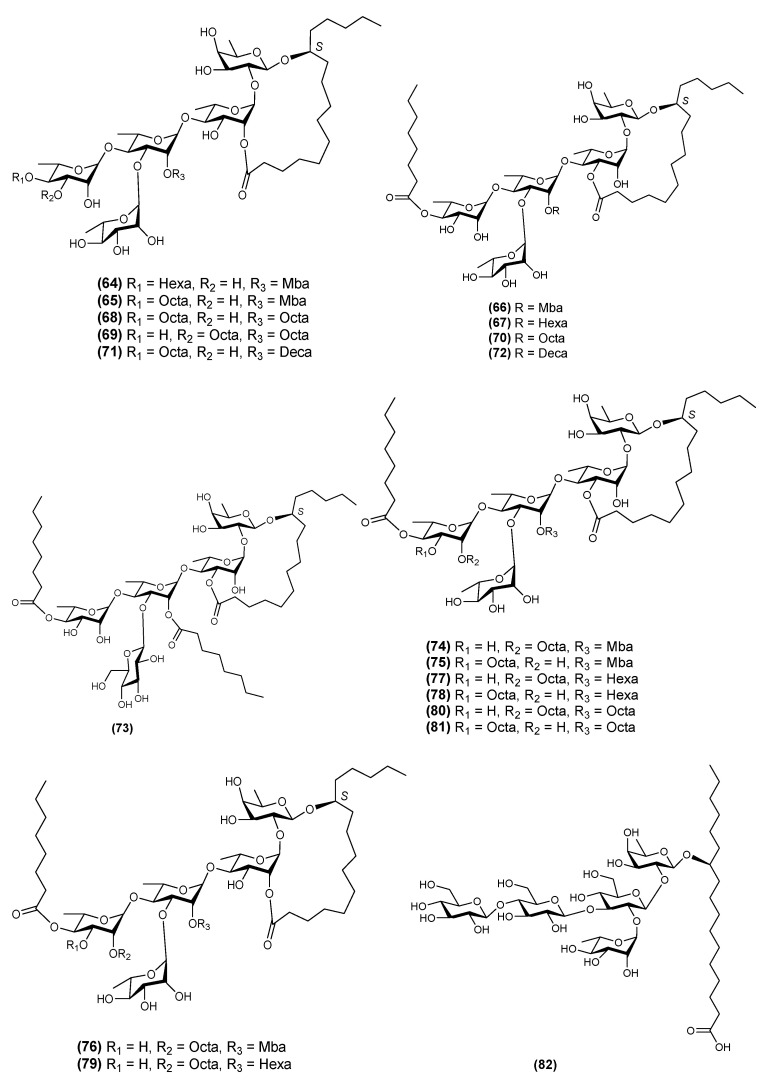

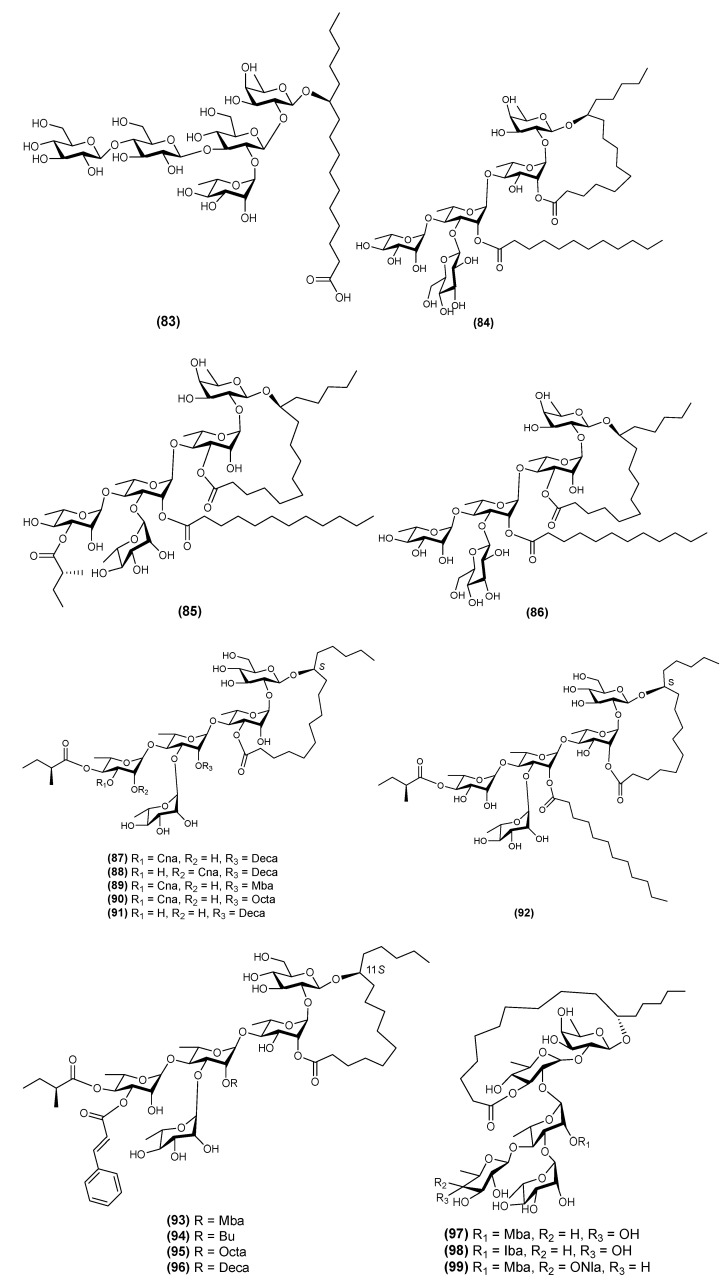

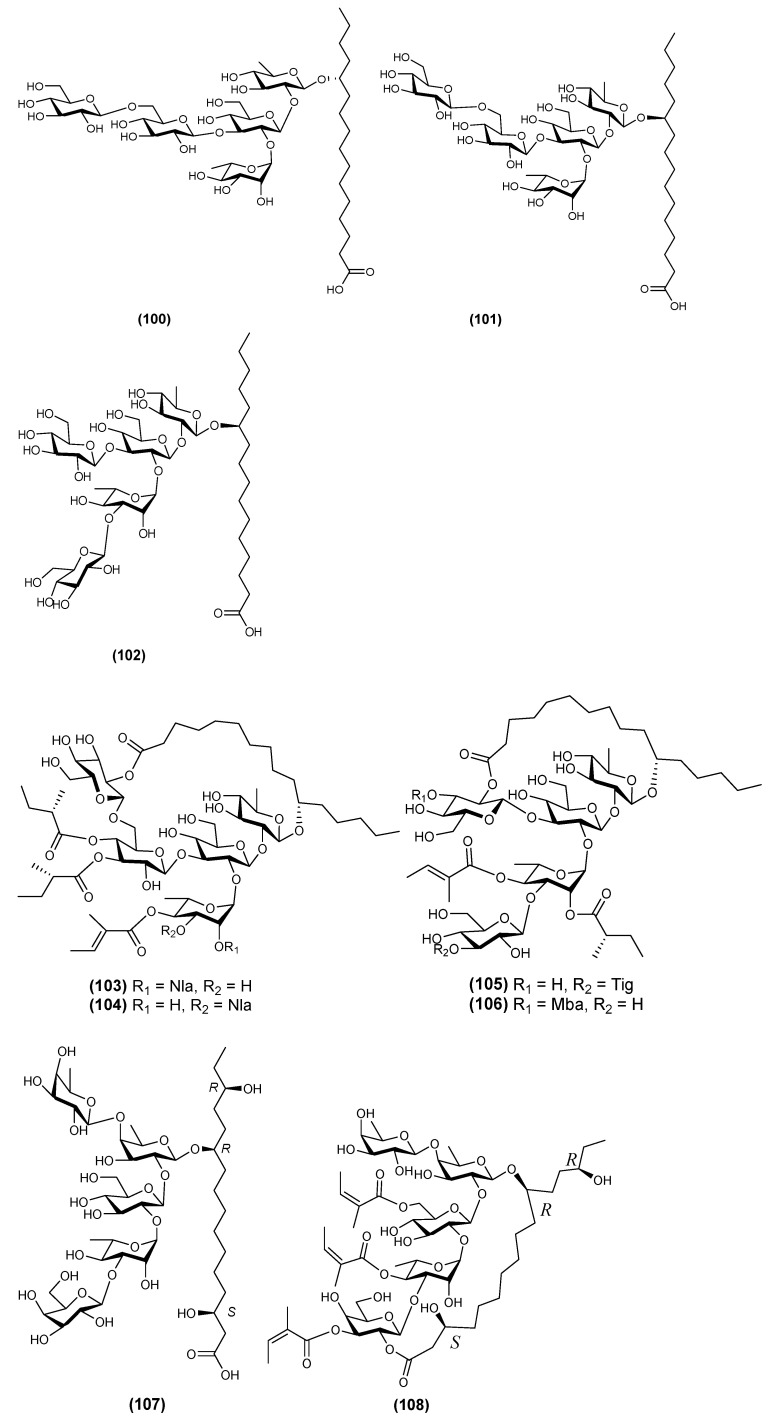

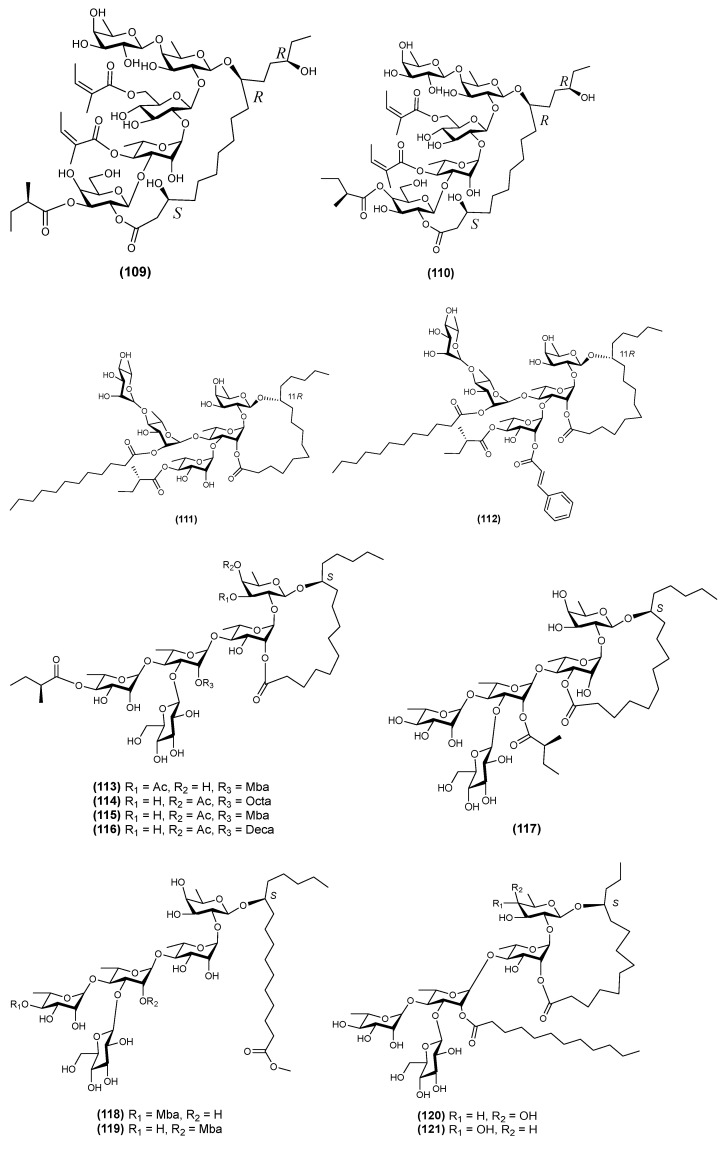

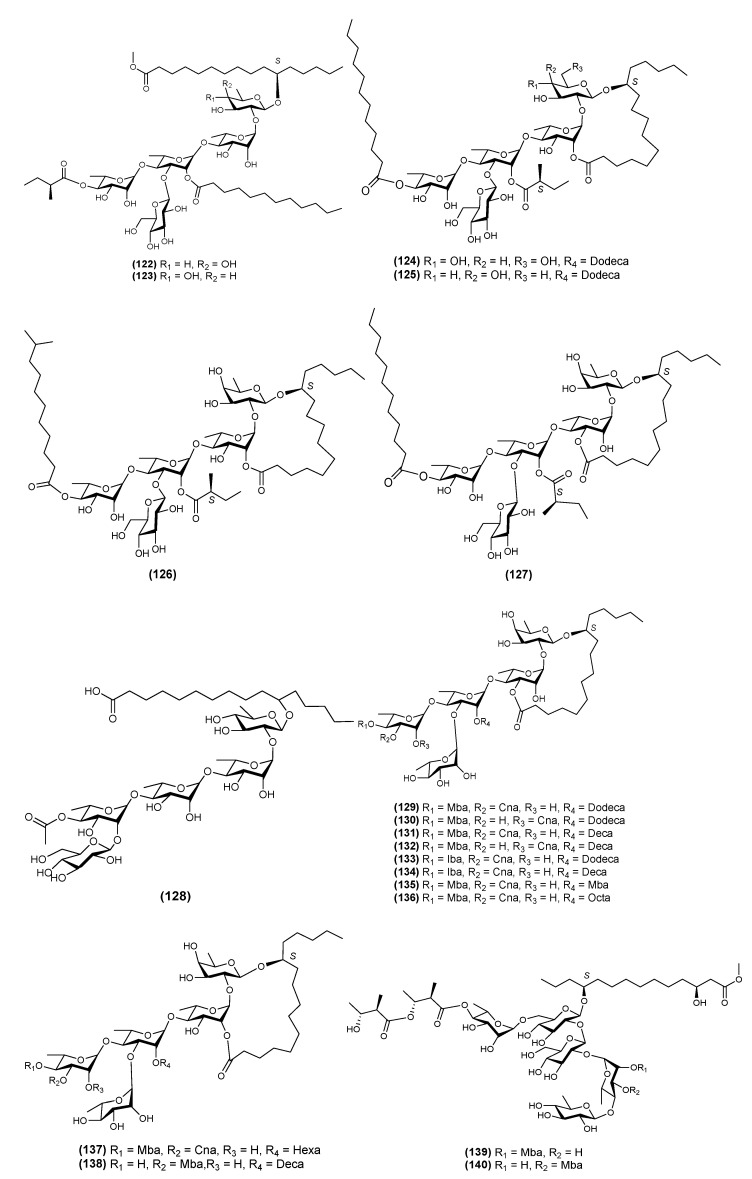

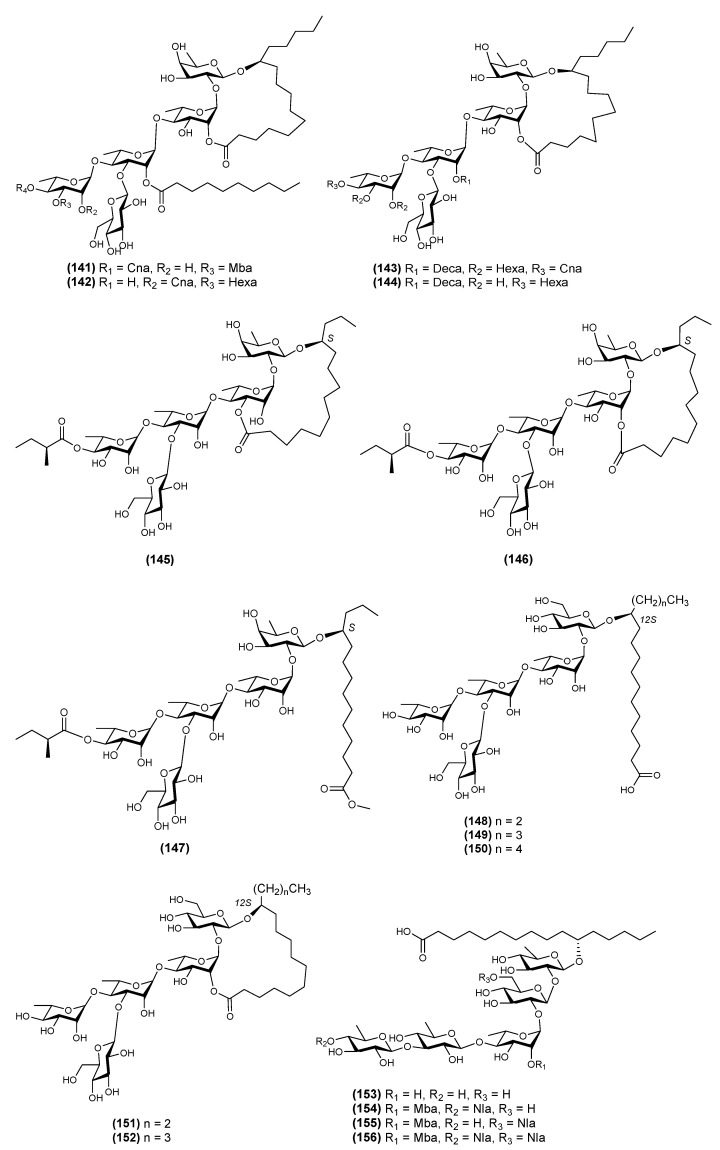

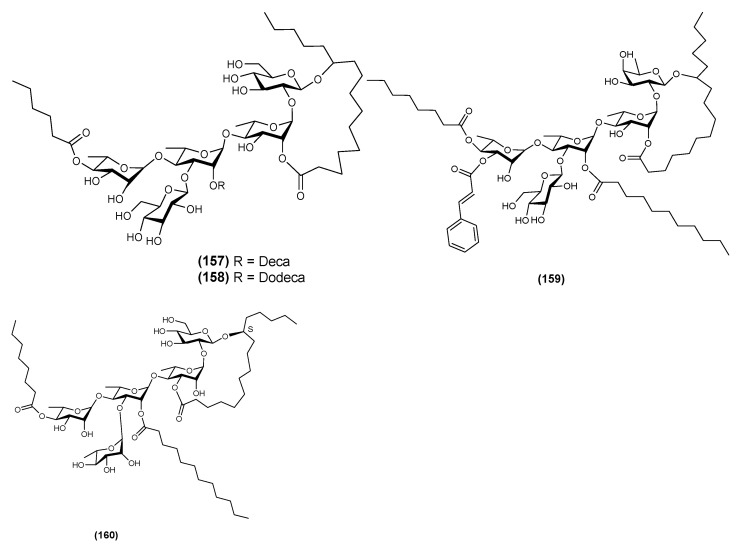
Structures of pentasaccharide-based resin glycosides **50**–**160**.

Acutacosides A-B (**50**–**51**) (Figure 5) are resin glycosides containing a pentasaccharide core in their structure [8]. These compounds were isolated from *Argyreia acuta* and have the same structural framework that is characterized by the presence of similar carbon signals in the ^13^C-NMR, particularly of the chemical shift from 177 to 41 ppm. The oligosaccharide core of these compounds is 11-*O*-α-L-rhamnopyranosyl-(1 → 3)-*O*-[α-L-rhamnopyranosyl-(1 → 4)]-*O*-α-L-rhamnopyranosyl-(1 → 4)-*O*-α-L-rhamnopyranosyl-(1 → 2)-*O*-β-fucopyranoside with an intramolecular aglycone 1,2″-ester. The only difference is that acutacoside A (**50**) and acutacoside B (**51**) have decanoic and dodecanoic groups, respectively. Acutacosides C-E (**52**–**54**) [36] were isolated, where the structures had the same framework as acutacosides A-B (**50**–**51**). However, the first sugar unit of acutacosides C-E (**52**–**54**) was glucose, while for acutacosides A-B (**50**–**51**) it was fucose. The difference between the structure of acutacosides C-E (**52**–**54**) was in the position of the cinnamoyl acid (Cna) and the dodecanoyl (Dodeca) groups at C-2, C-3, and C-4 of the fourth sugar unit (rha″‴). Acutacosides F-I (**55**–**58**) [37] have the same oligosaccharide core as acutacosides A-B (**50**–**51**); the difference is in the presence of organic acid groups, namely, methyl butyric acid (Mba), cinnamic acid (Cna), dodecanoyl (Dodeca), and butyl (Bu) at the third (Rha‴) and fourth (Rha″‴) sugar units.

In 2012, albinosides I–III (**59**–**61**) (Figure 5) were isolated from *Ipomoea alba*. Based on GC-MS analysis, these resin glycosides revealed the presence of sugar units of rhamnose (Rha), quinovose (Qui), and glucose (Glc) in a ratio of 2:2:1 [38]. HMBC analysis was used to determine the location of the aglycone in the structure. Based on the HMBC analysis, macrolactone was located at C-2, while the aglycone of albinoside I (**59**) was found in the terminal sugar unit of rhamnose (Rha), which was shown through a correlation of H-2 (5.60) with C-1 (173.5) of convolvulinic acid. The macrolatone of albinoside II (**60**) is located at C-3 on the terminal sugar unit rhamnose (Rha) with a correlation of H-3 (5.66), as well as C-1 (171.1) of convolvulinic acid. For albinoside III (**61**), the macrolactone is located at C-3 of the second sugar unit, and glucose (Glc) is indicated by a correlation between H-3 (5.78) and C-1 (173.0) jalapinolic acid. Based on its oligosaccharide core, albinoside IV (**62**) [7] has the same sugar framework as albinoside I (**59**). However, there is a tigloyl (Tig) group in albinoside IV (**62**) (Figure 5) that is attached to the third sugar unit, known as rhamnose (Rha′). Albinoside V (**63**) (Figure 5) has the same framework as the albinoside II (**60**). The difference between albinoside V (**63**) and albinoside II (**60**) with albinoside I (**59**) and albinoside III (**61**) is in the second sugar unit, whereby glucose is replaced with rhamnosyl sugar; hence, the oligosaccharide core becomes 11-*O*-α-L-rhamnosyl-(1 → 2)-*O*-β-D-quinosyl-(1 → 2)-*O*-[α-L rhamnosyl-(1 → 3)]-*O*-α-L-rhamnosyl-(1 → 4)-*O*-β-D quinosyl [7].

Resin glycoside from *Ipomoea aquatica* produced eleven aquaterins I–XI, consisting of ten pentasaccharide-type resin glycoside, aquaterins I–X (**64**–**73**) (Figure 5), and one tetrasaccharide group, aquaterin XI (**22**) [29]. Structurally, there are three types of pentasaccharide aquaterins compounds (**64**–**73**). The first is the oligosaccharide core rhamnosyl-(1 → 4)-*O*-[rhamnosyl-(1 → 3)]-*O*-rhamnosyl-(1 → 4)-*O*-rhamnosyl-(1 → 4)-*O*-fucopyranosyl, with a macrocyclic aglycone of jalapinolic acid attached to the C-2 rha sugar” (ester-1,2″) for the compound aquaterins I–II (**64**–**65**), V–VI (**68**–**69**), and VIII (**71**). The second contains a similar oligosaccharide core as the first, but the jalapinolic acid is attached to C-3, the rha sugar unit (ester-1,3), for aquaterins III–IV (**66**–**67**), VII (**70**), and IX (**72**). Finally, the third framework has a difference in the sugar units, where the fourth is replaced with a glucose sugar unit, thereby producing an oligosaccharide core of rhamnosyl-(1 → 4)-*O*-[glucosyl-(1 → 3)]-*O*-rhamnosyl-(1 → 4)-*O*-rhamnosyl-(1 → 4)-*O*-fucopyranosyl with jalapinolic acid aglycone (ester-1,3″) for aquaterin X (**73**). In the following year, eight resin glycosides were isolated, namely, aquaterins XII–XIX (**74**–**81**) (Figure 5) [39]. These compounds have the same oligosaccharide core structure as aquaterins I–IX compounds (**64**–**72**). The difference is only in the placement of the macrocyclic aglycones of jalapinolic acid. The ester-1,2″ type is for aquaterins XII–XIV (**74**–**76**), XVI (**78**), XVIII–XIX (**80**–**81**), while the ester-1,3″ type is for aquaterins XV (**77**) and XVII (**79**).

Arvensis K-L (**82**–**83**) (Figure 5) were found in the plants of *Convolvulus arvensis* [40]. Both compounds have the same oligosaccharide core, which is glucosyl-(1 → 4)-*O*-glucosyl-(1 → 4)-*O*-[rhamnosyl-(1 → 2)]-*O*-glucosyl-(1 → 4)-*O*-fucopyranosyl. The acyclic aglycone contains different structures, where arvensis L (**83**) has a common aglycone, and 11*S*-hydroxyhexadecanoic acid (or 11*S*-jalapinolic acid), while arvensis K (**82**) has a new aglycone with an additional one carbon, which is 11*S*-hydroxyheptadecanoic acid.

The plant of *Ipomoea batatas* produced batatinoside VII–IX (**84**–**86**) (Figure 5) [41], a resin glycoside with the same oligosaccharide framework as aquaterins [29]. Batatinoside VII (**84**) shares similar oligosaccharide cores with aquaterins I–II (**64**–**65**) and macrocyclic aglycones at C-2 rha units (ester-1,2″). Furthermore, batatinoside VIII (**85**) is similar to aquaterin X (**73**), in which one rhamnose sugar is replaced by glucose. Meanwhile, batatinoside IX (**86**) has the same characteristics as aquaterin III (**66**) with a similar ester-1,3″ type. Although they have the same oligosaccharide core, the difference between batatinosides VII–IX (**84**–**86**) and aquterins is in the presence of a dodecanoyl (dodeca) group at C-2 of the third sugar unit and a rhamnosyl sugar [41].

In 2013, six resin glycosides were isolated from *Ipomoea cairica*, namely, cairicosides A-F (**87**–**92**) (Figure 5) [42]. At first glance, the oligosaccharide core is somewhat similar to aquaterins [29] or batatinosides VII–IX (**84**–**86**)[41], but the first sugar unit of the cairicosides is glucose; hence, the structure becomes rhamnosyl-(1 → 4)-*O*-[rhamnosyl-(1 → 3)]-*O*-rhamnosyl-(1 → 4)-*O*-rhamnosyl-(1 → 2)-*O*-glucopyranosyl. Cairicoside A-E (**87**–**91**) has a macrocyclic aglycone attached to the C-3 of the second sugar unit, rha′ (ester-1,3″). Meanwhile, the aglycone of cairicoside F (**92**) is attached to its C-2 (ester-1,2″ type). The plant *I. cairica* also produced four resin glycosides, namely cairicosides I–IV (**93**–**96**) (Figure 5) [43]. These compounds have the same framework as cairicoide F (**92**), with a 1,2″ ester type. The difference among the four structures is only in the organic acid groups attached to the C-2 of the third sugar unit, which can be either butanoyl (Bu), methyl butyric acid (Mba), octanoyl (Octa), or decanoyl (Deca).

Calonyctins found in *Ipomoea muricata* are of the tetrasaccharide and pentasaccharide type [23]. The pentasaccharide-typed resin glycosides of calonyctins B-C (**97**–**98**) (Figure 5) have the structural framework as the oligosaccharide core of rhamnosyl-(1 → 3)-*O*-[quinosyl-(1 → 4)]-*O*-rhamnosyl-(1 → 2)-*O*-quinosyl-(1 → 2)-*O*-fucosyl. Meanwhile, calonyctin D (**99**) (Figure 5) is only different in its fourth sugar, in which quinovose is replaced with fucose. Calonyctins B-D (**97**–**99**) have a macrocyclic aglycone of jalapinolic acid and are attached to C-3 in the second sugar unit, qui″ (ester-1,3″ type). 

All plant parts of *Calystegia hederacea* were found to produce four glycosidic acids, namely, calyhedic acids A-D [44]. However, of these four resin glycosides, only calyhedic acid A (**100**) (Figure 5) belongs to the type of pentasaccharide group with a composition of three glucoses, one quinovose, and one rhamnose sugar unit, accompanied by an acyclic aglycone 12-hydroxyhexadecanoic acid. 

Calysolic acids A and B (**101**–**102**) (Figure 5) were isolated from the fresh leaves, stems, and roots of *Calystegia soldanella* [45]. Structurally, both acids differ in the order of the oligosaccharide core, where calysolic acid A (**101**) has a core of rhamnosyl-(1 → 2)-[*O*-glucosyl-(1 → 6)-*O*-glucosyl-(1 → 3)]-*O*-glucosyl-(1 → 2)-*O*-quinopyranosyl, while calysolic acid B (**102**) is glucosyl-(1 → 3)-*O*-rhamnosyl-(1 → 2)-*O*-[glucosyl-(1 → 3)]-*O*-glucosyl-(1 → 2)-*O*-quinopyranoic acid. Both compounds contain acyclic aglycone 11*S*-hydroxydecanoic acid. *C. soldanella* was also found to produce calysolins I–IX [30,46]. Calysolin II–III (**103**–**104**) (Figure 5) has an oligosaccharide core skeleton, which is α-L-rhamnopyranosyl-(1 → 2)-[*O*-β-D-glucopyranosyl-(1 → 6)-*O*-β-D-glucopyranosyl-(1 → 3)]-*O*-β-D-glucopyranosyl (1 → 2)-*O*-β-D-quinopyranoside, and jalapinolic acid aglycones (with ester-1,2″ type). Both compounds have differences in the location of the nilic acid (Nla) group, which is attached to the sugar rhamnose unit at its C-2 or C-3 [30]. Meanwhile, calysolin V–VI (**105**–**106**) (Figure 5) has a β-D-glucopyranosyl-(1 → 3)-*O*-α-L-rhamnopyranosyl (1 → 2)-[*O*-β-D-glucopyranosyl-(1 → 3)]-*O*-β-D-glucopyranosyl-(1 → 2)-*O*-β-quinopyranoside. The difference between the structures is in the calysolin V (**105**), which has a tiglic acid group (tig) on C-3 of glucose (glc″), as opposed to calysolin VI (**106**). Another difference is that there is a methyl butyric acid (Mba) group on the C-4 glucose unit (glc′) in calysolin VI (**106**), which was absent in calysolin V (**105**) [46].

Evolvulic acid A (**107**) and evolvulin I–III (**108**–**110**) (Figure 5) have the same oligosaccharide core as β-D-galactopyranosyl-(1 → 3)-*O*-α-L-rhamnopyranosyl (1 → 2)-*O*-β-D-glucopyranosyl-(1 → 2)-[*O*-β-D-fucopyranosyl-(1 → 4)]-*O*-β-D-fucopyranoside [47,48]. These compounds are found in *Evolvulus alsinoides* plants. The most interesting fact about these compounds is the presence of a novel aglycone, which is a 3,11,14-trihydroxyhexadecanoid acid unit, a new fatty acid derivative obtained from nature. The absolute configuration of the aglycone was determined by the Mosher method using an MTPA reagent. Meanwhile, the aglycone was derived as 3*S*,11*R*, and 14*R*-trihydroxyhexadecanoid acid [47].

The two new glycoside resins obtained by the plant *Ipomoea pes-caprae*, ipomeolides A and B (**111**–**112**) (Figure 5), had an unusual structure with the aglycone of 11*R*-jalapinolic acid [49]. The configuration of macrolactones with 11*R* has not been reported, where the majority of resin glycosides have an 11S configuration. Both compounds have an oligosaccharide core rhamnosyl-(1 → 4)-*O*-rhamnosyl-(1 → 4)-[*O*-rhamnosyl-(1 → 3)]-*O*-rhamnosyl-(1 → 2)-*O*-fucopyranosyl-(1,2″-lactone).

Merremins A-G (**113**–**119**) (Figure 5), isolated from *Merremia hederacae,* have an oligosaccharide core of glucosyl-(1 → 3)-*O*-[rhamnosyl-(1 → 4)]-*O*-rhamnosyl-(1 → 4)-*O*-rhamnosyl-(1 → 2)-*O*-fucosyl [50]. The difference between these merremin compounds is found in the attachment position of the lactone aglycone. Macrocyclic aglycones of merremins A-D (**113**–**116**) are attached in the C-2 second sugar (1,2″-ester type). Meanwhile, merremin E (**117**) has its aglycone attached to the C-3 second sugar (1,3-ester type), with the presence of an acylic aglycone in the merremins F-G (**118**–**119**) structure.

**Figure 6:**
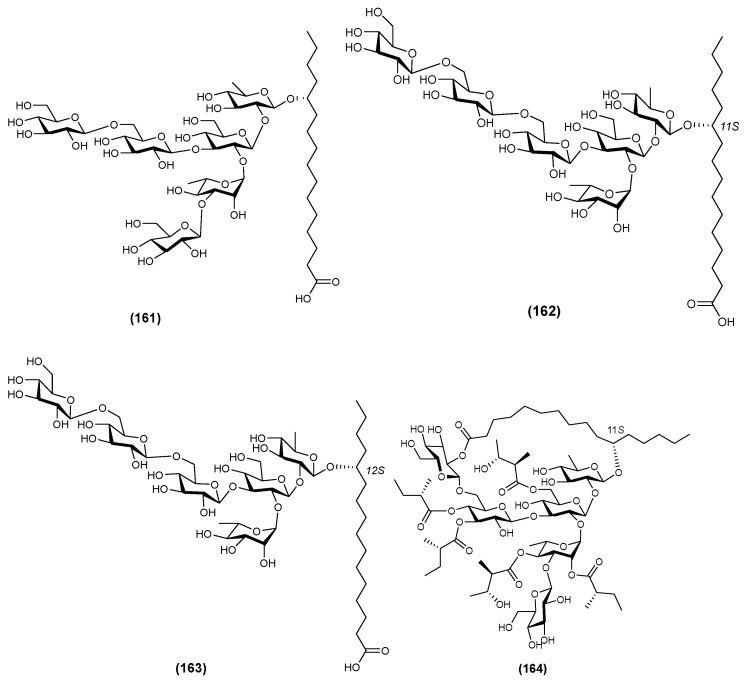

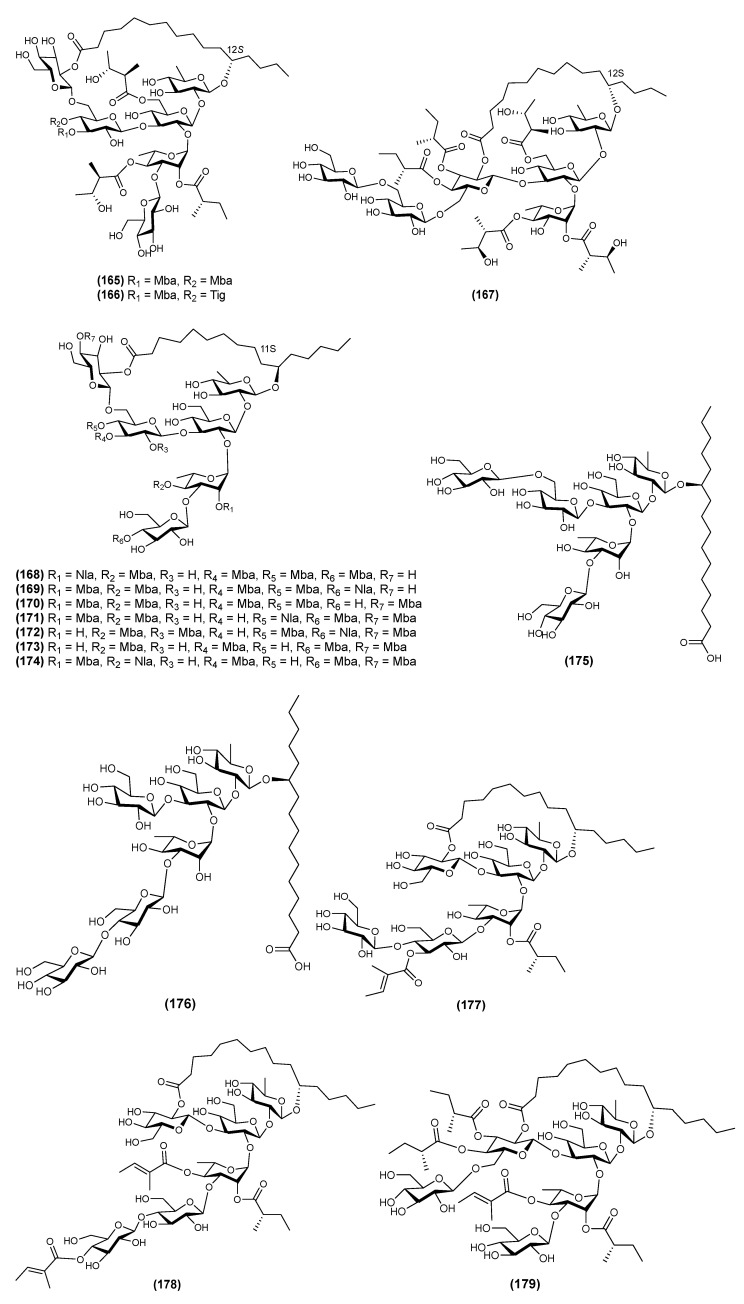

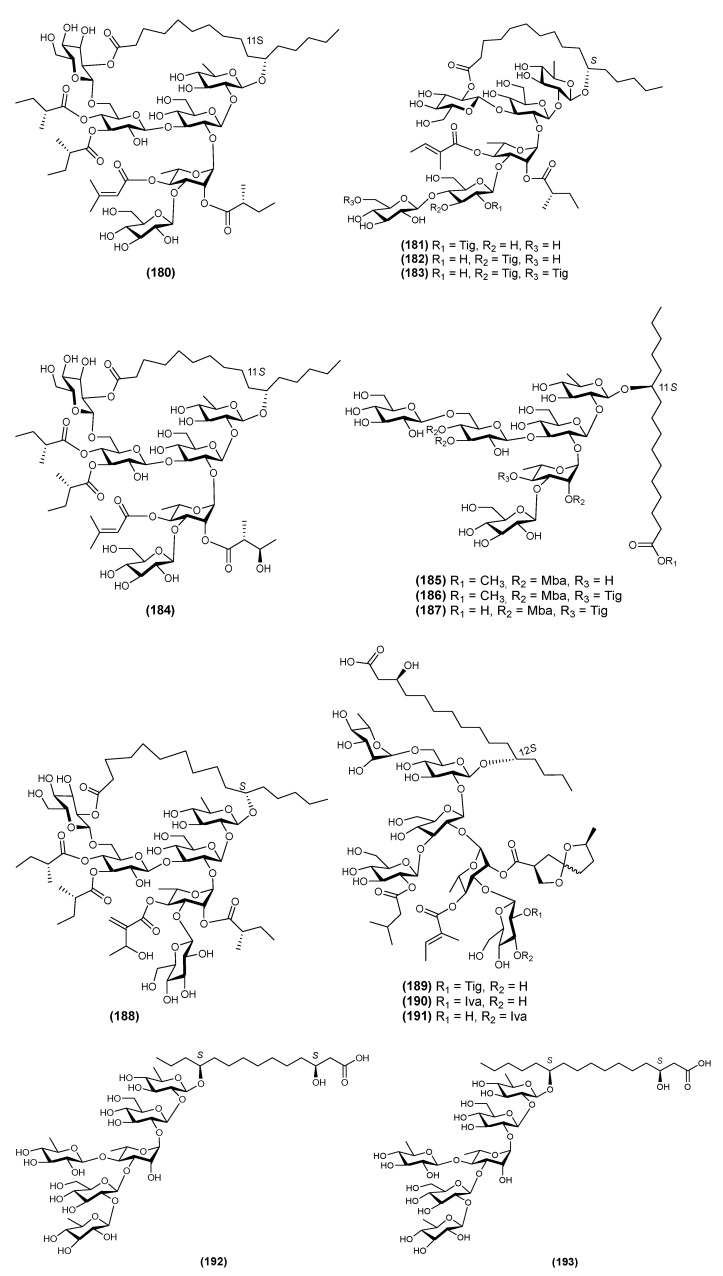

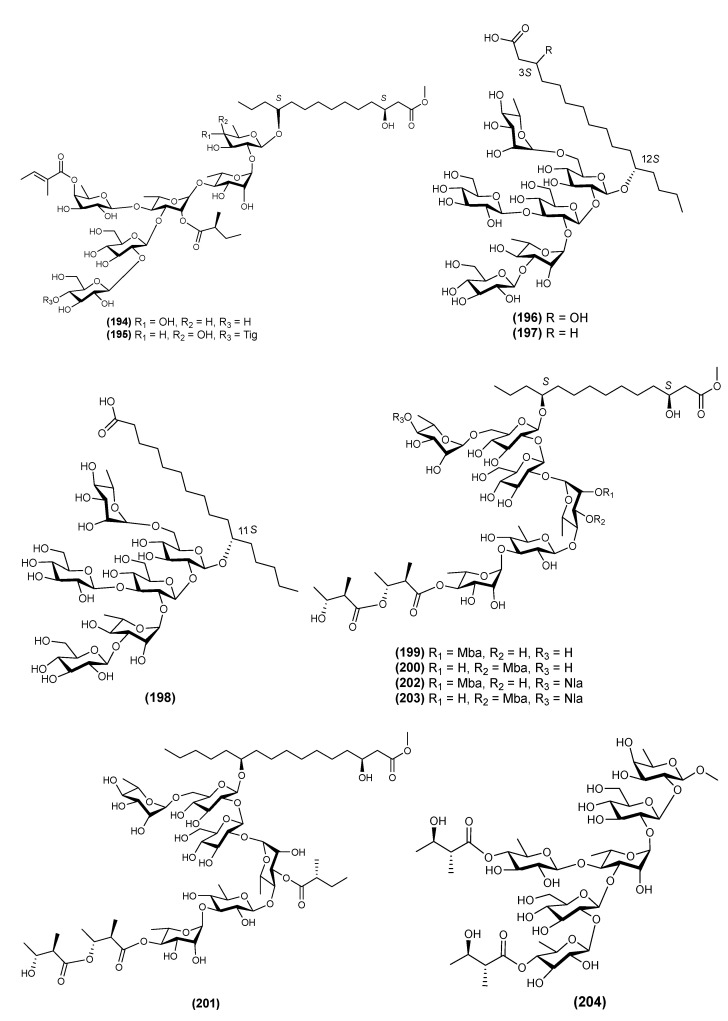

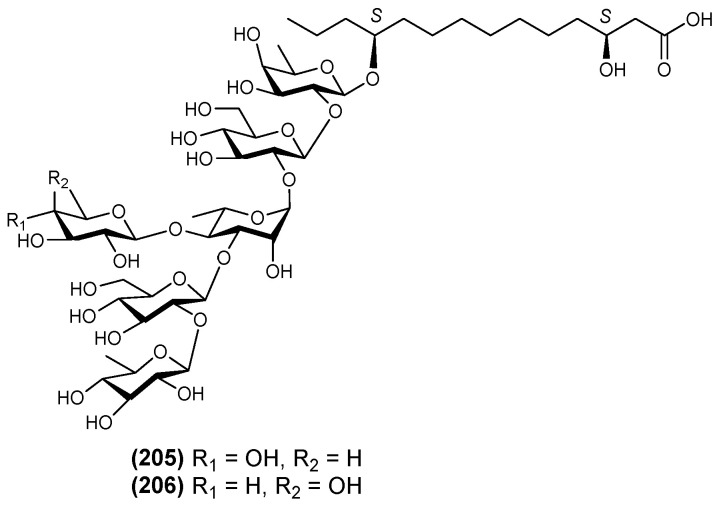
Structures of hexasaccharide-based resin glycosides **161**–**206**.

**Figure 7:**
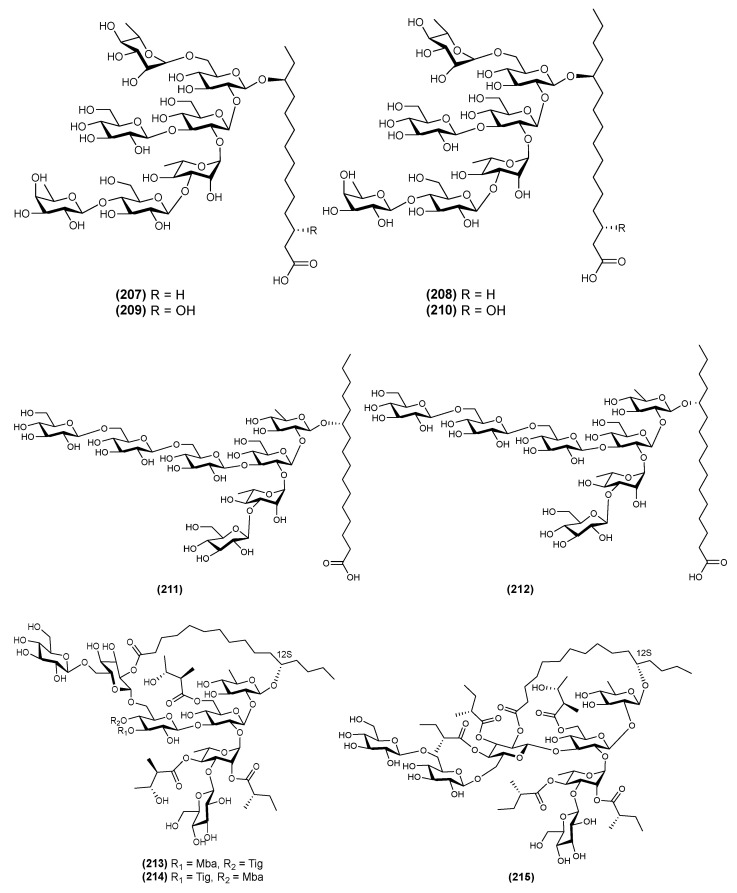

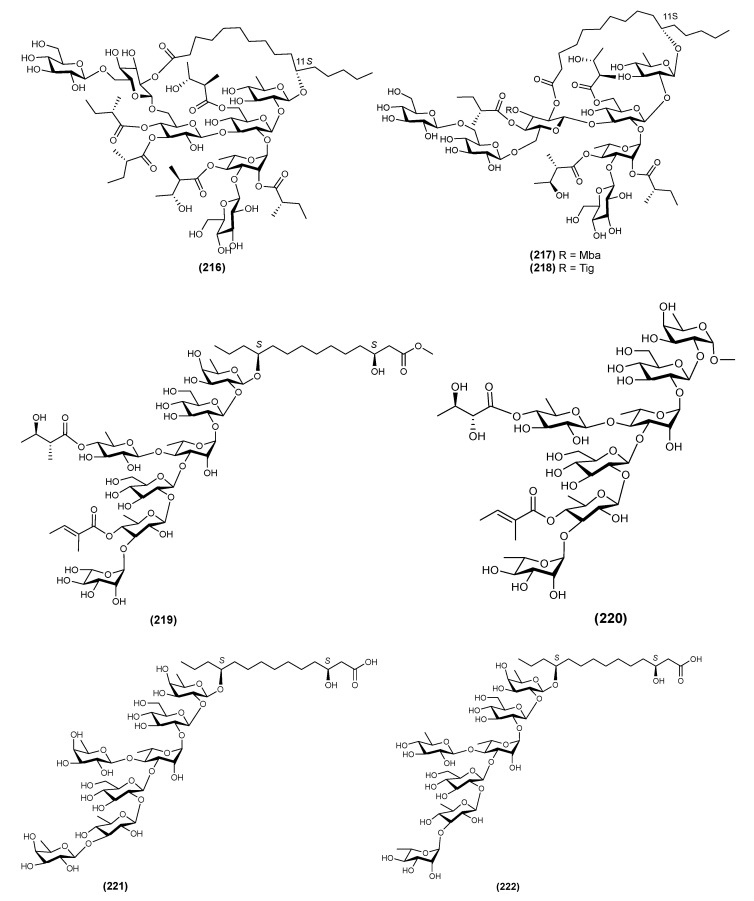
Structures of heptasaccharide-based resin glycosides **207**–**222**.

Seven resin glycosides have succeeded in isolation from *Quamoclit × multifida* plants [51]. These consist of four resin glycosides with a pentasaccharide core, multifidins III–VI (**120**–**123**) (Figure 5), two resin glycosides with a hexasaccharide core, multifidin VII–VIII (**184**–**185**) (Figure 6), and one type of the bidesmode group, multifidine IX (**218**) (Figure 7). Multifidins III–VI (**120**–**123**) compounds have two differences; firstly, the aglycone can be either macrocyclic lactone (ester-1,2″ type) for multifidin III–IV (**120**–**121**) or acyclic aglycone for multifidin V–VI (**122**–**122**). Secondly, there are two distinct oligosaccharide cores, namely, a glucosyl-(1 → 3)-*O*-[rhamnosyl-(1 → 4)]-*O*-rhamnosyl-(1 → 4)-*O*-rhamnosyl-(1 → 2)-*O*-fucopyranosyl for multifidin III (**120**) and multifidin V (**122**). This is in addition to a similar oligosaccharide core that differs in the first sugar unit, which is quinovose for multifidin IV (**121**) and multifidin VI (**123**).

The isolation of resin glycoside from *Ipomoea batatas* resulted in four murasakimasarins I–IV (**124**–**127**) (Figure 5) [52]. The difference in the structures of these compounds is in the first sugar unit, which built the oligosaccharide core. The first sugar unit in Murasakimasarin I (**124**) and murasakimasarin II–IV (**125**–**127**) are glucose and fucose, respectively. For example, murasakimasarin II (**125**) has an oligosaccharide core of glucosyl-(1 → 4)-rhamnosyl-(1 → 4)-rhamnosyl-(1 → 4)-rhamnosyl-(1 → 2)-fucosyl. The macrocyclic aglycone of murasakimasarins I–III (**124**–**126**) is attached to the C-2 of the second sugar unit (ester-1,2″ type). In contrast to murasakimasarin IV (**127**), its aglycone is attached to the C-3 of the second sugar unit (ester-1,3″ type).

A new glycosidic acid, named murucinic acid II (**128**) (Figure 5), with an oligosaccharide resin core of glucosyl-(1→2)-*O*-rhamnosyl-(1→4)-*O*-rhamnosyl-(1→4)-*O*-rhamnosyl-(1→2)-*O*-fucopyranoside was isolated from *Ipomoea murucoides* [16].

Ten resin glycosides, pescapreins XXI–XXX (**129**–**138**) (Figure 5), were found in *Ipomoea pes-caprae* [53]. The resins have frameworks of 11-*O*-α-L-rhamnopyranosyl-(1 → 3)-*O*-[α-L-rhamnopyranosyl-(1 → 4)]-*O*-α-L-rhamnopyranosyl-(1 → 4)-*O*-α-L-rhamnopyranosyl-(1 → 2)-*O*-β-D-fucopyranoside. The aglycone macrocyclic location, pescapreins XXI–XXVIII (**129**–**136**), is at the C-3 of the second sugar unit with an ester-1,3″ type. Meanwhile, pescapreins XXIX–XXX (**137–138**) has the aglycone attached to the C-2 of the second sugar unit (ester-1,2″ type).

The discovered resin glycosides in *Pharbitis nil* seeds have a unique acyclic aglycone, namely methyl 3*S*,11*S*-ipurotare in PM-6–PM-7 (**139–140**) (Figure 5) [54]. PM-6–PM-7 (**139**–**140**), which has a skeleton of quinosyl-(1 → 4)-rhamnosyl-(1 → 2)-*O*-glucosyl-(1 → 2)-*O*-[rhamnosyl-(1) oligosaccharides → 6)]-*O*-glucopyranosyl. The oligosaccharide sequence indicates that the aglycone is attached to the second sugar unit of glucose.

The pentasaccharide found in the *Ipomoea purga* plant, known as purginoside I–IV (**141**–**144**) (Figure 5) [6,9], have an oligosachharide core framework of glucosyl-(1 → 3)-*O*-[rhamnosyl-(1 → 4)]-*O*-rhamnosyl-(1 → 4)-*O*-rhamnosyl-(1 → 2)-*O*-fucopyranoside, which were confirmed to be related to the pescapreins and murucoidins series [74,75]. The macrocyclic aglycones of purginoside I–IV (**141**–**144**) are all attached to the C-2 of the second sugar, rhamnose (ester-1,2″).

The quamoclin V–VII (**145**–**147**) (Figure 5) isolated from *Quamoclit pennata* have a framework similar to purginoside, which is β-D-glc-(1 → 3)-*O*-[α-L-rha-(1 → 4)]-*O*-α-L-rha-(1 → 4)-*O*-α-L-rha-(1 → 2)-*O*-β-D-fuc [55]. The aglycones of the compounds differed from the structures of quamoclin V–VII (**145**–**147**). Similarly, Quamoclin V (**145**), quamoclin VI (**146**), and quamoclin VII (**147**) contain a 1,3″ ester-typed macrocyclic aglycone, 1,2″ ester-typed macrocyclic aglycon, and an acyclic aglycone. 

The *Operculina turpethum* plant produced glycosidic acids, consisting of turpethic acids A-C (**148**–**150**) (Figure 5), glycoside resin with lactone-1,2″ type, and turpethosides A-B (**151**–**152**) (Figure 5) [56]. The oligosaccharide core of these compounds is β-D-glucosyl-(1 → 3)-*O*-[α-L-rhamnosyl-(1 → 4)]-*O*-α-L-rhamnosyl-(1 → 4)-*O*-α-L-rhamnosyl-(1 → 2)-*O*-β-D-glucopyranoside. Meanwhile, the aglycones are unique because there are three variations of the fatty acids, namely, C15, C16, and C17. C15 fatty acids, such as 12*S*-hydroxypentadecanoic acid found in turpethic acid A (**148**) and turpethoside A (**151**). C16 fatty acids are found in turpethic acid B (**149**) and turpethoside (**152**) as a common aglycone of the resin glycosides, jalapinolic acid, and 12*S*-hydroxyhexadecanoic acid. Finally, C17 fatty acid and 12*S*-hydroxyheptadecanoic acid are found in turpethic acid C (**150**).

Four linear glycosidic acids, namely, tyrianthinoic acid (**153**) and tyrianthinic acids III–V (**154**–**156**) (Figure 5), have an oligosaccharide core of β-D-quinosyl-(1 → 4)-*O*-β-D-quinosyl-(1 → 4)-*O*-α-L-rhamnosyl-(1 → 2)-*O*-β-D-glucosyl-(1 → 2)-*O*-β-D-quinovopyranoside [17]. These compounds were isolated from *Ipomoea tyrianthina*.

The pentasaccharide core of wolcottinosides III–IV (**157**–**159**) (Figure 5) is glucosyl-(1 → 3)-*O*-[rhamnosyl-(1 → 4)]-*O*-rhamnosyl-(1 → 4)-*O*-rhamnosyl-(1 → 2)-*O*-glucopyranoside, which is almost similar to arboresins 1–6 with a difference in the last sugar unit (glc′) [24]. According to the report, the last sugar unit does not have a branch at C-3 of Rha″ but forms a linear pentasaccharide. Furthermore, wolcottinoside V (**158**) has a different oligosaccharide core of glucosyl-(1 → 3)-*O*-[rhamnosyl-(1 → 4)]-*O*-rhamnosyl-(1 → 4)-*O*-rhamnosyl-(1 → 2)-*O*-fucopyranoside, which is similar to intrapilosine VII (**112**). The macrolactonization of wolcottinosides III–V (**157**–**159**) took place at the C-2 of the second sugar unit. These three compounds were found in the plant *Ipomoea wolcottiana*.

The new resin glycoside compound (**160**) (Figure 5), isolated from the *Ipomoea maxima* plant, was named compound 1 by the first author [57]. Moreover, compound 1 (**160**) was reported to have an oligosaccharide core of rhamnosyl-(1 → 3)-*O*-[rhamnosyl-(1 → 4)]-*O*-rhamnosyl-(1 → 4)-*O*-rhamnosyl-(1 → 2)-*O*-fucopyranoside (ester-1.2″) similar to the aquaterins series [29]. 

### 2.5. Hexasaccharides

The plant of *Calystegia hederacea* produced resin glycosides containing hexasaccharide core, calyhedic acids, and calyhedins series [44,58,59,60]. Calyhedic acid B (**161**) (Figure 6) comprises β-D-glucosyl-(1 → 3)-*O*-α-L-rhamnosyl-(1 → 2)-[*O*-β-D-glucosyl-(1 → 6)-*O*-β-D-glucosyl-(1 → 3)]-*O*-β-D-glucosyl-(1 → 2)-*O*-β-D-quinovopyranoside with acyclic aglycone 12*S*-hydroxyhexadecanoic acid [44]. This acid has similar sugar units with calyhedic acid E-F (**162**–**163**) (Figure 6), namely four glucose, one rhamnose, and one quinovose. However, in calyhedic acid E-F (**162**–**163**)[58], one glucose sugar unit changed position and was not bound to C-3 units of rhamnose sugar but was bound to C-6 glucose units. Hence, the hexasaccharide core became α-L-rhamnosyl-(1 → 2)-[*O*-β-D-glucosyl-(1 → 6)-*O*-β-D-glucosyl-(1 → 6)-*O*-β-D-glucosyl-(1 → 3)]-*O*-β-D-glucosyl-(1 → 2)-*O*-β-D-quinovopyranoside. The difference in the position of the hydroxyl group in the aglycone is between these two compounds. The acyclic aglycone of 11*S*-hydroxyhexadecanoic and 12*S*-hydroxyhexadecanoic acids are found in calyhedic acid E (**162**) and calyhedic acid F (**163**), respectively. The macrocyclic form of calyhedic acids is the calyhedin series. Calyhedins I–III (**164**–**166**) (Figure 6) have the same hexasaccharide core as calyhedic acid B (**161**). The macrolactonization sites of these three compounds are located at the C-2 position of the glc sugar unit. Calyhedin I (**164**) and calyhedins II–III (**165**–**166**) have aglycone of 11*S*-hydroxyhexadecanoic acid and 12*S*-hydroxyhexadecanoic acid [59]. Calyhedin X (**167**) (Figure 6) [60] has the same hexasaccharide core as calyhedic acid F (**163**). The difference is that calyhedin X (**166**) is a macrolactone aglycone bound to the C-2 sugar unit of Glc.

Calysepins I–VII (**168**–**174**) (Figure 6) has a structural skeleton of β-D-glucopyranosyl-(1 → 3)-*O*-α-L-rhamnopyranosyl-(1 → 2)-[*O*-β-D-glucopyranosyl-(1 → 6)-*O*-β-D-glucopyranosyl-(1 → 3)]-*O*-β-D-glucopyranosyl-(1 → 2)-α-D-quinovopyranoside [61]. The aglycone of 11*S*-jalpinolic acid forms a lactone that is attached to the C-2 of the glucose (glc‴). The difference between the calysepins I–VII (**168**–**174**) is in the variety of organic acids, namely methyl butyric acid (Mba) and Nilic acid (Nla).

In the plant of *Calystegia soldanella*, a series of hexasaccharide-typed resin glycosides, calysolic acids, and calysolins were isolated. Calysolic acids C-D (**175**–**176**) (Figure 6) are isolated as glycosidic acid with the aglycone of 11*S*-hydroxyhexadecanoic acid. Calysolic acid C (**175**) has the same oligosaccharide core as calyhedic acid B (**161**). In contrast to calysolic acid D (**176**), the sugar unit attached to C-6 Glc′ moves to C-4 Glc‴, hence the skeleton becomes β-D-glucosyl-(1 → 4)-*O*-β-D-glucosyl-(1 → 3)-α-L-rhamnosyl-(1 → 2)-[*O*-β-D-glucosyl-(1 → 3)]-*O*-β-D-glucosyl-(1 → 2)-*O*-β-D-quinovopyranoside [45]. Calysolin IV (**177**) (Figure 6) has the same hexasaccharide framework as calysolic acid D (**176**). In contrast, the aglycone of calysolin IV (**177**) forms a cyclic structure at C-2 of the Glc′ sugar unit [30]. Calysolins series with a hexasaccharide core, namely calysolins VII–IX (**178**–**180**) (Figure 6), were isolated [46]. Calysolin VII (**178**) has an oligosaccharide core with a similar aglycone location as calysolin IV (**177**). Meanwhile, calysolin VIII–IX (**179**–**180**) has the same hexasaccharide core as calysolic acid C (**175**) and calyhedic acid B (**161**) with a varying location. Macrolactonization took place at C-2 sugar units Glc′ for calysolin VIII (**179**) and at C-2 sugar units Glc″ for calysolin IX (**180**). Calysolins XI–XIII (**181**–**183**) (Figure 6) [31] are an advanced series of calysolins, with the same hexasaccharide core as calysolin IV (**177**) and VII (**178**). The difference between these compounds is in the variety of organic acids. In the following year, calysolins XIV–XVII (**184**–**187**) (Figure 6) were isolated to obtain a structural skeleton of glucosyl-(1 → 3)-*O*-rhamnosyl-(1 → 2)-[*O*-glucosyl-(1 → 6)-*O*-glucosyl-(1 → 6)-*O*-glucosyl-(1 → 6)-*O*-glucosyl-(1 → 6)-(1 → 3)]-*O*-glucosyl-(1 → 2)-*O*-quinovopyranoside [62]. This skeleton is similar to the hexasaccharide core of calysolin VIII–IX (**179**–**180**). Calysolin XIV (**184**) has the same macrolactone aglycone of C-2 sugar units Glc″‴ as calysolin IX (**180**). Meanwhile, calysolins XV–XVII (**185**–**187**) are resin glycosides with acyclic aglycones [62]. The last series of calysolin are calysolin XIII (**188**) (Figure 6) isolated from the leaves, stems, and roots of *C. soldanella* [14]. There was no difference in the hexasaccharide core compared to the previous calysolins VIII–IX (**179**–**180**) and XIV–XVII (**184**–**187**). The macrolactone was built at the C-2 sugar unit Glc″‴.

Resin glycoside of macrocarposidic acids A-C (**189**–**191**) (Figure 6) has a unique hexasaccharide core of β-D-glucosyl-(1 → 3)-*O*-α-L-rhamnosyl-(1 → 2)-[*O*-β-D-glucosyl-(1 → 3)]-*O*-β-D-glucosyl-(1 → 2)-[*O*-α-L-rhamnosyl-(1 → 6)]-*O*-β-D-glucopyranoside [28]. These resin glycosides have similarities in their acyclic aglycone, 3S,12*S*-dihydroxyhexadecanoic acid, and the presence of exogonoyl organic acids. The difference between the structures is in the presence of isovaleroyl (Iva) and tigloyl (Tig) organic acids at C-2 and C-3 of Glc‴ sugar units. Macrocarposidic acids A-C (**189**–**191**) are isolated from the plant of *Operculina macrocarpa*.

*Quamoclit × multifida* produced hexasaccharide-based resin, maltifidinic acids C-D (**192**–**193**) (Figure 6) [26]. Both compounds have an oligosaccharide core of β-D-quinovopyranosyl-(1 → 2)-*O*-β-D-glucopyranosyl-(1 → 3)-[*O*-β-D-fucopyranosyl-(1 → 4)]-*O*-α-L-rhamnopyranosyl-(1 → 2)-*O*-β-D-glucopyranosyl-(1 → 2)-*O*-β-D-quinovopyranoside and categorized to glycosidic acid, with two hydroxyl positions at C-3 and C-11 on the aglycone. Maltifidinic acids C-D (**192**–**193**) are homologous compounds, and based on HR-Positive Ion-FAB-MS, maltifidinic acid D (**193**) has a mass unit of 28 (C_2_H_4_) larger than maltifidinic acid C (**192**). Therefore, the difference lies in their aglycone. ^1^H- and ^13^C-NMR analysis and the determination of absolute configuration using Mosher method with MTPA reagent revealed that maltifidinic acid C (**192**) is 3*S*,11*S*-dihydroxytetradecanoic acid, 3*S*,11*S*-convolvulinic acid or 3*S*,11*S*-ipurolic acid, while maltifidinic acid D (**193**) is 3*S*,11*S*-dihydroxyhexadecanoic acid. Another hexasaccharide glycosidic acids, multifidins VII–VIII (**194**–**195**) (Figure 6), were found in *Q. multifida* [51]. Multifidin VII (**194**) has a similar oligosaccharide core as maltifidinic acid C-D (**192**–**193**), as opposed to multifidin VIII (**195**), which has a different first sugar unit in the form of fucose. The hexasaccharide core of multifidin VIII (**195**) becomes quinosyl-(1 → 2)-*O*-glucosyl-(1 → 3)-[*O*-fucosyl-(1 → 4)]-*O*-rhamnosyl-(1 → 2)-*O*-glucosyl-(1 → 2)-*O*-fucopyranoside. The aglycones of these two compounds are 3*S* and 11*S*-ipurolic acid.

Operculinic acids H-J (**196**–**198**) (Figure 6) are glycosidic acids obtained from the roots of *O. macrocarpa* [28]. Structurally, the hexasaccharide core of these two compounds is similar to macrocarposidic acids A-C (**189**–**191**). The hydroxy group in the aglycone of operculinic acid H-I (**196**–**197**) is 12*S*-hydroxyhexadecanoic acid, and 11*S*-hydroxyhexadecanoic acid found in operculinic acid J (**198**).

The acylated hydroxy of fatty acids was found in the seeds of *Pharbitis nil*, PM1–PM5 (**199**–**203**) (Figure 6) [54]. The framework for these compounds is α-L-rhamnosyl-(1 → 3)-*O*-β-D-quinosyl-(1 → 4)-α-L-rhamnosyl-(1 → 2)-*O*-β-D-glucosyl-(1 → 2)-[*O*-α-L-rhamnosyl-(1 → 6)]-*O*-β-D-glucopyranoside, which is a linear hexasaccharide that differs from the other series of hexasaccharide-typed resin glycosides. The aglycone of PM3 (**201**) is methyl 3*S*,11*S*-dihydroxyhexadecanoate. Meanwhile, PM1–PM2 (**199**–**200**) and PM4–PM5 (**202**–**203**) have an aglycone of methyl 3*S*,11*S*-dihydroxytetradecanoate.

The new of methyl pyranoside was discovered in the *Quamoclit pennata* plant, namely, QM4 (**204**) (Figure 6) [63]. In this compound, the common aglycone of 11*S*-jalapinolic acid and 11*S*-convolvulic acid in the resin glycoside is replaced by methyl groups. The hexasaccharide framework of this compound is β-D-quinosyl-(1 → 2)-*O*-β-D-glucosyl-(1 → 3)-*O*-[β-D-quinosyl-(1 → 4)]-*O*-α-L-rhamnosyl-(1 → 2)-*O*-β-D-glucosyl-(1 → 2)-α-D-fucopyranoside. Another resin glycoside found in *Q. pennata* is quamoclinic acids C-D (**205**–**206**) (Figure 6), which contain dihydroxy fatty acids, 3S,11*S*-ipurolic acid [27]. The oligosaccharide core of quamoclinic acid C (**205**) is similar to QM4 (**204**), while in quamoclinic acid D (**206**), the quinovose sugar unit attached to C-4 of the Rha sugar group is replaced with a fucose sugar unit. The hexasaccharide core of quamoclinic acid D (**206**) becomes β-D-quinosyl-(1 → 2)-*O*-β-D-glucosyl-(1 → 3)-*O*-[β-D-fucosyl-(1 → 4)]-*O*-α-L-rhamnosyl-(1 → 2)-*O*-β-D-glucosyl-(1 → 2)-*O*-β-D-fucopyranoside.

### 2.6. Heptasaccharides

Four heptasaccharide-typed resin glycosides were found in the plant of *Convolvulus arvensis*, namely, arvensis A-D (**207**–**210**) (Figure 7) [64]. The oligosaccharide core of these compounds is β-D-fucopyranosyl-(1 → 4)-*O*-β-D-glucopyranosyl-(1 → 3)-*O*-α-L-rhamnopyranosyl-(1 → 2)-[*O*-β-D-glucopyranosyl-(1 → 3)]-*O*-β-D-glucopyranosyl-(1 → 2)-[*O*-α-L-rhampyranosyl-(1 → 6)]-*O*-β-D-glucopyranoside, which is similar to the hexasaccharide core of PM1–PM5 (**199**–**203**). However, a fucose unit is added to the last sugar unit of arvensis A-D (**207**–**210**). There are two variations of acyclic aglycones in these four compounds, firstly, 12*S*-pentadecanoic acid for arvensis A (**207**) and C (**209**), and secondly, 12*S*-hexadecanoic acid for arvensis B (**208**) and D (**210**).

The plant *Calystegia hederacae* produced a series of heptasaccharide-type resin glycosides [44,59,60]. Calyhedic acids C-D (**211**–**212**) (Figure 7) have a similar heptasaccharide core, namely, 11-*O*-D-glucosyl-(1 → 3)-*O*-α-L-rhamnosyl-(1 → 2)-[*O*-β-D-glucosyl-(1 → 6)-*O*-β-D-glucosyl-(1 → 6)-*O*-β-D-glucosyl-(1 → 3)]-*O*-β-D-glucosyl-(1 → 2)-*O*-β-D-quinovopyranoside. Acyclic aglycones distinguish 11*S*-hydroxyhexadecanoic and 12*S*-hydroxyhexadecanoic acids for calyhedic acid C (**211**) and calyhedic acid D (**212**), respectively [44]. Three heptasaccharide-type resin glycoside compounds, namely, calyhedins IV–VI (**213**–**215**) (Figure 7) [59]. The heptasaccharide core of these three compounds is similar to calyhedic acids C-D (**211**–**212**). The aglycones of calyhedic acids IV–V (**213**–**214**) form an intramolecular ester bond with glc‴ (1,2‴-ester type), while calyhedic acid VI (**215**) forms lactone bonds with glc′ (1,2′-ester type). The most recent findings of the heptasaccharides-based calyhedins series from *C. hederacea* are calyhedins VII–IX (**216**–**218**) (Figure 7) [60]. Structurally, there is no significant difference between the heptasaccharide cores of calyhedins VII–IX (**216**–**218**), calyhedins IV–VI (**213**–**215**), and calyhedic acids C-D (**211**–**212**), but the difference is in the location of the intramolecular ester. Calyhedins VII–IX (**216**–**218**) have two types of rings, a 27-membered ring for calyhedins VII (**216**) and a 22-membered ring for calyhedins VIII–IX (**217**–**218**).

Heptasaccharides-type resin glycosides with a series of QMs and quamoclinic acid were isolated from *Quamoclit pennata* [27,63,65]. QM1 (**219**) and QM7 (**220**) (Figure 7) have a heptasaccharide core that is almost similar to the heptasaccharide of maltifidinic acid C-D (**192**–**193**). However, there is an addition of one more sugar unit, rha″, at the end of the oligosaccharide, giving a framework of α-L-rhamnopyranosyl-(1 → 3)-*O*-β-D-quinovopyranosyl-(1 → 2)-*O*-β-D-glucopyranosyl-(1 → 3)-[*O*-β-D-quinovopyranosyl-(1 → 4)]-*O*-α-L-rhamnopyranosyl-(1 → 2)-*O*-β-D-glucopyranosyl-(1 → 2)-*O*-β-D-fucopyranoside. The aglycone of QM1 (**219**) is determined as methyl 3*S*,11*S*-dihydroxytetradecanoate [63], while QM7 (**220**) is only methyl [65]. Another series of the heptasaccharide resin, namely quamoclinic acid E-F (**221**–**222**) (Figure 7), has the same aglycone moiety with 3*S*,11*S*-ipurolic acid, and 3*S*,11*S*-dihydroxytetradecanoic acid [27]. However, the heptasaccharide core of both resin glycosides is different. Quamoclinic acid F (**222**) has a similar oligosaccharide core to QM1 (**219**), as opposed to quamoclinic E (**221**), in which the two sugar units, rhamnose, and quinovose, are replaced by fucose resulting in the core of β-D-fucopyranosyl-(1 → 3)-*O*-β-D-quinovopyranosyl-(1 → 2)-*O*-β-D-glucopyranosyl-(1 → 3)-[*O*-β-D-fucopyranosyl-(1 → 4)]-*O*-α-L-rhamnopyranosyl-(1 → 2)-*O*-β-D-glucopyranosyl-(1 → 2)-*O*-β-D-fucopyranoside.

### 2.7. Bidesmosides

**Figure 8:**
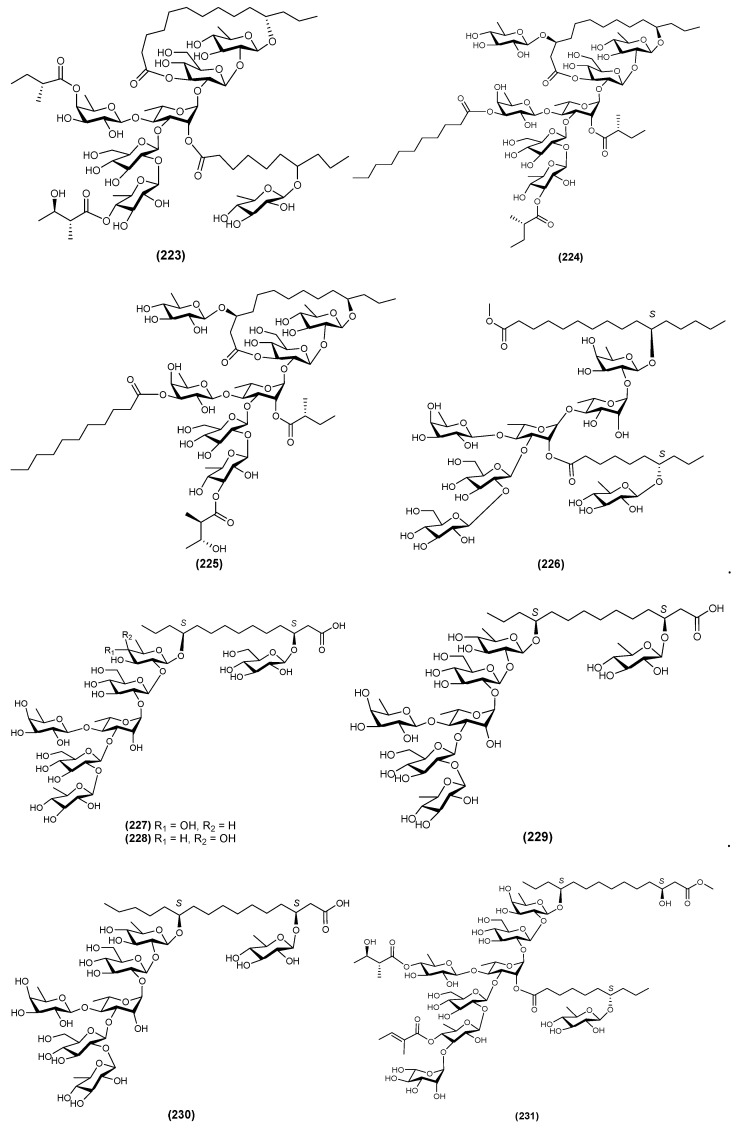

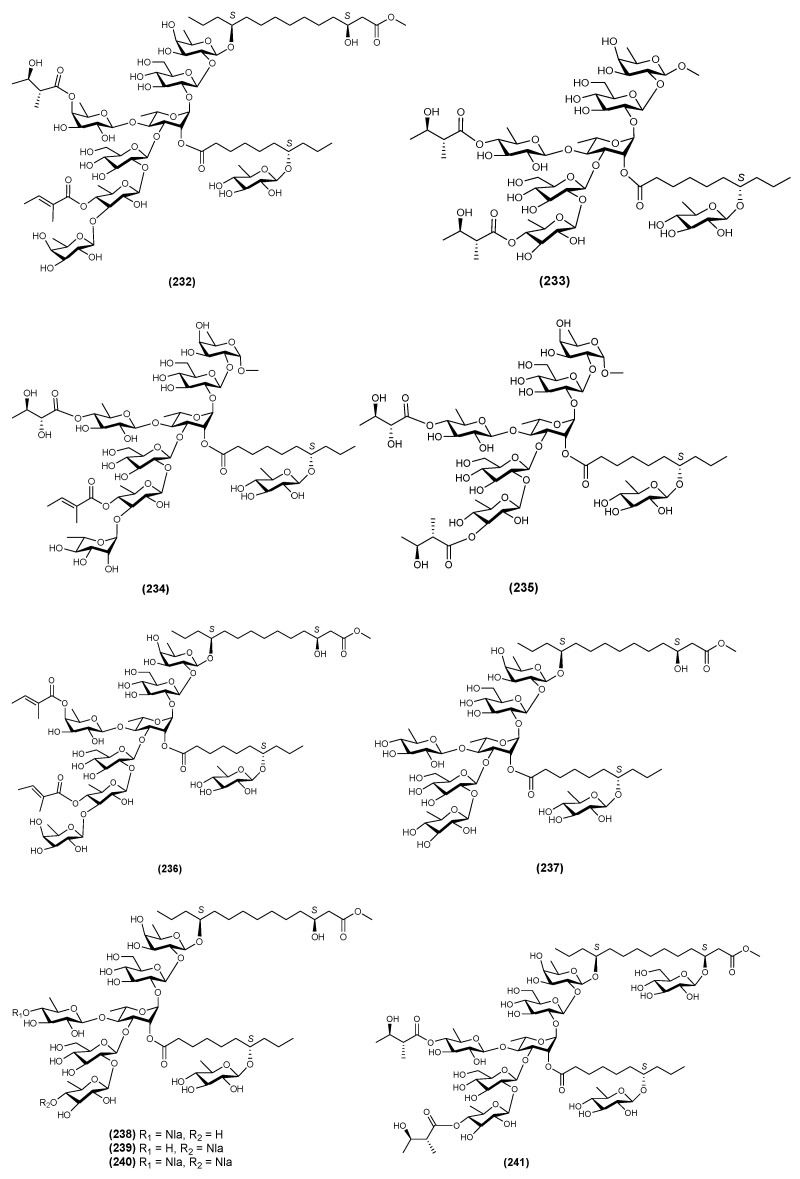

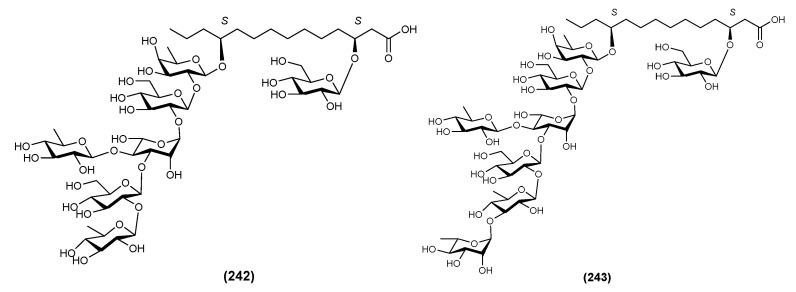
Structures of bidesmoside-based resin glycosides **223**–**243**.

Jalapinoside B (**223**) (Figure 8) was isolated from the methanolic extract of the roots of *Ipomoea purga* [16]. Structurally, this compound is categorized as resin glycoside in the macrocylic bidesmoside class and is characterized by the presence of additional sugar units attached to the aglycone outside the main framework of the oligosaccharide core. This core is similar to the hexasaccharide of multifidin VII (**194**). Jalapinoside B (**223**) contains two aglycones and one macrocyclic aglycone 11*S*-convolvulinic acid (1,3″-lactone) attached to the skeleton of the main oligosaccharide. The second aglycone is an aglycone placed outside the oligosaccharide framework, which has an additional sugar unit of quinovose at the decanoic acid (C-7), which is structurally similar to the monosaccharide-type resin glycoside, quamoclinic acid B (**2**). Therefore, the framework to the jalapinoside B (**223**) is 11*S*-hydroxytetradecanoic acid 11-*O*-β-D-quinovopyranosyl-(1 → 2)-*O*-β-D-glucopyranosyl-(1 → 3)-[*O*-β-D-fucopyranosyl-(1 → 4)-(2-*O*-7*S*-hydroxydecanoic acid-*O*-β-D-quinovopyranosyl-(1 → 2)]-*O*-α-L-rhamnopyranosyl-(1 → 2)-*O*-β-D-glucopyranosyl-(1 → 2)-*O*-β-D-quinovopyranoside (1,3″-lactone). Jalapinosides I–II (**224**–**225**) (Figure 8) were reportedly isolated [66,67]. These compounds have a shared similar oligosaccharide framework with jalapinoside B (**223**). However, an additional quinovose unit is not attached to the decanoic acid, unlike the 3*S*,11*S*-ipurolic acid (C-3). Jalapinoside I–II (**224**–**225**) has a structural framework of 3*S*,11*S*-hydroxytetradecanoic acid 11-*O*-β-D-quinovopyranosyl-(1 → 2)-*O*-β-D-glucopyranosyl-(1 → 3)-[*O*-β-D-fucopyranosyl-(1 → 4)]-*O*-α-L-rhamnopyranosyl-(1 → 2)-*O*-β-D-glucopyranosyl-(1 → 2)-*O*-β-D-quinovopyranosyloxy-3-[*O*-β-D-quinovopyranosyloxy]-1,3″-lactone.

A resin glycoside with a macrocylic bidesmoside framework, named multifidin IX (**226**) (Figure 8), was isolated from *Quamoclit × multifida* [51]. This compound has a similar oligosaccharide core as multifidin VIII (**195**) with additional sugar units in the form of quamoclinic acid B (**2**) and the acyclic aglycone of methyl 3*S*,11*S*-ipurolate. Its structural skeleton is methyl 3*S*,11*S*-ipurolate 11-*O*-β-D-quinovopyranosyl-(1 → 2)-*O*-β-D-glucopyranosyl-(1 → 3)-[*O*-β-D-fucopyranosyl-(1 → 4)]-(2-*O*-7*S*-hydroxydecanoic acid 7-*O*-β-D-quinovopyranosyl-(1 → 2)]-*O*-α-L-rhamnopyranosyl-(1 → 2)-*O*-β-D-glucopyranosyl-(1 → 2)-*O*-β-D-fucopyranoside. Other macrocylic bidesmoside-type resin glycosides that were isolated from this plant included multifidinic acids F-G (**227**–**228**) (Figure 8) [68]. Multifidinic acid F (**227**) has an oligosaccharide core, which is similar to the hexasaccharide core of multifidin VII (**194**), and the additional glucose unit attached to the 3*S*,11*S*-ipurolic acid (C-3), meaning that the structural skeleton of 3*S*,11*S*-hydroxytetradecanoic acid can be given as 11-*O*-β-D-quinovopyranosyl-(1 → 2)-*O*-β-D-glucopyranosyl-(1 → 3)-[*O*-β-D-fucopyranosyl-(1 → 4)]-*O*-α-L-rhamnopyranosyl-(1 → 2)-*O*-β-D-glucopyranosyl-(1 → 2)-*O*-β-D-quinovopyranosyl-3-*O*-β-D-glucopyranoside. Multifidinic acid G (**228**) shares a similar oligosaccharide core as multifidin VIII (**195**), with an additional glucose unit attached to the 3*S*,11*S*-ipurolic acid (C-3), revealing the structural skeleton to be 3*S*,11*S*-hydroxytetradecanoic acid 11-*O*-β-D-quinovopyranosyl-(1 → 2)-*O*-β-D-glucopyranosyl-(1 → 3)-[*O*-β-D-fucopyranosyl-(1 → 4)]-*O*-α-L-rhamnopyranosyl-(1 → 2)-*O*-β-D-glucopyranosyl-(1 → 2)-*O*-β-D-fucopyranosyl-3-*O*-β-D-glucopyranoside.

Purgic acids C-D (**229**–**230**) (Figure 8) are a series of bidesmosidic-type resin glycosides found in *Ipomoea purga* [67]. The main oligosaccharide core of purgic acid C (**229**) is similar to the hexasaccharide core of multifidin VII (**194**) with an acyclic aglycone, 3*S*,11*S*-hydroxytetradecanoic acid. Structurally, the compound is similar to that of multifidinic acid F (**227**), but a bidemosidic moiety is a quinovose unit attached to the dihydroxy aglycone (C3). This leads to the structural framework of purgic acid C (**229**), which is 11-*O*-β-D-quinovopyranosyl-(1 → 2)-*O*-β-D-glucopyranosyl-(1 → 3)-[*O*-β-D-fucopyranosyl-(1 → 4)]-*O*-α-L-rhamnopyranosyl-(1 → 2)-*O*-β-D-glucopyranosyl-(1 → 2)-*O*-β-D-quinovopyranosyl-3*S*,11*S*-hydroxytetradecanoic acid 3-*O*-β-D-quinovopyranoside. Purgic acid D (**230**) has a similar oligosaccharide core and bidesmosidic sugar units as purgic acid C (**229**). However, the aglycone of this compound is 3*S*,11*S*-hydroxyhexadecanoic acid, hence the skeleton is 11-*O*-β-D-quinovopyranosyl-(1 → 2)-*O*-β-D-glucopyranosyl-(1 → 3)-[*O*-β-D-fucopyranosyl-(1 → 4)]-*O*-α-L-rhamnopyranosyl-(1 → 2)-*O*-β-D-glucopyranosyl-(1 → 2)-*O*-β-D-quinovopyranoside-3*S*,11*S*-hydroxyhexadecanoic acid 3-*O*-β-D-quinovopyranoside.

Most macrocylic bidemoside-types of resin glycosides are found in the QMs series of *Quamoclit pennata* plants [63,65,69]. These QMs are classified as bidemosides because the structures have one additional sugar unit, quamoclinic acid B (**2**), which is commonly attached to the C-2 unit of the rhamnose sugar. QM2 (**231**) (Figure 8) has a heptasaccharide core and aglycones similar to QM1 (**219**). However, QM2 (**231**) has a bidemosidic part that is absent in QM1 (**219**), while QM2 (**231**) has a fully structural skeleton which is given as methyl 3*S*,11*S*-dihydroxytetradecanoate 11-*O*-α-L-rhamnopyranosyl-(1 → 3)-*O*-β-D-quinovopyranosyl-(1 → 2)-*O*-β-D-glucopyranosyl-(1 → 3)-[*O*-β-D-quinovopyranoside-(1 → 4)]-*O*-(2-*O*-7*S*-hydroxydecanoic acid 7-*O*-β-D-quinovopyranoside))-α-L-rhamnopyranosyl-(1 → 2)-*O*-β-D-glucopyranosyl-(1 → 2)-*O*-β-D-fucopyranoside. QM3 (**232**) (Figure 8) has a similar heptasaccharide core as quamoclinic acid E (**221**) with a bidesmoside part of quamoclinic acid B (**2**) and an aglycone of methyl 3*S*,11*S*-ipurolate in QM3 (**232**), giving a structural framework of 3*S*,11*S*-dihydroxytetradecanoate 11-*O*-β-D-fucopyranosyl-(1 → 3)-*O*-β-D-quinovopyranosyl-(1 → 2)-*O*-β-D-glucopyranosyl-(1 → 3)-[*O*-β-D-fucopyranosyl-(1 → 4)]-*O*-(2-*O*-7*S*-hydroxydecanoic acid 7-*O*-β-D-quinovopyranoside))-α-L-rhamnopyranosyl-(1 → 2)-*O*-β-D-glucopyranosyl-(1 → 2)-*O*-β-D-fucopyranoside. QM5 (**233**) (Figure 8) has a similar hexasaccharide core to QM4 (**204**), which comprises a resin without an aglycone but a methyl group QM5 (**233**). It has a quamoclinic acid B (**2**) as a bidemosidic moiety to obtain methyl-D-quinovopyranosyl-(1 → 3)-*O*-β-D-quinovopyranosyl-(1 → 2)-*O*-β-D-glucopyranosyl-(1 → 3)-[*O*-β-D-quinovopyranosyl-(1 → 4)]-*O*-(2-*O*-7*S*-hydroxydecanoic acid 7-*O*-β-D-quinovopyranosyl-(1 → 2))-α-L-rhamnopyranosyl-(1 → 2)-*O*-β-D-glucopyranosyl-(1 → 2)-*O*-β-D-fucopyranoside as its skeleton [63]. QM8 (**235**) (Figure 8) [65] has a similar structure to QM5 (**233**), but QM8 (**235**) contains α oriented methyl group, while QM5 (**233**) is in the β-oriented methyl group. Methylated glycoside is present in the QM6 (**234**) (Figure 8), which has the structural skeleton of a heptasaccharide core, similar to the α-oriented methyl group of QM7 (**220**). In QM6 (**234**), there is a bidemosidic quamoclinic acid B (**2**); hence, the framework becomes methyl-L-rhamnosyl-(1 → 3)-*O*-β-D-quinosyl-(1 → 2)-*O*-β-D-glucosyl-(1 → 3)-[*O*-β-D-quinosyl-(1 → 4)]-*O*-(2-*O*-7*S*-hydroxydecanoic acid 7-*O*-β-D-quinosyl-(1 → 2)-α-L-rhamnosyl-(1 → 2)-*O*-β-D-glucosyl-(1 → 2)-*O*-β-D-fucopyranoside [65]. The heptasaccharide core, bidemosidic moiety, and aglycone of QM9 (**236**) (Figure 8), are similar to QM3 (**232**), which is methyl 3*S*,11*S*-dihydroxytetradecanoate 11-*O*-β-D-Fuc-(1 → 3)-*O*-β-D-Qui-(1 → 2)-*O*-β-D-Glc-(1 → 3)-[*O*-β-D-Fuc-(1 → 4)]-*O*-(2-*O*-7*S*-hydroxydecanoic acid 7-*O*-β-D-Qui)-α-L-Rha-(1 → 2)-*O*-β-D-Glc-(1 → 2)-*O*-β-D-Fuc. The difference between QM9 (**236**) and QM3 (**232**) is in the organic acid group attached to the C-4 Fuc′ sugar unit, in which nilic acid (Nla) and tiglic acid (Tig) are found in QM3 (**232**) and QM9 (**236**), respectively. QM10 (**237**) (Figure 8) has a similar hexasaccharide core and bidesmosidic moiety as QM5 (**233**). However, its methyl ipurolate aglycone provides a fully structural skeleton as methyl 3*S*,11*S*-dihyroxytetradecanoate 11-*O*-β-D-Qui-(1 → 3)-*O*-β-D-Qui-(1 → 2)-*O*-β-D-Glc-(1 → 3)-[*O*-β-D-Qui-(1 → 4)]-*O*-(2-*O*-7*S*-hydroxydecanoic acid 7-*O*-β-D-Qui-(1 → 2))-α-L-Rha-(1 → 2)-*O*-β-D-Glc-(1 → 2)-*O*-β-D-Fuc [65]. Another series of QMs resin glycosides are QM11–QM13 (**238**–**240**) (Figure 8) [69], which have a similar structural framework to QM10 (**237**). The difference between QM11 and QM13 (**238**–**240**) lies in the variation of the position of the organic nilic acid (Nla). QM14 (**241**) (Figure 8) was the last series of QM compounds found in *Q. pennata*. Structurally, the oligosaccharide core is similar to QM13 (**240**). Meanwhile, the structural characteristic of QM14 (**241**) is the presence of two bidesmosidic parts, quamoclinic acid B (**2**), which is common in the QMs series, and an additional glucose unit attached to C-3 methyl ipurolate as a second bidemosidic. Therefore, the skeleton of this compound is identified as 11-*O*-β-D-quinovopyranosyl-(1 → 3)-*O*-β-D-quinovopyranosyl-(1 → 2)-*O*-β-D-glucopyranosyl-(1 → 3)-[*O*-β-D-quinovopyranosyl-(1 → 4)]-*O*-(2-*O*-7*S*-hydroxydecanoic acid 7-*O*-β-D-quinovopyranosyl-(1 → 2))-α-L-rhamnopyranosyl-(1 → 2)-*O*-β-D-glucopyranosyl-(1 → 2)-*O*-β-D-fucopyranosyl-methyl 3*S*,11*S*-dihyroxytetradecanoate 3-*O*-β-D-glucopyranoside [69].

*Quamoclit pennata* also produced quamoclinic acid G-H (**242**–**243**) (Figure 8) [70]. On the other hand, the bidesmosidic quamoclinic acid G-H (**242**–**243**) has a glucose unit attached to methyl ipurolate (C-3 aglycone). Quamoclinic acid G (**242**) has a hexasaccharide core similar to that of QM10–QM13 (**237**–**240**), although the bidesmosidic moiety is unattached to the rhamnose unit compared to the aglycone. Therefore, the compound’s skeleton becomes 11-*O*-β-D-quinosyl-(1 → 3)-*O*-β-D-quinosyl-(1 → 2)-*O*-β-D-glucosyl-(1 → 3)-[*O*-β-D-quinosyl-(1 → 4)]-*O*-α-L-rhamnosyl-(1 → 2)-*O*-β-D-glucosyl-(1 → 2)-*O*-β-D-fucosyl-methyl 3*S*,11*S*-dihyroxytetradecanoate 3-*O*-β-D-glucopyranoside. Quamoclinic acid H (**243**) has a heptasaccharide core that is similar to QM2 (**231**) in the absence of the quamoclinic acid B (**2**) group in the rhamnosyl sugar. The additional glucose unit is attached to the C-3 aglycone, methyl ipurolate. The compound has a structural skeleton of 11-*O*-α-L-rhamnosyl-(1 → 3)-*O*-β-D-quinosyl-(1 → 2)-*O*-β-D-glucosyl-(1 → 3)-[*O*-β-D-quinosyl-(1 → 4)]-*O*-α-L-rhamnosyl-(1 → 2)-*O*-β-D-glucosyl-(1 → 2)-*O*-β-D-fucosyl-methyl 3*S*,11*S*-dihydroxytetradecanoate 3-*O*-β-D-glucopyranoside.

### 2.8. Oligosaccharide Ester-Type Dimer

**Figure 9:**
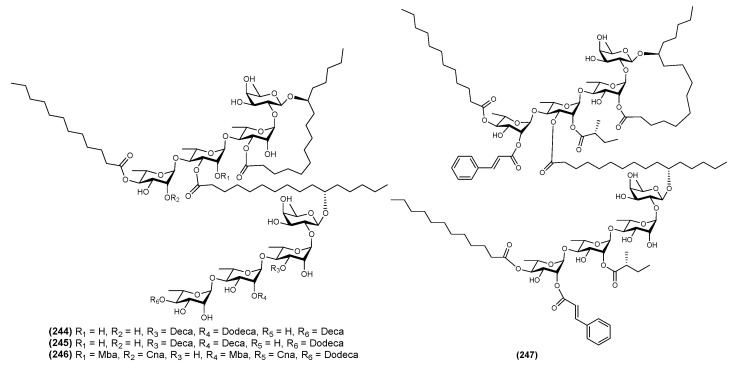

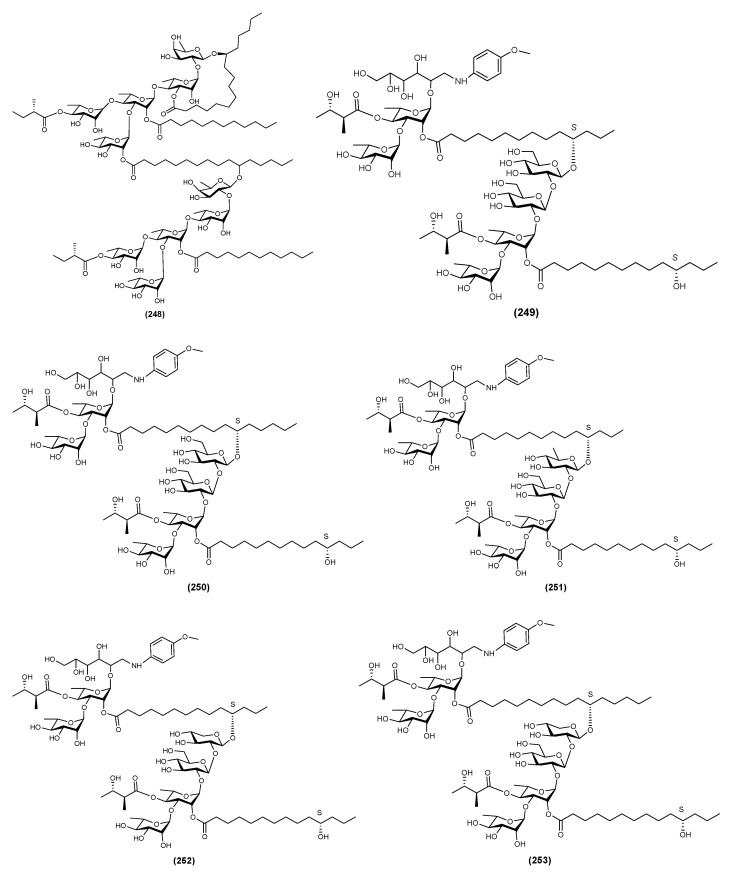

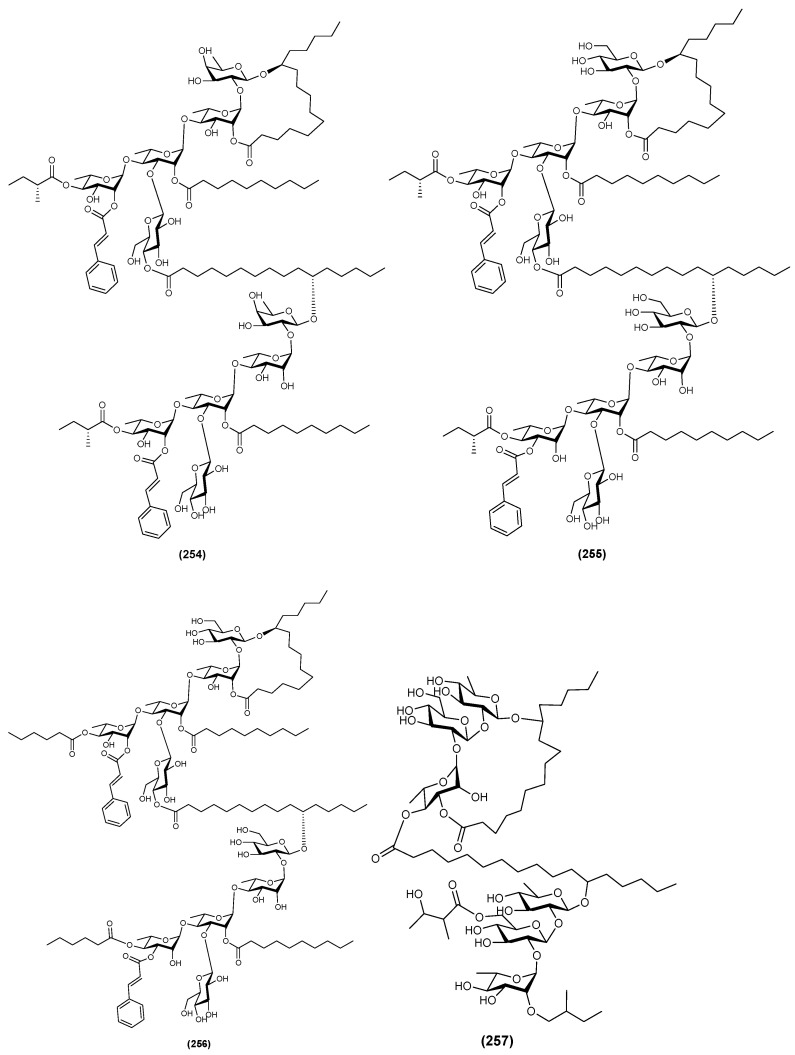

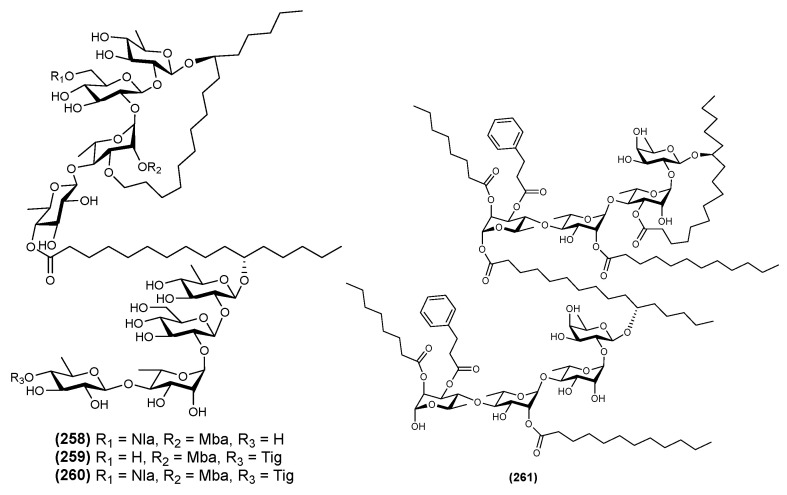
Structures of dimer-based resin glycosides **244**–**261**.

Batatins III–VI (**244**–**247**) (Figure 9) were obtained from *Ipomoea batatas* and grouped into an oligosaccharides ester-type dimer [71]. Unlike the common resin glycoside, an ester-type dimer contains two oligosaccharide units esterified by two hexadecanoic (palmitic acids) [76]. These compounds were identified to have a similar oligosaccharide core as the operculinic acid C [77], with a tetrasaccharide skeleton α-L-rhamnosyl-(1 → 4)-O-α-L-rhamnosyl-(1 → 4)-O-α-L-rhamnosyl-(1 → 2)-O-β-D-fucopyranoside for both units A and B. In unit A, the aglycone of jalapinolic acid is in the form of macrocylic lactone-1,3″, while B has an acyclic aglycone in which the C-1 ester of jalapinolic acid (unit B) is bonded to the C-3 sugar Rha′ (unit A). The difference in the structure of batatins III–VI (**244**–**247**) is due to the variety of organic acids [71]. Furthermore, other oligosaccharide ester-type dimers from *I. batatas* is batatin VII (**248**) (Figure 9) [41], which comprises units A and B with a pentasaccharide skeleton of 11-O-α-L-rhamnosyl-(1 → 3)-O-[α-L-rhamnosyl-(1 → 4)]-O-α-L-rhamnosyl-(1 → 4)-O-α-L-rhamnosyl-(1 → 2)-O-β-D-fucopyranoside, similar to those of the aquaterins [39] and pescapreins series [53]. The 11*S*-jalapinolic acid aglycone in unit A forms a macrocylic lactone with a 1,3″ ester type, which is an acyclic aglycone in unit B. Batatin VII (**248**) has a connecting point of the C-1 jalapinolic acid (unit B), bound to the C-3 of Rha″ (unit A).

Five dimeric resins were found in *Cuscuta chinensis*, namely cuses 8–12 (**249**–**253**) (Figure 9) [4]. Unlike other dimeric ester-type compounds, where the term ‘dimer’ refers to the similarity of the oligosaccharide framework between the A and B units, cuses 8–12 (**249**–**253**) are different. The A unit of the first sugar group on the anomeric carbon is poorly protected, and tautormerism occurs. Therefore, the anomeric carbon of the first sugar in unit A of cuses 8–12 (**249**–**253**) reacts with *p*-anisidine to obtain an aminoalditol derivative. Unit A of cuses 8–12 (**249**–**253**) is a trisaccharide that is similar to cuses 5–7 (**4**–**6**), while unit B is a tetrasaccharide with variations in the first sugar and the aglycone, which distinguishes the structure from cuses 8–12 (**249**–**253**). Unit B of cuses 8 (**249**) is the tetrasaccharide of 11-O-α-L-Rha-(1 → 3)-O-α-L-Rha-(1 → 2)-O-β-D-Glc-(1 → 2)-O-β-D-Glc, which is attached to the C-11 aglycone, 11*S*-convolvulic acid. Furthermore, cuses 9 (**250**) has a similar tetrasaccharide core to cuses 8 (**249**), but cuses 9 (**250**) is attached to the aglycone 11*S*-jalapinolic acid. The tetrasaccharide core of unit B of cuses 10 (**251**) is first replaced with quinovose sugar and attached to the aglycone of convolvulic acid. This turns the structural framework into 11*S*-convolvulic acid 11-O-α-L-Rha-(1 → 3)-O-α-L-Rha-(1 → 2)-O-β-D-Glc-(1 → 2)-O-β-D-Qui. Furthermore, cuses 11 (**252**) and 12 (**253**) have a xylose unit as the first sugar unit in the tetrasaccharide to give the structural skeleton of 11-O-α-L-Rha-(1 → 3)-O-α-L-Rha-(1 → 2)-O-β-D-Glc-(1 → 2)-O-β-D-Xyl. The difference between cuses 11 (**252**) and 12 (**253**) is the aglycone, in which 11*S*-convolvulic and 11*S*-jalapinolic acids, respectively.

Purgin I–III (**254**–**256**) (Figure 9) was isolated from *Ipomoea purga*, which has a structural framework of the dimeric ester of resin glycoside [6,9]. Meanwhile, Purgin I (**254**) has a similar oligosaccharide core to the pentasaccharide series of merremins [50], murasakimasarins [52], purginosides [9], and quamoclins [55], with a skeleton of 11-*O*-β-D-glucosyl-(1 → 3)-*O*-[α-L-rhamnosyl-(1 → 4)]-*O*-α-L-rhamnosyl-(1 → 4)-*O*-α-L-rhamnosyl-(1 → 2)-*O*-β-D-fucopyranoside. The aglycone 11*S*-hydroxyhexadecanoic acid in unit A is a 1,2″-macrocylic lactone, while the pentasaccharide in B is attached to the C-11 acyclic aglycone, with the C-1 aglycone, located at C-4 of the glucose unit [9]. Purgin II–III (**255**–**256**)[6] has a different oligosaccharide core from purgin I (**254**), which is 11-*O*-β-D-glucosyl-(1 → 3)-*O*-[α-L-rhamnosyl-(1 → 4)]-*O*-α-L-rhamnosyl-(1 → 4)-*O*-α-L-rhamnosyl-(1 → 2)-*O*-β-D-quinovopyranoside. The structure is similar to the pentasaccharide-type multifidin IV (**121**) [51] and operculinic acid B [77]. Furthermore, the connecting site of units A and B and their aglycones is similar to purgin I (**254**).

Three dimeric esters of resin glycosides were isolated tyrianthins C-E (**257**–**259**) (Figure 9) from *Ipomoea tyrianthina* [17]. The tetrasaccharides core in units A and B is similar to the skeleton of tyrianthinic acid VI (**48**), which is 11-*O*-β-D-quinovopyranosyl-(1 → 4)-*O*-α-L-rhamnopyranosyl-(1 → 2)-*O*-β-D-glucopyranosyl-(1 → 2)-*O*-β-D-quinovopyranoside. In unit A, the aglycone, 11*S*-hydroxyhexadecanoic acid forms a macrocylic lactone-1,3‴, while in unit B, the structure has an acyclic aglycone, with the connecting site between the C-1 aglycone and C-4 qui′ (unit A). The difference between these three compounds is the variety of organic acids.

The *Ipomoea stans* plant produced a resin glycoside, stansin A (**260**) (Figure 9), which was classfied into the dimeric ester type because it has two trisaccharide units, which are esterified with hexadecanoic acid [16]. The trisaccharide core of this compound is 11-*O*-α-L-rhamnopyranosyl-(1 → 2)-*O*-β-D-glucopyranosyl-(1 → 2)-*O*-β-D-quinovopyranoside, which is similar to the core stansoic acid A (**13**). In unit A, the 11*S*-hydroxyhexadecanoic acid aglycone forms a macrocylic lactone-1,3‴. Meanwhile, there is an acyclic aglycone in unit B, with the linking site of the C-1 aglycone (unit B) attached to the C-4 of rhamnose (unit A).

Wolcottine I (**261**) (Figure 9) is a dimeric ester-type resin glycoside obtained from *Ipomoea wolcottina* [24]. The oligosaccharide core of this compound is similar to operculinic acid C (**168**), a tetrasaccharide with the structural skeleton of 11-*O*-α-L-rhamnopyranosyl-(1 → 4)-*O*-α-L-rhamnopyranosyl-(1 → 4)-*O*-α-L-rhamnopyranosyl-(1 → 4)-*O*-β-D-fucopyranoside. The 11*S*-jalapinolic acid aglycone in unit A forms a macrocylic lactone 1,3″, while the oligosaccharides in unit B are attached to the C-11 of the acyclic aglycone, with the connecting site between the C-1 aglycone and the C-3 sugar Rha″ unit A.

This classification comprises several pieces of information with a particular species of plants responsible for producing a specific group of resin glycosides. For example, the *Operculina* and *Jalapae* species only produced hexasaccharides-typed resin glycosides, while *Porana* and *Dichondra* had trisaccharides and tetrasaccharides groups. Meanwhile, the *Ipomoea* species is the only species that produced a wide range of resin glycosides, from trisaccharides to octasaccharides, down to the dimer class. The distribution of resin glycosides in several species is shown in Table 2.

## 3. Biological Activity

Resin glycosides are not only unique in their structures but also have a wide range of biological properties. Several studies on glycosides revealed cytotoxic activities [4,11,20,29,39,42,47,51,59,75,78,79], multidrug-resistance (MDR) modulating properties [3,6,7,12,23,24,49,50,53,57,66,67,80], α-glucosidase inhibitory potential [13,36], antiviral [14], anti-metastatic [15], anti-proliferative [16,29], neuroprotective and anticonvulsant activities [10], sedative effect and gamma-aminobutyric acid (GABA) release [17,81], anti-inflammatory [18,20], antimycobacterial activity [82], the antidepressant effect [19], anticonvulsant effect/anti-seizure for epilepsy [19], the antimalarial effect [22], anti-diarrhoea [20], downregulation aquaporin 3 [15,20], and antibacterial activities [21] (Table 3). 

## 4. Isolation Techniques

The previous reviews described the use of various processes to obtain resin glycosides from plants. However, this review analysed the steps in the isolation of resin glycosides from Convolvulaceae plants. 

Most isolation techniques of resin glycosides were initiated by standard extractive procedures. The aerial part, whole part, stem, root, or leaves of the plant was macerated either by methanol [4,5,14,22,30,34,45,55,57,58,66,67,83,84], ethanol [8,29,36,37,39,40,42,43,47,48,50,53,56,64,85,86], hexane [12,74,87], a mixture of acetone:water [23], subsequent hexane/chloroform/dichloromethane [7,17,30], chloroform [6,9,24,41,88], ethyl acetate [18], subsequent hexane/dichloromethane/methanol [82], or dichloromethane [19]. The process of percolation using successive hexane, dichloromethane, and methanol [28] or chloroform/acetone [89], and also the process of soxhlet extraction using hexane/chloroform, were reported [49]. 

Mostly, methanolic and ethanolic extracts were suspended in water before the extraction. The suspension was extracted either by successive petroleum ether/dichloromethane/ethyl acetate/butanol [29,32,39,40,47,48,64], petroleum ether/ethyl acetate/butanol [56], *n*-hexane/butanol [55], 70% methanol/hexane [58], ethyl acetate/butanol [30,34,45], petroleum ether/chloroform [36], chloroform/water [8,85] petroleum ether/ethyl acetate [50], ethyl acetate/water [5,89], butanol/water [83], or hexane/chloroform/methanol [84]. Several studies took advantage of the butanol fraction to be chromatographed further to obtain resin glycosides [48,64]. Others used petroleum ether [50], ethyl acetate [34], or dichloromethane [29] fractions to obtain the resin glycosides. According to another report, the first methanolic extract obtained from maceration was directly purified by chromatographic techniques without prior partition [57]. Chromatographic techniques that were reportedly used are based on silica gel, NH_2_ gel, sephadex LH-20 [89], microporous resin D101 [4,53], Diaion HP-20, ODS, and preparative HPLC. Purifications were not straightforward, with peak-shaving and heart-cutting techniques used to recycle the preparative HPLC to obtain the resin glycosides [12]. Numerous reports describe the conversion of fractions into their acylated [64] or reductive forms [4] to improve the poor resolution of glycosidic acid. The chloroform extract was directly chromatographed [6,41] through the preparative-scale recycling of HPLC [66] to obtain resin glycosides.

Table 4 shows the representative details of isolation techniques of resin glycosides, starting from different solvents in the maceration process.

## 5. Structural Determination

The structural determination of resin glycosides has been described in preliminary reviews thoroughly [1,2]. In this review, the sequence of the general structural determination of resin glycosides will still be explained based on the reports, even though the majority are mostly similar to the ones reported in the prior reviews.

The structural determination of resin glycosides, spectroscopic, and analytical methods involved infrared, ultraviolet, nuclear magnetic resonance, mass spectrometry, specific rotation, HPLC, and GC-MS [3,4,5,6,7,12,16,17,23,24,29,32,34,36,37,40,41,42,43,47,48,49,50,55,56,58,63,64,65,66,67,68,69,90]. Most structural determination sequences involve alkaline and acidic hydrolysis, which breaks the structure into a simpler and more straightforward structures. The first alkaline hydrolysis was attempted to decay the structure into water-soluble resin glycoside and organic fatty acids, which were then analysed through the GC-MS experiment and compared with authentic samples. Furthermore, their absolute configurations were determined by optical rotation measurements, which were compared with the authentic samples. The water-soluble glycoside was then hydrolysed in acidic conditions to break the resin glycoside into glyosidic acid fractions. These sugar fractions are analysed via several methods, including GC-MS, HPLC, and specific rotations. Several studies stated that the water-soluble glycoside was partitioned with *n*-BuOH per acetylated, recycled through HPLC, and then characterized by NMR [24,67].

The absolute configuration of the sugars is determined by comparing their specific rotations and GC-MS data with the given standards. The relative configuration of the β-configuration is determined via the large coupling constants of the anomeric protons (*J* = 7.5, 8.0, 8.5 Hz). The α-configuration was determined via the chemical shifts of C-5 in the carbon NMR, while the Mosher method was used for the 11*S*-configuration [42].

An infrared experiment was used to determine the main functionalities present in the resin glycoside structure. The three functional groups contained in the infrared spectrum are hydroxyl, alkyl, and carbonyl groups [8,36,37,57]. Ultraviolet experiments showed that resin glycoside absorbs at a wavelength of 280 nm [36].

Mass spectroscopy, including ESI-MS, FAB-MS, HRAPCI-MS [57], APCI-MS [57], can be used to analyse fragments obtained during the experiments to erase overall structural determination [29,42,65]. Furthermore, the NMR experiment is one of the most powerful methods used in the structural elucidation of resin glycoside. All protons and carbons of the structure are characterized by ID and 2D-NMR. The presence of sugars can be determined by analysing the anomeric signals in the proton and carbon NMR. Meanwhile, carboxyl groups in the structure are evaluated through carbon NMR. All sugar connectivity’s in the structure, including the ester location, are determined using ^1^H-NMR, ^13^C-NMR, HSQC, COSY, and HMBC. TOCSY was also used in the structural determination [41]. However, it is not mandatory but can be added to the manuscript if the discussion is unusually long or complex. The steps in the structural elucidation of resin glycosides are described in Table 5, which presents the structural determination steps of eight representatives of the resin glycosides, including monosaccharide-, trisaccharide-, tetrasaccharide-, pentasaccharide-, hexasaccharide-, heptasaccharide-, bidesmoside-, and ester dimer-based resin glycosides.

## 6. Synthesis

The synthesis of resin glycoside is challenging because it involves stereoselective glycosylation, macrolactonization, and deprotection steps. Some of the glycosylations used in the synthesis process include Schmidt glycosylation which requires TMSOTF as an activator [11,90,91,92,93] and interrupted Pummerer reaction-mediated (IPRm) glycosylation [94,95].

Macrocyclization administered to the resin glycoside was undertaken in several ways, including Keck Macrolactonization [90,92,94], the Corey–Nicolaou macrolactonization method [93], ring closing metathesis [11], and through intramolecular glycosylation [91].

**Scheme 1:**
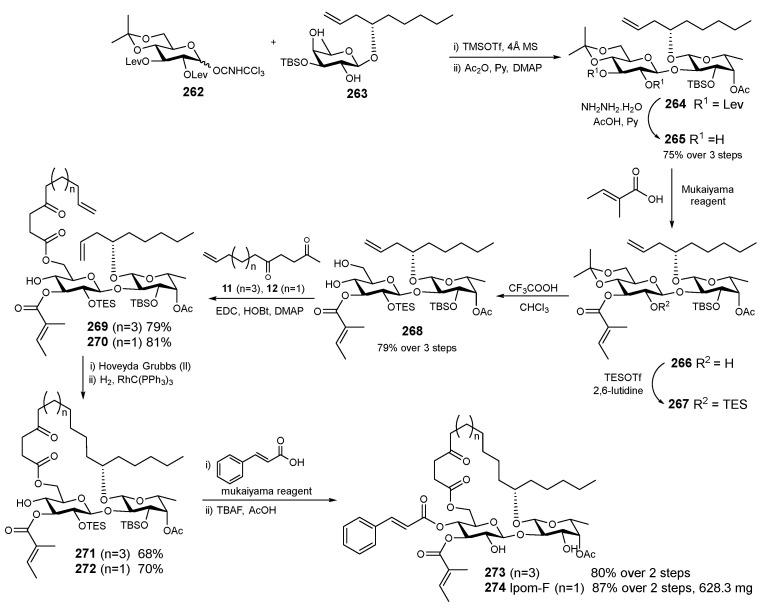
Optimized total syntheses of 22-membered ring analogue two (**273**) and ipomoeassin F (**274**).

An optimized total synthesis of the 22-membered ring of analogue (**273**) and ipomoeassin F was reported in 2020 [11]. The dissacharide core of the synthetic target was built from the glucose donor (**262**) and fucose acceptor (**263**) mediated by TMSOTf to obtain dissacharide (**264**). The deprotection of two levulinoyl groups using NH_2_NH_2_.H_2_O, AcOH in Pyridine led to a diol that was ready to be subsequently incorporated by the tiglate group (Mukaiyama method) and was sylilated using TESOTf to produce compound (**267**) in good yield. This compound was further converted to diol (**268**) using TFA in chloroform before it was conjugated with 4-oxo-10-undecenoic acid and mediated by EDC. The product (**269**)/(**270**) was then cyclized through ring-closing metathesis followed by hydrogenation to produce the cyclic product (**271**)/(**272**). The hydroxy group was then esterified using the Mukaiyama method with cinnamic acid, followed by TES and TBS deprotections to obtain the desired analogue (**273**)/ipomoeassin F (**274**), as shown in Scheme 1.

The synthetic approach in the total synthesis of tricolorin A (tetrasaccharide) was interrupted using the Pummerer reaction-mediated (IPRm) glycosylation method toward the construction of complex oligosaccharides and glycoconjugates [94]. IPRm was applied to connect two disaccharide fragments (**280** and **287**) and also to build them individually. 

**Scheme 2:**
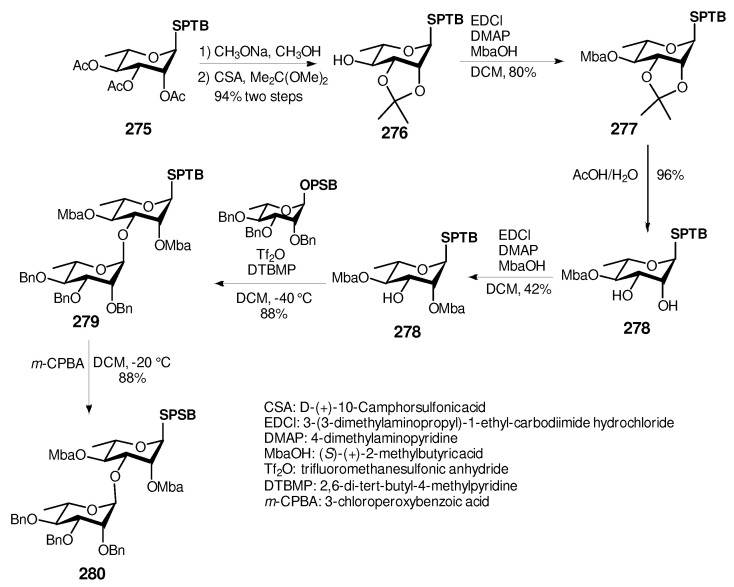
Preparation of SPSB-disaccharide (**280**).

Fragment (**280**) was constructed from the OPSB glycosyl donor and the SPTB glycosyl acceptor (**278**), as shown in Scheme 2. The acceptor (**278**) was synthesized via the subsequent deacetylation of rhamnoside (**275**) and isopropylidenation leading to (**276**) with free hydroxyl groups at position four. The obtained compound was esterified with (*S*)-(+)-2-methylbutyric acid in the presence of EDCI and DMAP to give a high yield (**277**). The following ketal deprotection and acylation of the hydroxyl group at position two resulted in the SPTB glycosyl acceptor (**278**) that was ready to be glycosylated with the OPSB donor using the IPRm method. The glycosylation produced (**279**) that was further selectively oxidized using *m*-CPBA to produce the high-yield SPSB donor (**280**).

**Scheme 3:**
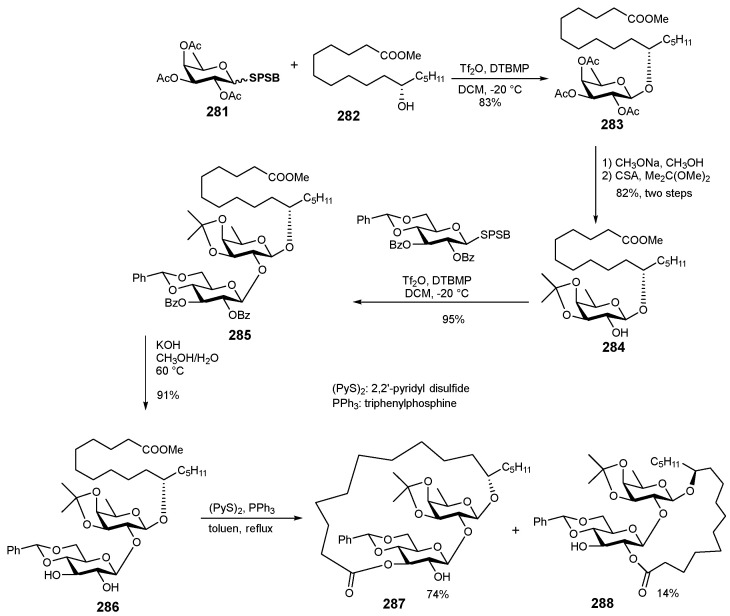
Synthesis of macrolactone disaccharide (**287**) and (**288**).

Fragment (**287**) was prepared from the SPSB donor (**281**), as shown in Scheme 3. This donor was glycosylated with methyl 11(*S*)-jalapinolate (**282**) in the presence of triflic anhydride and 2,6-di-tert-butyl-4-methylpyridine (DTBMP) to obtain compound (**283**). The deacetylation, isopropylidenization, IPRm glycosylation, and safonification resulted in the aliphatic chain-bearing disaccharide (**286**). The macrolactonization of (**286**) was found to be critical in the overall synthesis. Meanwhile, the use of 2,2′-pyridyl disulfide and triphenylphosphine gave a 19-membered ring, and 74% yield of the macrolactone (**287**).

**Scheme 4:**
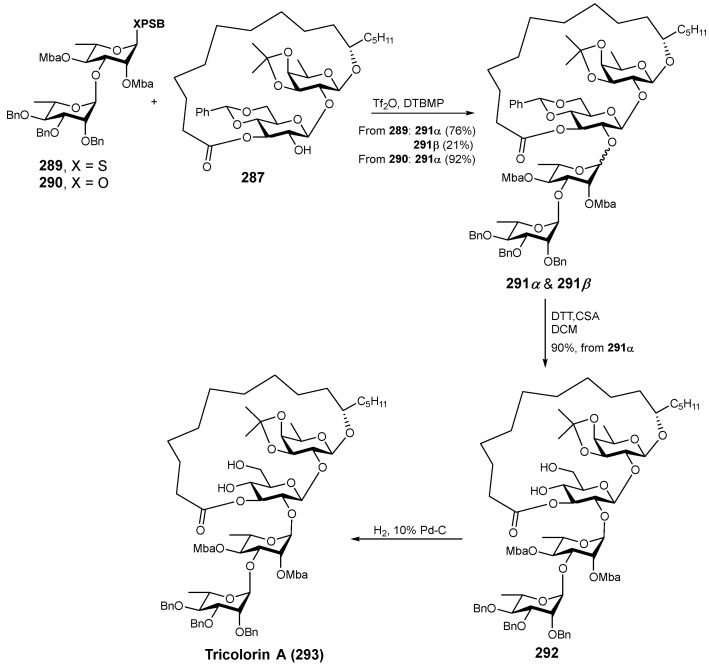
Synthesis of tricolorin A (**293**).

After obtaining fragments (**280**) and (**287**), the last step was to combine both using IPRm glycosylation, as shown in Scheme 4. However, this step only yielded moderate results and about 74% when it was run under −78 ^o^C conditions. The replacement of SPSB-based (**289**) donors with OPSB-based (**290**) increased the yield to 92%. A subsequent desopropylidenization, de-benzylidenization, and debenzylation led to the desired tricolorin A (**293**).

**Scheme 5:**
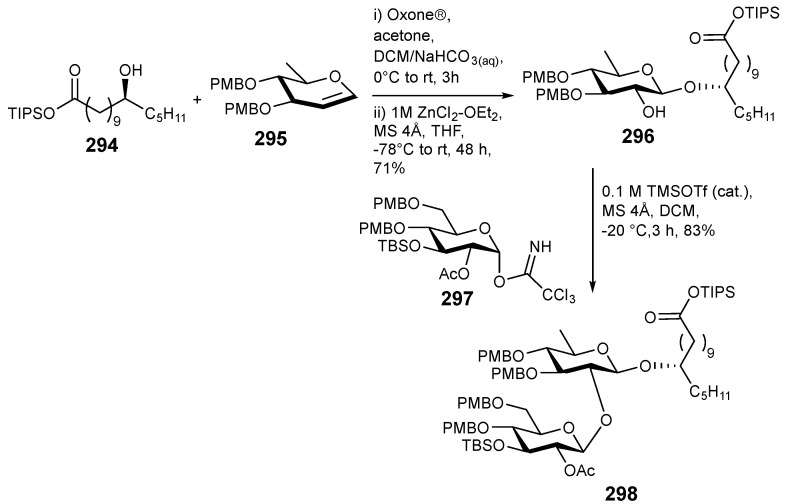
Synthesis of disaccharide (**298**).

Total synthesis of the most complex resin glycosides, which were isolated to the date-calysolin IX 27-membered ring, macrolactone was carried out using intramolecular glycosylation during the ring-closing step [91]. This approach aimed to reduce the steps in the synthesis with the retrosynthetic analysis consisting of three fragments, namely, the glycosyl donor (**301**), glycosyl acceptor (**298**), and disaccharide unit. Fragment (**298**) was synthesized from the jalapinolic ester (**294**) and was previously prepared from undecyne and hexanal, as shown in Scheme 5. The reaction of (**294**) and 6-deoxyglucal (**295**) stereoselectively conjugated using oxone, followed by zinc chloride, led to the opening of the oxirane ring to produce β-glycoside (**296**) with the free OH group at the C-2 position suitable for the next glycosylation step. The glycosylation step was carried out using trichloroacetimidate in the presence of catalytic amounts of TMSOTf to produce disaccharide (**298**) in 83% yields.

**Scheme 6:**
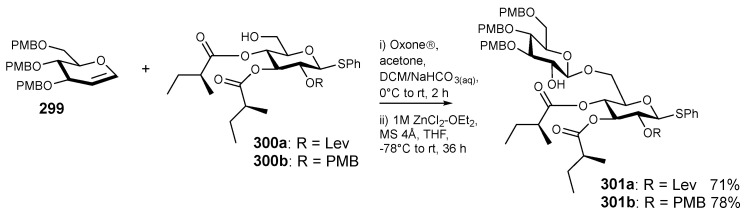
Synthesis of disaccharide (**301**).

Fragment (**301**) was synthesized using the same method as the synthesis of fragment (**298**), which involved glucal (**299**) and thioglycoside (**300**), as shown in Scheme 6.

**Scheme 7:**
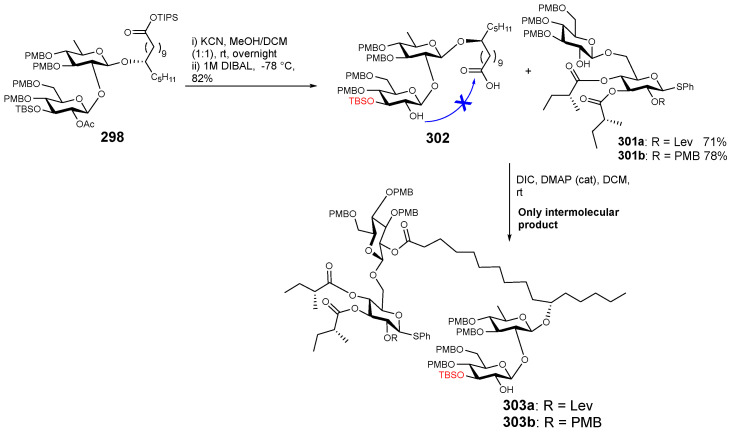
Synthesis of (**303**).

Before it was conjugated with (**301**), the protecting groups of OH and COOH were deprotected to produce intermediate (**302**), as shown in Scheme 7. The product (**302**) was conjugated with (**301**) by placing the bulky protected group of tert-butyldimethylsilyloxy at a neighbouring C-3 position to prevent intramolecular esterification between the free OH and COOH of (**302**). By applying this strategy, (**304**) could be successfully combined with fragment (**301**) using DIC/DMAP to obtain (**303**).

**Scheme 8:**
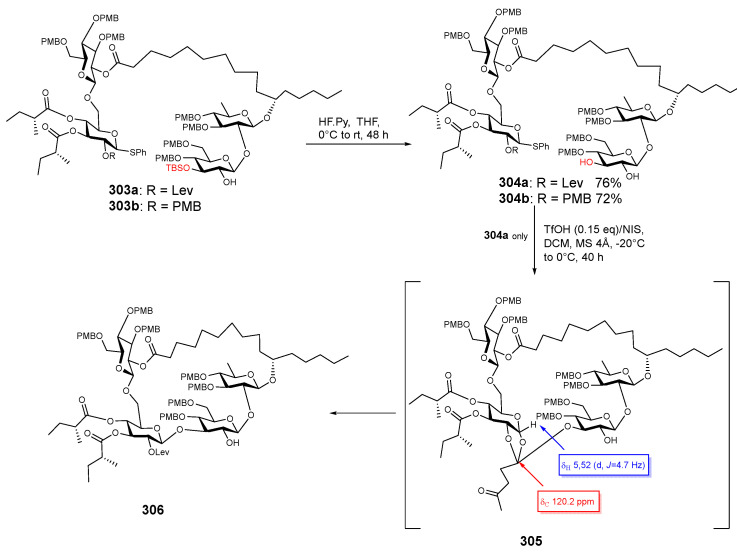
Synthesis of (**306**).

The regioselective intramolecular glycosylation of (**303**) was undertaken to produce compound (**306**), as shown in Scheme 8. The introduction of the disaccharide unit has not been reported in due course.

Two interrupted Pummerer reactions mediated with (IPRm) glycosylations were developed in the synthesis of resin glycoside [95]. This made it easy for the latent glycosides to oxidize the active glycosyl donors for the subsequent glycosylations with high efficacy. Murucoidins IV and V were synthesised through the application of the IPRm glycosylations and a sequential transient protection−glycosylation−deprotection protocol. Murucoidins IV and V were assembled in a convergent manner via a [3 + 2] IPRm glycosidic coupling and a macrolactonization.

**Scheme 9:**
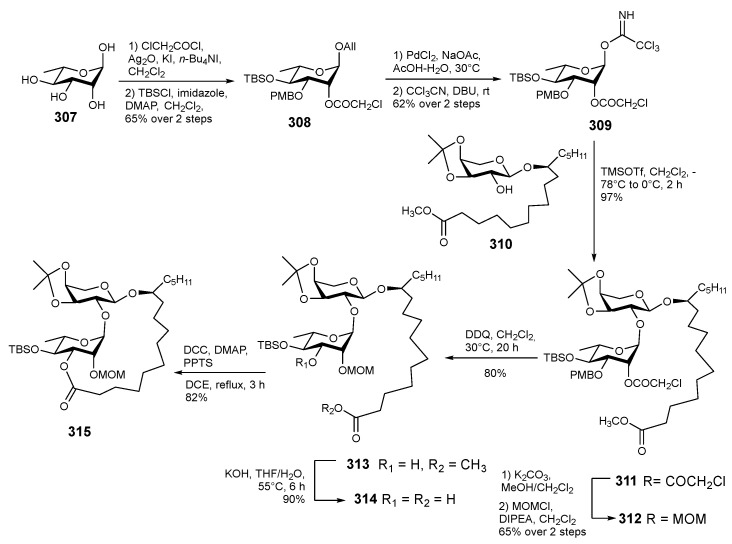
Synthesis of macrolactone (**315**).

There were efficient syntheses of the three macrocyclic lactone units by the use of a Keck macrolactonization approach [92]. The Keck macrolactonization is a carbodiimide-promoted esterification to form a macrolactone in the presence of a nitrogenous base. This reaction was applied in the preparation of macrolactones. The synthesis of macrolactone (**315**) started from allyl 3-*O*-PMB-a-L-rhamnopyranoside (**307**), as shown in Scheme 9. The overall reaction involved protection, deprotection, and glycosylation. The activation of anomeric hemiacetal was carried out using trichloroacetonitrile (CCl_3_CN) and 1,8-diazabicyclo[5.4.0] undec-7-ene (DBU) to produce trichloroacetimidate (**309**). The glycosilation steps (**309**) and (**310**) involved the Schmidt condition of a catalytic amount of trimethylsilyl trifluoromethane-ulfonate (TMSOTf) and the furnishing of disaccharide lipid (**311**) in a 97% yield. Macrolactonization took advantage of the Keck method using DCC/DMAP/PPTS.

Zhu et al. (2013) reported a total synthesis of dimeric batatin VI [90] using the convergent approach through [5 + 3] glycosidic coupling for the synthesis of (**329**) and (**335**). Pentasaccharide alcohol (**329**) was prepared by assembling three building blocks [2 + 2 + 1] (**323**–**324**–**327**), while trirhamnosyl trichloroacetimidate (**335**) was easily prepared from (**332**) and (**333**). An acyl-type functionality acting as a neighbouring participating group was incorporated at the 2-position of each donor to ensure the formation of 1,2-*trans* stereochemistry of each glycosylation. Groups of *p*-methoxybenzyl (PMB), levulinoyl (Lev), and isopropylidene groups were protected. Macrolactonization took advantage of a modified Keck method using DCC, pyridine, PPTS, 1,2-dichloroethane (DCE), and reflux at 2 hour. 

**Scheme 10:**
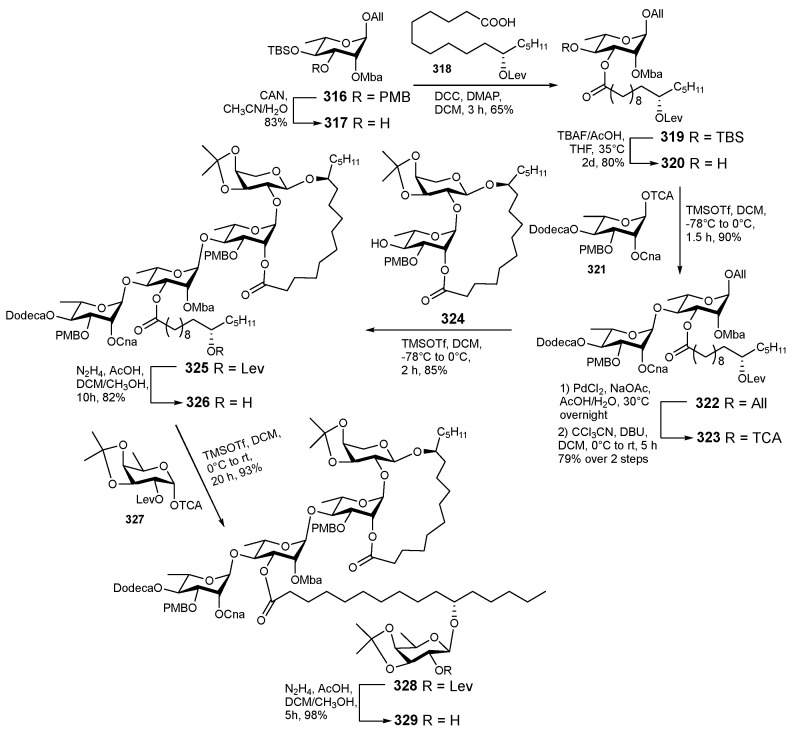
Synthesis of Pentasaccharide acceptor (**329**).

Pentasaccharide alcohol (**329**) was built from fragments of (**323**), (**324**), and (**327**), as shown in Scheme 10. Fragment (**324**) was prepared from known compound using Schmidt glycosylation (TMSOTf). Meanwhile, (**329**) was built from preparing fragment (**323**) through the subsequent PMB deprotection of (**316**), followed by the esterification of the product alcohol (**317**) with the jalapinolic acid derivative (**318**) using DCC/DMAP. TBS deprotection of the resulting (**319**), TMSOTf activation, coupled with rhamnosyl imidate to give disaccharide (**322**). The deallylation of (**322**) resulted in compound (**323**), which was ready to be coupled with fragment (**324**) prior to activation using TMSOTf. Schmidt glycosylation was trialled at first with a 45% yield of the product. An inverse glycosylation was trialled to improve the yield. Fragment (**324**) was activated using TMOSTf and reacted with (**323**) to give compound (**326**) with an 82% yield. The conjugation of product (**326**) and (**327**), followed by Lev deprotection was carried out to obtain pentasaccharide alcohol (**329**).

**Scheme 11:**
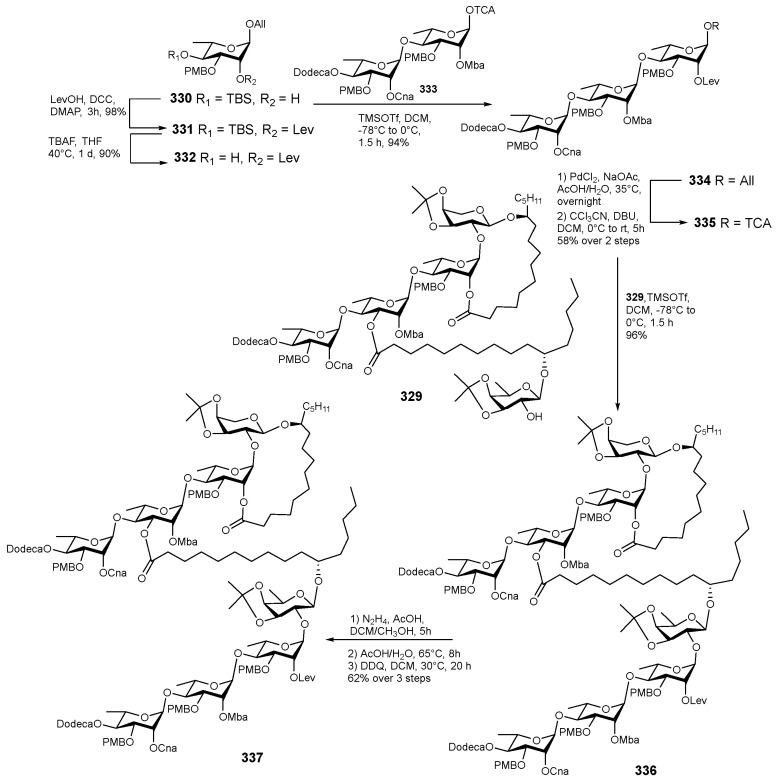
Completion of total synthesis of Batatin VI (**337**).

Intermediate (**335**) was synthesized through subsequent Lev protection and TBS deprotection before it was glycosylated with donor (**333**), as shown in Scheme 11. Finally, fragments (**335**) and (**329**) were combined through inverse *O*-glycosilation to produce a 96% yield of octasaccharide (**336**). Global deprotection of the groups in (**336**) was carried out to obtain batatin VI (**337**). 

**Scheme 12:**
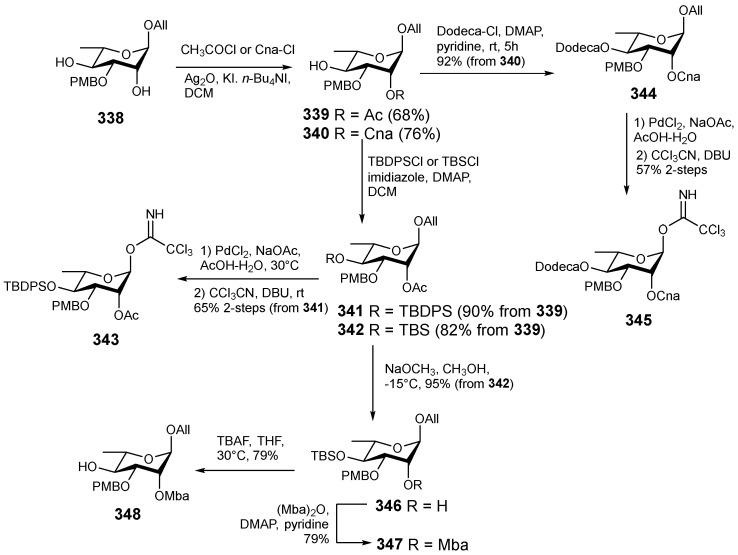
Preparation of monosaccharide building blocks.

Xie et al. (2010) reported a total synthesis of batatoside L (**362**) [93], which was carried out using a convergent strategy between fragments (**358**) and (**360**) through glycosylation. The Corey–Nicolaou macrolactonization method was involved in the formation of macrocyclic subunit (**358**). Four glycosyls trichloroacetimidate donors (**350**), (**351**), (**343**), and (**345**), and one L-rhamnosyl acceptor (**348**) were used in the preparation of fragments (**360**) and (**356**). The acyl groups serving as the necessary neighbouring participatory groups were incorporated at the 2-OH position of each donor to ensure that the desired 1,2-trans stereoselectivity was obtained for the glycosylation process. Subsequential protection followed by activation (imidation) was involved in the formation of fragment (**343**) from (**338**). Similar steps were used in the formation of (**345**). In addition, the (**348**) compounds were prepared through the aminolysis of intermediate (**345**), followed by respective esterification and desylilation, as shown in Scheme 12.

**Scheme 13:**
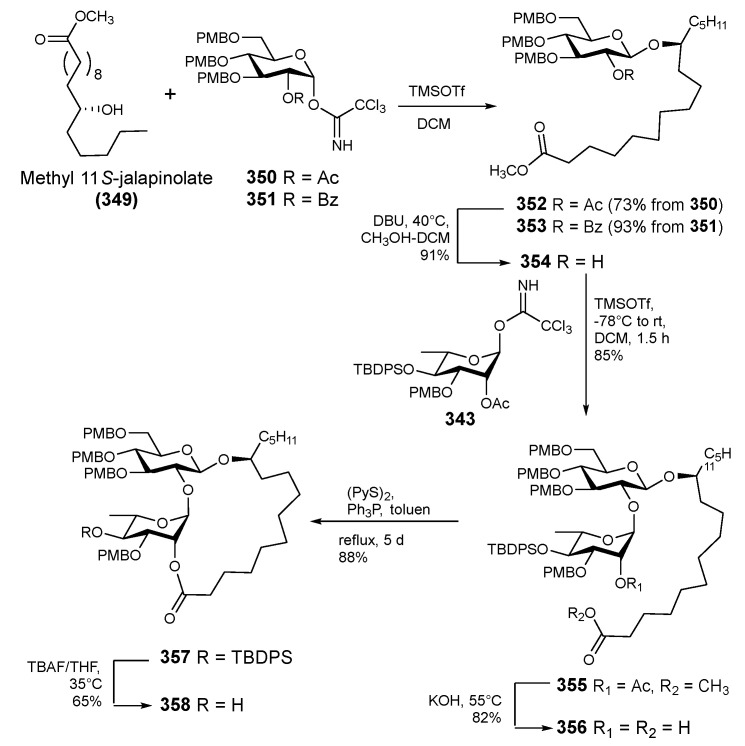
Formation of macrolactone framework.

Macrolactone (**358**) was synthesized using the Schmidt glycosylation method, as shown in Scheme 13. A chiral hydroxy ester (**349**) was glycosylated with (**350**) and (**351**) using the activating agent TMSOTf. The deacetylation process was problematic in determining the condition of DBU in methanol:dichloromethane (40 °C). This is because it resulted in the desired product that was ready to be glycosylated with donor (**343**) and the TMSOTf-activated step. The process was followed by the saponification of product (**355**) into (**338**) before the application of Corey–Nicolaou’s lactonization protocol using 2,20-pyridyl disulfide ((PyS)2) and triphenylphosphine (Ph3P). This was conducted in the highly dilute toluene (7.5 × 10^−4^ M) upon heating for 5 days to obtain compound (**357**). The product was then desylilated to obtain fragment (**358**), which was coupled to (**360**).

**Scheme 14:**
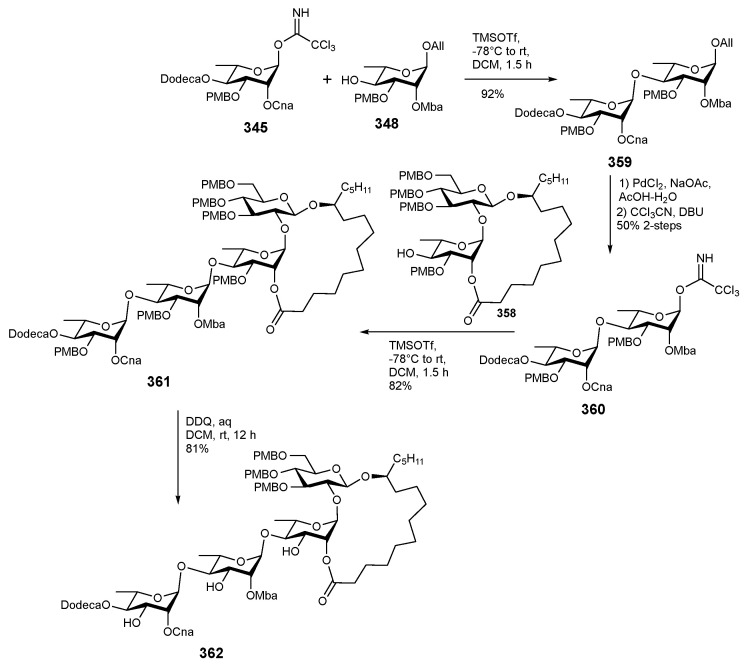
Completion of total synthesis of Batatoside L (**362**).

In preparing the exocyclic dirhamno-pyranose fragment (**360**), the imidate donor (**345**) was glycosylated with (**348**) and catalyzed by TMSOTf to produce intermediate (**359**). This compound was then concerted to imidate over two steps resulting in (**360**), which was coupled to fragment (**358**) through TMSOTf-activated glycosylation, followed by a global deprotection to give batatoside L (**362**), as shown in Scheme 14.

Synthesis of a disaccharide constituent of tricolorin A (**370**) and tricolorin F was successfully carried out through the MeOTf-promoted intermolecular glycosylation of dodecyl thioglycosyl donors [96]. Son et al. (2009) studied the intramolecular glycosyation using racemic: a mixture with MeOTf as a catalyst. The result showed that the *R* isomer went through glycosylation easier than the S isomer. Furthermore, the studies were applied to the synthesis of the disaccharide of tricolorin A and the total synthesis of tricolorin F. 

**Scheme 15:**
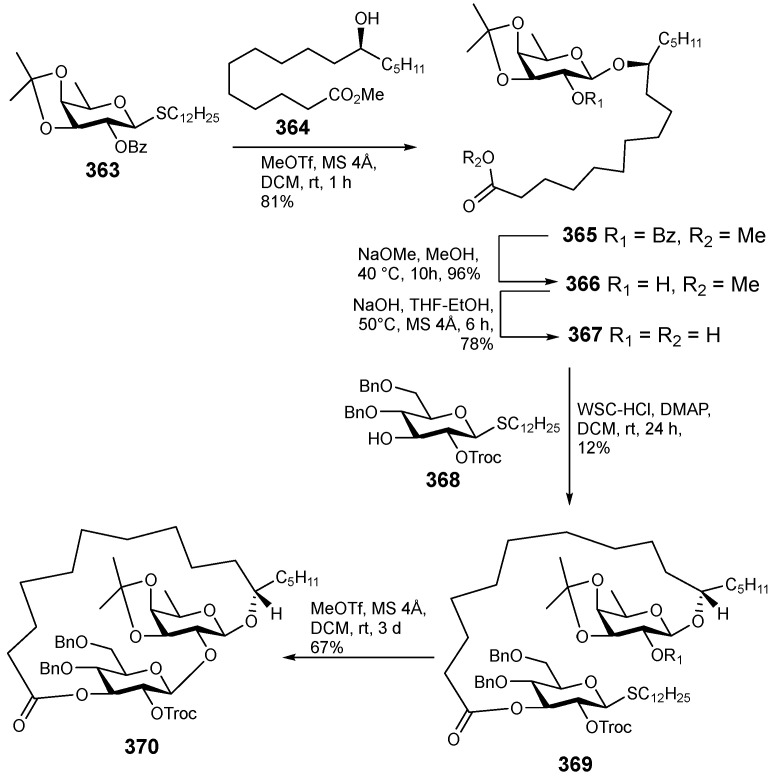
Synthesis of a disaccharide constituent of tricolorin A (**370**).

In the synthesis of disaccharide and tricolorin A (**370**), the D-fucosyl donor (**363**) was conjugated to enantiomerically pure (*S*)-methyl 11-jalapionate using MeOTf. The resulting product was then safonified and condensed to the glucosyl donor (**368**) to obtain a low-yield product (**369**). This compound was then cyclized through MeOTf-activated glycosylation to produce (**370**) as a disaccharide constituent of tricolorin A, as shown in Scheme 15.

**Scheme 16:**
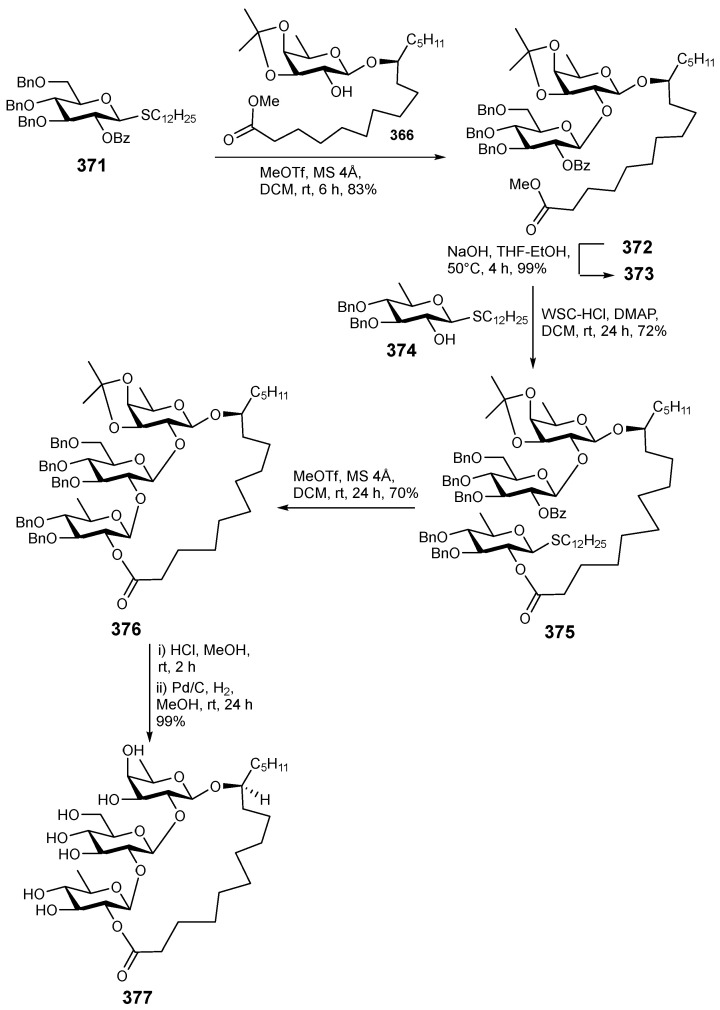
Total synthesis of tricolorin F **377**.

A total synthesis of tricolorin F (**377**) was then attempted, as shown in Scheme 16. The synthesis was initiated from the starting material donor (**371**), which was initially MeOTf-promoted and glycosylated with the fucosyl acceptor (**366**) to produce intermediate (**372**). The intermediate was then hydrolysed and conjugated with thioquinovoside (**374**) to give compound (**375**) with a 6% yield. However, the use of excess (**374**) increased the yield by 72%. The intramolecular glycosylation of compound (**375**) was undertaken using a similar MeOTf-activated glycosylate to give compound (**376**) at a 72% yield. This product was then deprotected globally to produce the desired tricolorin F (**377**).

**Scheme 17:**
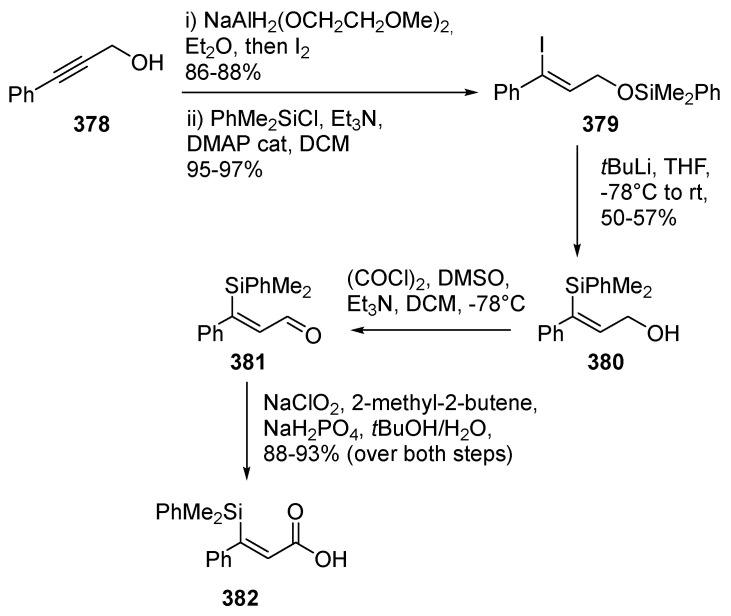
Synthesis of **382**.

In the synthesis of ipomoeassin A-F and analogues, macrocyclic rings were obtained through the ring-closing metathesis (RCM) method [97]. The C-sylillated strategy was applied successfully to synthesize the synthetic target. One of the precursors was the syllilated cinnamic acid (**382**), which was synthesized through the hydroalumination of (**378**), followed by the addition of iodine and subsequent O-silylation under standard conditions to produce product (**379**), which was subsequently converted to (**382**) through three reaction steps, as shown in Scheme 17. Lithium for the halogen exchange triggered a retro-Brook rearrangement that installed the C-silyl substituent and released the primary alcohol in (**380**), which was then oxidized to the corresponding acid (**382**) in an excellent overall yield.

**Scheme 18:**
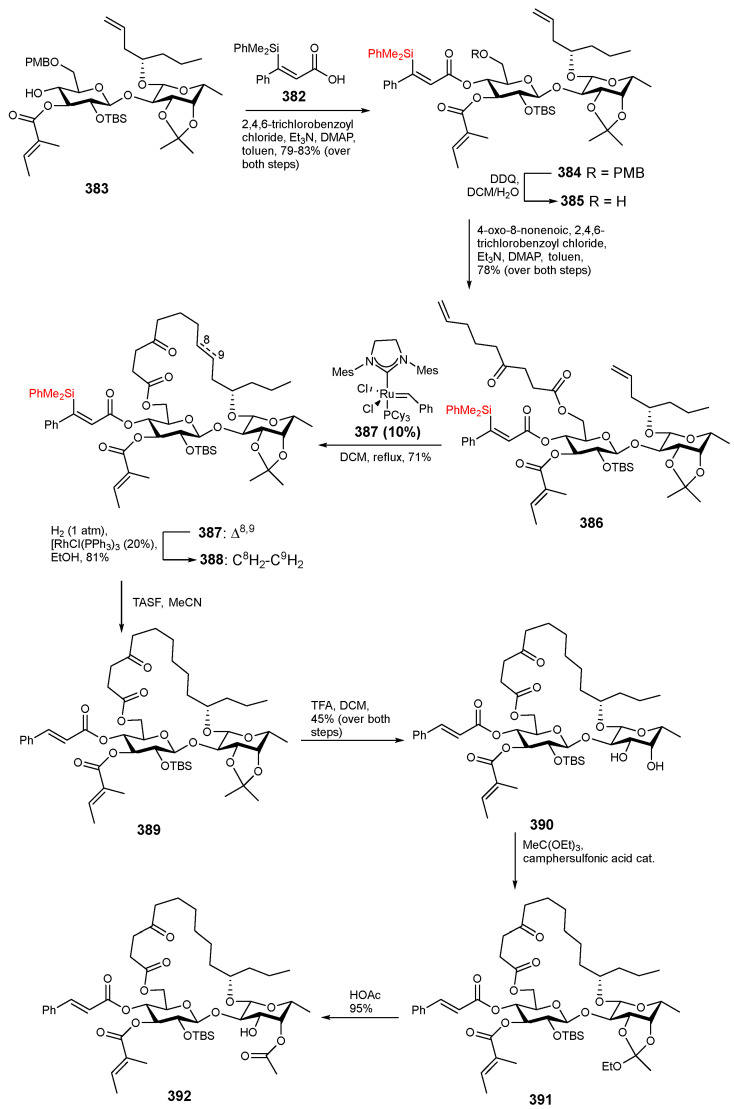
Total synthesis of ipomoeassin A (**392**) and B (**390**).

The new strategy was used to esterify the sylillated cinnamic acid (**382**) by alcohol (**383**). Furthermore, the Yamaguchi method was used to analyse product (**384**), and after the PMB deprotection of the 4-oxo-8-nonenoic acid ester segments, diene (**386**) was produced. This was followed by a macrocycle using the commercial “second-generation” ruthenium alkylidene complex (**387**). After the hydrogenation and syllil processes, OPMB deprotection, ipomoeassin A (**392**), and B (**390**) were obtained. A similar strategy was also applied to the synthesis of ipomoeassin C-F, as shown in Scheme 18.

## 7. Summary

This updated review shows that resin glycosides are a class of natural products with unique structures, comprising sugar units and the aglycone. The number of sugar unit and the variety of the aglycone, including the organic acids, make a large variation in the structure of resin glycoside. The review on the structural diversity of resin glycosides revealed that the pentasaccharides-typed resin glycosides are the dominant compounds discovered in the Convolvulaceae family, followed by the hexasaccharide type. The most complex structure of resin glycosides was found in the group of the oligomer ester. Meanwhile, the monosaccharide-typed resin glycoside made it the simplest structure of all. The unique structure of the resin glycosides is associated with the varied biological properties of the compounds, covering cytotoxic activities, MDR modulating properties, α-glucosidase inhibitory potential, antiviral, anti-metastatic, and anti-proliferative activities. It also covers the neuroprotective and anticonvulsant activities, sedative effect, GABA release, anti-inflammatory, antimycobacterial, antidepressant, anticonvulsant, antimalarial, anti-diarrhoea, downregulation aquaporin 3, and antibacterial activities. Among all of the activity, resin glycosides are known to be a good MDR modulator when they are combined with resistant drugs and have the potential to be explored more in the future. The isolation and structural characterization of resin glycosides shown in this review provide useful information. For the chemical synthesis part, the review gave information for the chemical access to the resin glycoside, and it can possibly stimulate new synthetic strategies in the future, including strategies for glycosylation to make an oligosaccharide core, cyclization, including macrolactonization, to make a macrocyclic core and also in the selection, protection, and deprotection of the hydroxyl groups during synthesis.

## Figures and Tables

**Table 1:** Classification of resin glycosides from 2010–2021.

No	Type	Sources	Refs.
*Monosaccharides*			
1	Maltifidinic acid E (**1**)	*Quamoclit × multifida*	[26]
2	Quamoclinic acid B (**2**)	*Quamoclit pennata*	[27]
3	Operculinic acid K (**3**)	*Operculina macrocarpa*	[28]
*Trisaccharides*			
1	Cuses 5 (**4**)	*Cuscuta chinensis*	[4]
2	Cuses 6 (**5**)	*Cuscuta chinensis*	[4]
3	Cuses 7 (**6**)	*Cuscuta chinensis*	[4]
4	Cuses 3 (**7**)	*Cuscuta chinensis*	[4]
5	Cuses 4 (**8**)	*Cuscuta chinensis*	[4]
6	Dichondrin C (**9**)	*Dichondra repens*	[3]
7	Poranic acid A (**10**)	*Porana duclouxii*	[5]
8	Poranic acid B (**11**)	*Porana duclouxii*	[5]
9	Poranaside A (**12**)	*Porana duclouxii*	[5]
10	Stansoic acid A (**13**)	*Ipomoea stans*	[16]
*Tetrasaccharides*			
1	Albinoside VI (**14**)	*Ipomoea alba*	[7]
2	Albinoside VII (**15**)	*Ipomoea alba*	[7]
3	Albinoside VIII (**16**)	*Ipomoea alba*	[7]
4	Albinoside IX (**17**)	*Ipomoea alba*	[7]
5	Albinosinic acid D (**18**)	*Ipomoea alba*	[7]
6	Albinosinic acid E (**19**))	*Ipomoea alba*	[7]
7	Albinosinic acid F (**20**)	*Ipomoea alba*	[7]
8	Albinosinic acid G (**21**)	*Ipomoea alba*	[7]
9	Aquaterin XI (**22**)	*Ipomoea aquatica*	[29]
10	Calonyctin E (**23**)	*Ipomoea muricata*	[23]
11	Calonyctin F (**24**)	*Ipomoea muricata*	[23]
12	Calonyctin G (**25**)	*Ipomoea muricata*	[23]
13	Calonyctin H (**26**)	*Ipomoea muricata*	[23]
14	Calonyctin I (**27**)	*Ipomoea muricata*	[23]
15	Calonyctin J (**28**)	*Ipomoea muricata*	[23]
16	Calysolin I (**29**)	*Calystegia soldanella*	[30]
17	Calysolin X (**30**)	*Calystegia soldanella*	[31]
18	Dichondrins A (**31**)	*Dichondra repens*	[3]
19	Dichondrins B (**32**)	*Dichondra repens*	[3]
20	Evolvulic acid B (**33**)	*Evolvulus alsinoides*	[32]
21	Evolvulic acid C (**34**)	*Evolvulus alsinoides*	[18]
22	Ipomotaoside A (**35**)	*Ipomoea batatas*	[18]
23	Ipomotaoside B (**36**)	*Ipomoea batatas*	[18]
24	Ipomotaoside C (**37**)	*Ipomoea batatas*	[18]
25	Ipomotaoside D (**38**)	*Ipomoea batatas*	[18]
26	Muricatic acid D (**39**)	*Ipomoea muricata*	[33]
27	Muricatin IX (**40**)	*Ipomoea muricata*	[34]
28	Muricatin X (**41**)	*Ipomoea muricata*	[33]
29	Muricatin XI (**42**)	*Ipomoea muricata*	[33]
30	Stansin 6 (**43**)	*Ipomoea stans*	[10,35]
31	Stansinic acid I (**44**)	*Ipomoea stans*	[16]
32	Tricolorin K (**45**)	*Ipomoea tricolor*	[12]
33	Tricolorin L (**46**)	*Ipomoea tricolor*	[12]
34	Tricolorin M (**48**)	*Ipomoea tricolor*	[12]
35	Tyrianthinic acids VI (**48**)	*Ipomoea tyrianthina*	[17]
36	Wolcottinoside I (**49**)	*Ipomoea wolcottiana*	[24]
*Pentasaccharides*			
1	Acutacoside A (**50**)	*Argyreia acuta*	[8]
2	Acutacoside B (**51**)	*Argyreia acuta*	[8]
3	Acutacoside C (**52**)	*Argyreia acuta*	[36]
4	Acutacoside D (**53**)	*Argyreia acuta*	[36]
5	Acutacoside E (**54**)	*Argyreia acuta*	[36]
6	Acutacoside F (**55**)	*Argyreia acuta*	[37]
7	Acutacoside G (**56**)	*Argyreia acuta*	[37]
8	Acutacoside H (**57**)	*Argyreia acuta*	[37]
9	Acutacoside I (**58**)	*Argyreia acuta*	[37]
10	Albinoside I (**59**)	*Ipomoea alba*	[38]
11	Albinoside II (**60**)	*Ipomoea alba*	[38]
12	Albinoside III (**61**)	*Ipomoea alba*	[38]
13	Albinoside IV (**62**)	*Ipomoea alba*	[7]
14	Albinoside V (**63**)	*Ipomoea alba*	[7]
15	Aquaterin I (**64**)	*Ipomoea aquatica*	[29]
16	Aquaterin II (**65**)	*Ipomoea aquatica*	[29]
17	Aquaterin III (**66**)	*Ipomoea aquatica*	[29]
18	Aquaterin IV (**67**)	*Ipomoea aquatica*	[29]
19	Aquaterin V (**68**)	*Ipomoea aquatica*	[29]
20	Aquaterin VI (**69**)	*Ipomoea aquatica*	[29]
21	Aquaterin VII (**70**)	*Ipomoea aquatica*	[29]
22	Aquaterin VIII (**71**)	*Ipomoea aquatica*	[29]
23	Aquaterin IX (**72**)	*Ipomoea aquatica*	[29]
24	Aquaterin X (**73**)	*Ipomoea aquatica*	[29]
25	Aquaterin XII (**74**)	*Ipomoea aquatica*	[39]
26	Aquaterin XIII (**75**)	*Ipomoea aquatica*	[39]
27	Aquaterin XIV (**76**)	*Ipomoea aquatica*	[39]
28	Aquaterin XV (**77**)	*Ipomoea aquatica*	[39]
29	Aquaterin XVI (**78**)	*Ipomoea aquatica*	[39]
30	Aquaterin XVII (**79**)	*Ipomoea aquatica*	[39]
31	Aquaterin XVIII (**80**)	*Ipomoea aquatica*	[39]
32	Aquaterin XIX (**81**)	*Ipomoea aquatica*	[39]
33	Arvensic acid K (**82**)	*Convolvulus arvensis*	[40]
34	Arvensic acid L (**83**)	*Convolvulus arvensis*	[40]
35	Batatinoside VII (**84**)	*Ipomoea batatas*	[41]
36	Batatinoside VIII (**85**)	*Ipomoea batatas*	[41]
37	Batatinoside IX (**86**)	*Ipomoea batatas*	[41]
38	Cairicoside A (**87**)	*Ipomoea cairica*	[42]
39	Cairicoside B (**88**)	*Ipomoea cairica*	[42]
40	Cairicoside C (**89**)	*Ipomoea cairica*	[42]
41	Cairicoside D (**90**)	*Ipomoea cairica*	[42]
42	Cairicoside E (**91**)	*Ipomoea cairica*	[42]
43	Cairicoside F (**92**)	*Ipomoea cairica*	[42]
44	Cairicoside I (**93**)	*Ipomoea cairica*	[43]
45	Cairicoside II (**94**)	*Ipomoea cairica*	[43]
46	Cairicoside III (**95**)	*Ipomoea cairica*	[43]
47	Cairicoside IV (**96**)	*Ipomoea cairica*	[43]
48	Calonyctin B (**97**)	*Ipomoea muricata*	[23]
49	Calonyctin C (**98**)	*Ipomoea muricata*	[23]
50	Calonyctin D (**99**)	*Ipomoea muricata*	[23]
51	Calyhedic acid A (**100**)	*Calystegia hederacea*	[44]
52	Calysolic acid A (**101**)	*Calystegia soldanella*	[45]
53	Calysolic acid B (**102**)	*Calystegia soldanella*	[45]
54	Calysolin II (**103**)	*Calystegia soldanella*	[30]
55	Calysolin III (**104**)	*Calystegia soldanella*	[30]
56	Calysolin V (**105**)	*Calystegia soldanella*	[46]
57	Calysolin VI (**106**)	*Calystegia soldanella*	[46]
58	Evolvulic acid A (**107**)	*Evolvulus alsinoides*	[47]
59	Evolvulin I (**108**)	*Evolvulus alsinoides*	[47]
60	Evolvulin II (**109**)	*Evolvulus alsinoides*	[47]
61	Evolvulin III (**110**)	*Evolvulus alsinoides*	[48]
62	Ipomeolide A (**111**)	*Ipomoea pes*-*caprae*	[49]
63	Ipomeolide B (**112**)	*Ipomoea pes*-*caprae*	[49]
64	Merremin A (**113**)	*Merremia hederacea*	[50]
65	Merremin B (**114**)	*Merremia hederacea*	[50]
66	Merremin C (**115**)	*Merremia hederacea*	[50]
67	Merremin D (**116**)	*Merremia hederacea*	[50]
68	Merremin E (**117**)	*Merremia hederacea*	[50]
69	Merremin F (**118**)	*Merremia hederacea*	[50]
70	Merremin G (**119**)	*Merremia hederacea*	[50]
71	Multifidin III (**120**)	*Quacmoclit × multifida*	[51]
72	Multifidin IV (**121**)	*Quacmoclit × multifida*	[51]
73	Multifidin V (**122**)	*Quacmoclit × multifida*	[51]
74	Multifidin VI (**123**)	*Quacmoclit × multifida*	[51]
75	Murasakimasarin I (**124**)	*Ipomoea batatas*	[52]
76	Murasakimasarin II (**125**)	*Ipomoea batatas*	[52]
77	Murasakimasarin III (**126**)	*Ipomoea batatas*	[52]
78	Murasakimasarin IV (**127**)	*Ipomoea batatas*	[52]
79	Murucinic acid II (**128**)	*Ipomoea murucoides*	[16]
80	Pescaprein XXI (**129**)	*Ipomoea pes*-*caprae*	[53]
81	Pescaprein XXII (**130**)	*Ipomoea pes*-*caprae*	[53]
82	Pescaprein XXIII (**131**)	*Ipomoea pes*-*caprae*	[53]
83	Pescaprein XXIV (**132**)	*Ipomoea pes*-*caprae*	[53]
84	Pescaprein XXV (**133**)	*Ipomoea pes*-*caprae*	[53]
85	Pescaprein XXVI (**134**)	*Ipomoea pes*-*caprae*	[53]
86	Pescaprein XXVII (**135**)	*Ipomoea pes*-*caprae*	[53]
87	Pescaprein XXVIII (**136**)	*Ipomoea pes*-*caprae*	[53]
88	Pescaprein XXIX (**137**)	*Ipomoea pes*-*caprae*	[53]
89	Pescaprein XXX (**138**)	*Ipomoea pes*-*caprae*	[53]
90	PM6 (**139**)	*Pharbitis nil*	[54]
91	PM7 (**140**)	*Pharbitis nil*	[54]
92	Purginoside I (**141**)	*Ipomoea purga*	[9]
93	Purginoside II (**142**)	*Ipomoea purga*	[9]
94	Purginosides III (**143**)	*Ipomoea purga*	[6]
95	Purginosides IV (**144**)	*Ipomoea purga*	[6]
96	Quamoclin V (**145**)	*Quamoclit pennata*	[55]
97	Quamoclin VI (**146**)	*Quamoclit pennata*	[55]
98	Quamoclin VII (**147**)	*Quamoclit pennata*	[55]
99	Turpethic acids A (**148**)	*Operculina turpethum*	[56]
100	Turpethic acids B (**149**)	*Operculina turpethum*	[56]
101	Turpethic acids C (**150**)	*Operculina turpethum*	[56]
102	Turpethoside A (**151**)	*Operculina turpethum*	[56]
103	Turpethoside B (**152**)	*Operculina turpethum*	[56]
104	Tyrianthinoic acid (**153**)	*Ipomoea tyrianthina*	[17]
105	Tyrianthinic acid III (**154**)	*Ipomoea tyrianthina*	[17]
106	Tyrianthinic acid IV (**155**)	*Ipomoea tyrianthina*	[17]
107	Tyrianthinic acid V (**156**)	*Ipomoea tyrianthina*	[17]
108	Wolcottinoside II (**157**)	*Ipomoea wolcottiana*	[24]
109	Wolcottinoside III (**158**)	*Ipomoea wolcottiana*	[24]
110	Wolcottinoside IV (**159**)	*Ipomoea wolcottiana*	[24]
111	A new resin glycoside (**160**)	*Ipomoea maxima*	[57]
*Hexasaccharides*			
1	Calyhedic acid B (**161**)	*Calystegia hederacea*	[44]
2	Calyhedic acids E (**162**)	*Calystegia hederacea*	[58]
3	Calyhedic acids F (**163**)	*Calystegia hederacea*	[58]
4	Calyhedin I (**164**)	*Calystegia hederacea*	[59]
5	Calyhedin II (**165**)	*Calystegia hederacea*	[59]
6	Calyhedin III (**166**)	*Calystegia hederacea*	[59]
7	Calyhedin X (**167**)	*Calystegia hederacea*	[60]
8	Calysepin I (**168**)	*Calystegia sepium*	[61]
9	Calysepin II (**169**)	*Calystegia sepium*	[61]
10	Calysepin III (**170**)	*Calystegia sepium*	[61]
11	Calysepin IV (**171**)	*Calystegia sepium*	[61]
12	Calysepin V (**172**)	*Calystegia sepium*	[61]
13	Calysepin VI (**173**)	*Calystegia sepium*	[61]
14	Calysepin VII (**174**)	*Calystegia sepium*	[61]
15	Calysolic acid C (**175**)	*Calystegia soldanella*	[45]
16	Calysolic acid D (**176**)	*Calystegia soldanella*	[45]
17	Calysolin IV (**177**)	*Calystegia soldanella*	[30]
18	Calysolin VII (**178**)	*Calystegia soldanella*	[46]
19	Calysolin VIII (**179**)	*Calystegia soldanella*	[46]
20	Calysolin IX (**180**)	*Calystegia soldanella*	[46]
21	Calysolin XI (**181**)	*Calystegia soldanella*	[31]
22	Calysolin XII (**182**)	*Calystegia soldanella*	[31]
23	Calysolin XIII (**183**)	*Calystegia soldanella*	[31]
24	Calysolin XIV (**184**)	*Calystegia soldanella*	[62]
25	Calysolin XV (**185**)	*Calystegia soldanella*	[62]
26	Calysolin XVI (**186**)	*Calystegia soldanella*	[62]
27	Calysolin XVII (**187**)	*Calystegia soldanella*	[62]
28	Calysolin XVIII (**188**)	*Calystegia soldanella*	[14]
29	Macrocarposidic acids A (**189**)	*Operculina macrocarpa*	[28]
30	Macrocarposidic acids B (**190**)	*Operculina macrocarpa*	[28]
31	Macrocarposidic acids C (**191**)	*Operculina macrocarpa*	[28]
32	Maltifidinic acid C (**192**)	*Quamoclit × multifida*	[26]
33	Maltifidinic acid D (**193**)	*Quamoclit × multifida*	[26]
35	Multifidin VII (**194**)	*Quamoclit × multifida*	[51]
36	Multifidin VIII (**195**)	*Quamoclit × multifida*	[51]
37	Operculinic acids H (**196**)	*Operculina macrocarpa*	[28]
38	Operculinic acids I (**197**)	*Operculina macrocarpa*	[28]
39	Operculinic acids J (**198**)	*Operculina macrocarpa*	[28]
40	PM1 (**199**)	*Pharbitis nil*	[54]
41	PM2 (**200**)	*Pharbitis nil*	[54]
42	PM3 (**201**)	*Pharbitis nil*	[54]
43	PM4 (**202**)	*Pharbitis nil*	[54]
44	PM5 (**203**)	*Pharbitis nil*	[54]
45	QM4 (**204**)	*Quamoclit pennata*	[63]
46	Quamoclinic acid C (**205**)	*Quamoclit pennata*	[27]
47	Quamoclinic acid D (**206**)	*Quamoclit pennata*	[27]
*Heptasaccharides*			
1	Arvensic acid A (**207**)	*Convolvulus arvensis*	[64]
2	Arvensic acid B (**208**)	*Convolvulus arvensis*	[64]
3	Arvensic acid C (**209**)	*Convolvulus arvensis*	[64]
4	Arvensic acid D (**210**)	*Convolvulus arvensis*	[64]
5	Calyhedic acid C (**211**)	*Calystegia hederacea*	[44]
6	Calyhedic acid D (**212**)	*Calystegia hederacea*	[44]
7	Calyhedin IV (**213**)	*Calystegia hederacea*	[59]
8	Calyhedin V (**214**)	*Calystegia hederacea*	[59]
9	Calyhedin VI (**215**)	*Calystegia hederacea*	[59]
10	Calyhedin VII (**216**)	*Calystegia hederacea*	[60]
11	Calyhedin VIII (**217**)	*Calystegia hederacea*	[60]
12	Calyhedin IX (**218**)	*Calystegia hederacea*	[60]
13	QM1 (**219**)	*Quamoclit pennata*	[63]
14	QM7 (**220**)	*Quamoclit pennata*	[65]
15	Quamoclinic acid E (**221**)	*Quamoclit pennata*	[27]
16	Quamoclinic acid F (**222**)	*Quamoclit pennata*	[27]
*Macrocyclic Bidesmoside*			
1	Jalapinoside B (**223**)	*Ipomoea purga*	[16]
2	Jalapinoside I (**224**)	*Ipomoea purga*	[66]
3	Jalapinoside II (**225**)	*Ipomoea purga*	[67]
4	Multifidin IX (**226**)	*Quamoclit × multifida*	[51]
5	Multifidinic acid F (**227**)	*Quamoclit × multifida*	[68]
6	Multifidinic acid G (**228**)	*Quamoclit × multifida*	[68]
7	Purgic acid C (**229**)	*Ipomoea purga*	[67]
8	Purgic acid D (**230**)	*Ipomoea purga*	[67]
9	QM2 (**231**)	*Quamoclit pennata*	[63]
10	QM3 (**232**)	*Quamoclit pennata*	[63]
11	QM5 (**233**)	*Quamoclit pennata*	[63]
12	QM6 (**234**)	*Quamoclit pennata*	[65]
13	QM8 (**235**)	*Quamoclit pennata*	[65]
14	QM9 (**236**)	*Quamoclit pennata*	[65]
15	QM10 (**237**)	*Quamoclit pennata*	[65]
16	QM11 (**238**)	*Quamoclit pennata*	[69]
17	QM12 (**239**)	*Quamoclit pennata*	[69]
18	QM13 (**240**)	*Quamoclit pennata*	[69]
19	QM14 (**241**)	*Quamoclit pennata*	[69]
20	Quamoclinic Acid G (**242**)	*Quamoclit pennata*	[70]
21	Quamoclinic Acid H (**243**)	*Quamoclit pennata*	[70]
*Oligomers Ester-type dimer*			
1	Batatin III (**244**)	*Ipomoea batatas*	[71]
2	Batatin IV (**245**)	*Ipomoea batatas*	[71]
3	Batatin V (**246**)	*Ipomoea batatas*	[71]
4	Batatin VI (**247**)	*Ipomoea batatas*	[71]
5	Batatin VII (**248**)	*Ipomoea batatas*	[41]
6	Cuses 8 (**249**)	*Cuscuta chinensis*	[4]
7	Cuses 9 (**250**)	*Cuscuta chinensis*	[4]
8	Cuses 10 (**251**)	*Cuscuta chinensis*	[4]
9	Cuses 11 (**252**)	*Cuscuta chinensis*	[4]
10	Cuses 12 (**253**)	*Cuscuta chinensis*	[4]
11	Purgin I (**254**)	*Ipomoea purga*	[9]
12	Purgin II (**255**)	*Ipomoea purga*	[6]
13	Purgin III (**256**)	*Ipomoea purga*	[6]
14	Tyrianthin C (**257**)	*Ipomoea tyrianthina*	[17]
15	Tyrianthin D (**258**)	*Ipomoea tyrianthina*	[17]
16	Tyrianthin E (**259**)	*Ipomoea tyrianthina*	[17]
17	Stansin A (**260**)	*Ipomoea stans*	[16]
18	Wolcotinne I (**261**)	*Ipomoea wolcottiana*	[24]

**Table 2:** Distribution of resin glycosides in species of Convolvulaceae.

Genus	Tri	Tetra	Penta	Hexa	Hepta	Bidesmoside	Dimer
*Cuscuta*	√						√
*Ipomoea*	√	√	√	√	√	√	√
*Porana*	√						
*Evolvulus*		√	√				
*Dichondra*		√					
*Calystegia*		√	√	√	√		
*Convolvulus*			√		√		
*Argyreia*			√				
*Merremia*			√				
*Quamoclit*			√	√	√	√	
*Operculina*				√			
*Jalapae*				√			
*Pharbitis*			√	√			

**Table 3:** Biological properties of resin glycosides.

No	Name of Compound	Activity	References
1	Ipomoeassin F and analogues (**273**–**274**)	Cytotoxic against MDA-MB 231 and Michigan Cancer Foundation-7 (MCF-7).	[11]
2	Evolvulin I (**108**)	Cytotoxic against MCF-7 cells (IC_50_ value of 3.12 μM).	[47]
3	Tricolorin A (**293**)	The MDR reversal factor of vinblastine cytotoxicity enhanced by 2164-fold in MCF/Vin cells.	[12]
4	Ipomeolides A (**111**)	Inducing apoptosis cell death in K562 cells when combined with doxorubicyn.	[49]
5	Calonyctins E (**23**), J (**28**), Muricatic acid C	The MDR reversal activity of vincristine cytotoxicity enhanced by 2.5–407.1-fold at 25 μM in KB/VCR cells (Calonyctin E was the most active).	[23]
6	Cairicosides I–IV (**93**–**96**)	α-Glucosidase inhibitory potential in vitro comparable to acarbose.	[43]
7	Cairicoside E (**91**)	Anti-metastatic effect through EMT inhibition via down-regulation of AQP5 in CRC cells.	[15]
8	Calysolin XVIII (**188**)	Anti-HSV-1 with EC_50_ value 2.3 µM and relatively high cytotoxicity (IC_50_ 8.7 μM).	[14]
9	Albinosides VII-VIII (**15**–**16**)	The MDR reversal activity of vinblastine enhanced with reversal factors (RF_MCF-7/Vin+_) of 201 and >2517-fold, respectively, in MCF/Vin cells.	[7]
10	Jalapinoside B (**223**), purgic acid D (**230**), stansoic acid A (**13**), stansin A (**43**), murucinic acid II (**128**), stansinic acid I (**44**)	A discrete effect against the proliferative rate of HCT-15 (ED50 > 24.0 µg/mL) and OVCAR (ED50 > 25.0 µg/mL (jalapinoside B, purgic acid D, stansoic acid A, murucinic acid II, and stansinic acid I). Active against the proliferation of OVCAR (ED_50_ 5.5 µg/mL), and less cytotoxic toward HCT-15 and UISO-SQC-1 (stansin A). Showing cell viability effects on C6 and RG2 cell-lines.	[16]
11	Jalapinoside II (**225**)	The MDR reversal activity of vinblastines enhanced with reversal factors (RF_MCF-7/Vin+_) of >1906 fold in MCF/Vin cells.	[67]
12	Acutacoside C-E (**52**–**54**)	A weak α-glucosidase inhibitory activity.	[3]
13	Wolcottinosides I–IV (**261**), (**157**–**159**)	The potentiation effect of the antibiotic susceptibility up to eightfold when combined with etracycline, kanamycin, or chloramphenicol.	[24]
14	Aquaterins XII-XIII (**74**–**75**), XV (77)	Cytotoxic against various cancer cell lines (IC_50_ 3.0–8.9 μM).	[39]
15	Aquaterin II (**65**)	Inducing G0/G1 arrest regulated by related proteins CDK4/6, cyclin D/E and p21. This was caused by mitochondria-mediated apoptosis with a decrease in MMP, ROS accumulation, caspase cascade activation and Bax/Bcl-2 alteration. Additionally, the suppression of the PI3K/Akt signalling pathway was observed, suggesting that it participated in the Aquaterin II–induced cell growth inhibition (antiproliferative mechanism).	[39]
16	Dichondrins A-C (**31**-**32**), (**9**)	The MDR reversal activity of vincristine enhanced with reversal factors (RF_MCF-7/Vin+_) by 1.03–1.78-fold in KB/VCR cells.	[3]
17	Jalapinoside I (**224**)	The MDR reversal activity of vincristine enhanced with reversal factors (RF_MCF-7/Vin+_) by >1906-fold in MCF-7/Vin cells.	[66]
18	Aquaterins I−IV (**64**–**67**), Aquaterin VII (**70**), Aquaterin IX-X (**72**–**73**)	Active against HepG2 cells with an IC_50_ value of 2.4 μM (aquaterin IV). Through the inhibition of the proliferation of HepG2 cells via G0/G1 arrest and apoptosis induction. Elevating Ca^2+^ in HepG2 cells (aquaterins I−IV, aquaterin VII, aquaterin IX, aquaterin X), which is involved in the regulation of the cytotoxic activities observed.	[29]
19	Merremin A (**113**), Merremin E-F (**117**–**118**), murucoidin V	The MDR reversal activity of vincristine enhanced with reversal factors by 2.3−142.5-fold in KB/VCR cells	[50]
20	Stansin 6 (**43**)	Neuroprotective and anticonvulsant activities.	[10]
21	Tyrianthins C-E (**257**–**259**)	Sedative and vasorelaxant effect.	[17]
22	Cuses 5–12 (**4**–**6**), (**249**–**253**)	Cytotoxic toward MCF-7, SMMC-7721, and MG-63 cell lines with IC_50_ values ranging from 8.72 to 59.35 mg/mL.	[4]
23	Cairicosides A–E (**87**–**91**)	Moderately cytotoxic against a small panel of human tumor cell lines with IC_50_ values in a range from 4.28 to 14.31 μM. Cairicosides A-D are more cytotoxic than cairicoside E.	[42]
24	Purgin III (256), Purginosides I–IV (**141**–**144**), Purgin I (254)	The MDR reversal activity of vinblastine enhanced with reversal factors (RF_MCF-7/Vin+_) by 1.4 to 6.5-fold in MCF-7/Vin cells.	[6]
25	Convolvulin	Sedative and vasorelaxant effect. Cytotoxic against nasopharyngeal carcinoma cell line KB (ED_50_ 2.3 μg/mL).	[81]
26	Pescapreins XXI–XXX (**129**–**138**)	Multidrug resistance modulator in the human breast cancer cell line MCF-7/ADR.	[53]
27	Ipomotaosides A (**35**)	Anti-inflammatory activity against cyclooxygenase (COX)-1 and -2.	[18]
29	Cairicosides A-B (**87**–**88**)	Strong α-glucosidase inhibitory activity with IC_50_ 25.3 ± 1.6 and 28.5 ± 3.3 μM, compared to acarbose.	[80]
30	Calysolin I–IX (**29**), **103**–**104**, **177**, **105**–**106**, **178**–**180**)	Antiviral activity against HSV-1 with IC_50_ ranging from 4.3 to 18.5 μM and EC_50_ from 1.9 to 19.2 μM.	[46]
31	Calysolin X–XIII (**30**), **181**–**183**)	Antiviral activity against HSV-1 with IC_50_ from 14.6 to 32.6 μM and EC_50_ from 2.6 to 7.5 μM.	[31]
32	Calysolin XIV–XVII (**184**–**187**)	Antiviral activity against HSV-1 with IC_50_ ranging from 7.1 to 50.4 μM and EC_50_ ranging from 3.5 to 12.8 μM.	[62]

**Table 4:** Representative details of isolation techniques of resin glycosides.

No	Plant	Part of Plant	Extraction/Separation	Refs.
1	*C* *alystegia* *soldanella*	Fresh leaves, stems and roots	Step 1: Maceration of the plant using methanol at room temperature (1 month). Step 2: Extraction of water suspension from the methanol extract with subsequent ethyl acetate and butanol. Step 3: Separation and purification of ethyl acetate fraction using subsequent silica gel column chromatography, using gradient mixtures of CHCl_3_–MeOH–H_2_O and HPLC, and using 95% and 90% MeOH as an eluent producing calysolin IX and compound 1, respectively.	[14]
2	*Ipomoea tricolor*	Seeds	Step 1: Maceration of the plant using *n*-hexane at room temperature. Step 2: Treatment of the hexane extract with chloroform and analysis of the crude by TLC and HPLC with a standard resin glycoside, tricolorin A. This step was carried out to identify the lipophilic resin glycoside mixtures. Step 3: Separation and purification of the hexane extract by preparative HPLC using the peak-shaving and heart cutting techniques to obtain ten eluates of resin glycosides.	[12]
3	*Ipomoea muricata*	Seeds	Step 1: Maceration of the plant using acetone/water (7:3 *v/v*) at room temperature (3 × 24 h). Step 2: Extraction of water suspension in the acetone/water extract with subsequent petroleum ether and ethyl acetate. Step 3: Separation and purification of the ethyl acetate fraction using silica gel column chromatography, eluting with CHCl_3_/MeOH mixtures, preparative HPLC (MeCN-H_2_O), or NH_2_ column chromatography and eluting with MeOH/H_2_O to yield resin glycosides. All compounds were further purified by Sephadex LH-20.	[23]
4	*Ipomoea alba*	Seeds	Step 1: Maceration of the plant using subsequent *n*-hexane and chloroform at room temperature. Step 2: Precipitation of chloroform-soluble fractions with methanol. Step 3: Analysis of the crude using RP-HPLC on the C-18 column for comparison with reference compounds. Step 4: Separation and purification of the crude using preparative-scale recycling HPLC, a Symmetry C18 column (Waters; 7 μm, 19 × 300 mm), isocratic elution with MeOH−CH_3_CN−H_2_O (10:7:3), and a flow rate of 8 mL/min for the first subfraction. Collection was performed by the heart-cutting technique. For the remaining fractions, isocratic elution with MeOH−CH_3_CN (7:3) and a flow rate of 8.5 mL/min was used. These procedures afforded pure resin glycosides.	[7]
5	*Ipomoea wolcottiana*	Dried flower	Step 1: Maceration of the plant using subsequent chloroform at room temperature. Step 2: Separation of chloroform extract with open silica gel column chromatography with a gradient of MeOH in CHCl_3_. Step 3: Separation of fraction-containing resin using a reversed-phase C18 (350 g) column chromatography, eluted with MeOH to eliminate pigmented residues. Step 4: Purification of resin glycoside-containing fractions using preparative recycle HPLC on a Symmetry C18 column (Waters; 7 μm, 19 x 300 mm), an isocratic elution with MeOH-CH_3_CN (9:1) at a flow rate of 8 mL/min, and a sample injection of 500 μL (sample concentration: 180 mg/mL). Eluates were collected through the technique of “heart cutting”. Each peak was independently reinjected (sample injection, 500 μL; concentration, 0.1 mg/μL), by employing the same C18 column and an isocratic elution MeOH-CH_3_CN (9:1) with a flow rate of 8.5 mL/min. These procedures afforded pure resin glycosides.	[24]
6	*Ipomoea batatas*	Aerial part	Step 1: Maceration of the plant using subsequent ethyl acetate at room temperature (six weeks). Step 2: Separation of ethyl acetate extract using silica gel column chromatography eluted with hexane-EtOAc. Step 3: Separation of fraction five using silica gel column chromatography, with hexane-EtOAc for elution. Step 4: Separation of fraction 5–2 using silica gel column chromatography eluted with hexane-EtOAc. Step 5: Purification using preparative HPLC (ODS, 88% MeOH) to obtain purified resin glycosides.	[18]
7	*Ipomoea tyrianthina*	Root	Step 1: Maceration of the plant using subsequent *n*-hexane, dichloromethane, and methanol at room temperature. Step 2: Separation of methanolic extract with C-18 silica gel column chromatography over the reversed-phase (C18) with a gradient of CH_3_OH in H_2_O for elution. Step 3: Separation of resinous fraction using Sephadex LH-20 column chromatography and preparative TLC, obtaining two chromatographic fractions. Step 4: Purification of a more polar chromatographic fraction using preparative HPLC using an Ultrasil ODS column (10 mm i.d. × 300 mm, 5 μm, Altex), eluting with a mixture of CH_3_CN-H_2_O (7:3), at a flow rate of 1 mL/min at 25 ^o^C and detection with UV at 215 nm. Chromatographic peaks were collected and reinjected until pure. This technique afforded pure resin glycosides.	[17]
8	*Operculina macrocarpa*	Root	Step 1: Percolation of the plant using subsequent *n*-hexane, dichloromethane, and methanol at room temperature. Step 2: Suspending the methanolic extract with water and sonication of the suspension. Step 3: Dissolving the water-insoluble fraction with methanol. Step 4: Liquid–liquid partion of the supernatant with butanol and evaporation of the fraction to obtain resin glycosides.	[28]

**Table 5:** Steps in the structural elucidation of representatives of resin glycosides.

No	Name of Resin Glycoside	Structural Elucidation	Refs.
1	Quamoclinic acid B (**2**) Monosaccharide *Quamoclit pennata*	Mass spectrometry (FAB-MS): showing the [M–H]^−^ ion peak at *m/z* 333 (C_16_H_29_O_7_) along with a fragment ion peak at *m/z* 187 [333–146 (6-deoxyhexose unit)]^−^ in the negative-ion mode. ^13^C-NMR: showing 16 carbons comprising one carbonyl carbon, one oxygenated methine carbon, and one terminal quinovopyranosyl unit. ^1^H-NMR: showing two equivalent methylene protons adjacent to a carbonyl group, and a primary methyl group ascribable to the 7-hydroxydecanoic acid moiety along with one β-D-quinovopyranosyl unit which has the ^4^C_1_ conformation.	[27]
2	Poranic acid A (**10**) Trisaccharides *Porana duclouxii*	Mass spectrometry (ESI-MS): exhibiting an [M–H]-ion peak at *m/z* 697 as well as a series of fragment ions at *m/z* 551 [697–146 (C_6_H_10_O_4_)]^−^, 389 [551–162 (C_6_H_10_O_5_)]^−^ and 243 [389–146 (C_6_H_10_O_4_)^−^]. ^1^H-NMR: showing three anomeric protons [δ_H_ 6.33 (br s); 5.87 (d, J 1⁄4 7.0Hz); 4.87 (d, J 1⁄4 7.5Hz)]. ^13^C-NMR: showing 32 signals, of which 18 carbons were assignable to a trisaccharide moiety and 14 carbons to the aglycone. ^1^H–^1^H COSY, HSQC, and HMBC experiments: helping to assign ^1^H and ^13^C NMR signals. Specific optical rotation-1: determining the absolute configuration of 11-hydroxytetradecanoic acid as 11S by the comparison of specific rotations of methyl ester with those obtained from Mexican jalap roots. Specific optical rotation-2: determining the absolute configurations of sugars by the direct comparison of specific optical rotation with authentic samples. GC-MS: confirming the absolute configuration through their TMSi-derivatives. HMBC: supporting the connectivities between the hydroxyl fatty acid aglycone, oligosaccharide, and among monosaccharides.	[5]
3	Calonyctins E (**23**) Tetrasaccharides *Ipomoea muricata*	Mass spectrometry (HRESIMS): showing a molecular ion peak at *m/z* 919.4528 [M + Na]^+^ (calcd. for C_42_H_72_NaO_20_, 919.4509). ^1^H-^1^H COSY: showing four spin systems, which attributed to one hexose and three 6-deoxyhexose units. HPLC analysis and optical rotations: revealing that the hexose unit belonged to D-glucose, and the three 6-deoxyhexose units belonged to two D-quinovose and one rhamnose. ^1^H NMR: suggesting the β-configurations of D-quinovose and D-glucose through the coupling constants of the anomeric protons at δ_H_ 4.75 (d, *J* = 7.8 Hz, 1H), 5.63 (d, *J* = 7.7 Hz, 1H), and 5.23 (d, *J* = 8.1 Hz, 1H). ^13^C NMR spectrum: suggesting the α-configuration of L-rhamnose through the chemical shift in C-5 of rhamnose (δ_C_ 66.9). HMBC-1: Determining the oligosaccharide core as rhamnosyl-(1->2)-[glucosyl-(1->3)]-quinosyl-(1->2)-quinosyl through the long-range HMBC correlations: H-1 of β-Qui′ (δ_H_ 5.63) to C-2 of b-Qui (δ_C_ 79.8), H-1 of α-Rha (δ_H_ 5.94) to C-2 of β-Qui′ (δ_H_ 78.7), and H-1 of β-Glc (δ_H_ 5.23) to C-3 of β-Qui′ (δ_C_ 85.1). NMR data and EIMS data: determining the aglycone to be the (11*S*)-jalapinolic acid (Jal) (after alkaline and acid hydrolyses). HMBC-2: determining the linkage between β-Qui (1/11) Jal through correlations from the H-1 of Qui (δ_H_ 4.75) to the C-11 of Jal (δ_C_ 81.8). HMBC-3: Determining the lactonization position to be H-2 of β-Glc, which indicated that the compound contained a 22-membered ring, and this was inferred by the correlation from H-2 of β-Glc (δ_H_ 5.67) to C-1 of Jal (δ_C_ 174). The compound contained an additional acetyl group (Ac) for the correlation between methyl (δ_H_ 4.75) and the carbonyl (δ_C_ 171.2). The additional HMBC correlations from δ_H_ 5.48 (H-4 of Rha) to δ_C_ 171.2 (C-1 of Ac) indicated that the OH-4 of Rha was acylated by the acetyl group.	[23]
4	Ipomeolides A (**111**) Pentasaccharides *Ipomoea pes-caprae*	Mass spectrometry (HRESIMS/MS): showing a deprotonated molecular ion at *m/z* 1249.7345 [M − H]− (calcd. 1249.7309) in the negative ion mode, consistent with the molecular formula C_63_H_110_O_24_. MALDITOF: showing a sodium adduct ion at *m/z* 1273.76. Other significant MS ions at *m/z* 1068.5688 were [M^+^ − 182 (C_12_H_22_O + H)], 1049.5551 [M^+^ − 201(C_10_H_16_O_4_ + H)], 903.4960 [M^+^ − H −183(C_12_H_23_O) − 163(C_6_H_11_O_5_)], 599.4520 [M^+^ − H − 475(C_24_H_43_O_9_) − 174(C_8_H_14_O_4_) − H], 417.5515 [M^+^ − 834(C_41_H_70_O_17_ + H)], 247.1173 [M^+^ − 1003 (C_52_H_91_O_18_)], and 205.6764 [M^+^ − H − 1044(C_55_H_96_O_18_)]. Infrared: showing hydroxy (3442 cm^−1^) and ester (1723, 1064 cm^−1^) functionalities. ^1^H-NMR-1: showing six secondary methyls at δ 1.52 (d, *J* = 6.6 Hz), 1.62 (d, *J* = 6.0 Hz), 1.66 (d, *J* = 5.4 Hz), 1.61 (d, *J* = 5.4 Hz), 1.40 (d, *J* = 6.6 Hz), and 1.21 (d, *J* = 6.6 Hz); three terminal methyls at δ 0.94 (t, *J* = 7.2 Hz), 0.88 (t, *J* = 6.5 Hz), and 0.89 (t, *J* = 7.5 Hz); five anomeric protons at δ 4.75 (d, J = 7.8 Hz), 6.17 (br s), 5.49 (br s), 5.61 (br s), 5.94 (br s), and the resonance of a long chain fatty acid. ^13^C-NMR: showing 63 carbon signals, including the signals of three carbonyls at δ 173.3, 173.4, and 176.6 ppm and five anomeric carbons at δ 104.6, 99.5, 99.1, 104.9, and 103.9 ppm. ^1^H-NMR-2: assigning the anomeric configuration of the fucosyl moiety as β, based on the large coupling constant (*J* = 7.8 Hz) of H-1′ while the proton signals arising from the rhamnose residues at δH 6.17 (br s), 5.49 (br s), 5.61 (br s), and 5.94 (br s) were consistent with an α-configuration of the anomeric protons. GC-MS: identifying the fatty acids as the n-dodecanoic acid and (2*S*)-methylbutanoic acid (a product of alkaline hydrolysis in the resin) Specific rotation: additionally identifying fatty acids through the comparison of their specific rotation value ([α]27_D_ +18 (c 0.2, CHCl_3_)) with that of an authentic sample. Mosher’s method: assigning (11*R*) configurations of (11*R*)-hydroxyhexadecanoate (the product of subsequent acid hydrolysis of glycosidic acid methyl ester). EIMS data: assigning the position of the hydroxy group at C-11 in jalapinolic acid based on crucial fragments observed at *m/z* 71, 101, and 201 in the EIMS data. GC-MS: identifying the sugars as L-rhamnopyranose and D-fucopyranose through the tetramethylsilane (TMS) derivatives. ^1^H−^1^H COSY: showing spin systems H-2−H-3−H-4 corresponding to the 2-methylbutanoyl (Mba) moiety. HMBC: determining the locations of the ester substituents, and lactonization The following key HMBC correlations were observed: H-1′′ of α-Rha′′ (δ_H_ 6.17) showed correlations with C-2′ of β-Fuc′ (δ_C_ 80.5), H-1′′′ of α-Rha′′′ (δ_H_ 5.49) with C-4′′ of α-Rha′′ (δ_C_ 80.4), H-1′′′′ of α-Rha′′′′ (δ_H_ 5.61) with C-4′′′ of α-Rha′′′ (δ_C_ 80.4), and H-1′′′′′ of α-Rha′′′′′ (δ_H_ 5.94) with C-3′′ of α-Rha′′ (δ_C_ 73.9). In addition, the position of Mba was located at C-4′′′′′ of α-Rha′′′′′, and the n-dodecanoyl moiety at C-2′′′ of α-Rha′′′ was based on the long-range correlations between H-4′′′′′ of α-Rha′′′′′ (δ_H_ 5.81) with C-1 of Mba (δ_C_ 176.6) and H-2′′′ of α-Rha′′′ (δ_H_ 5.97) with the C-1 of the dodecanoyl (δ_C_ 173.4) moiety, respectively. The position of lactonization at C-2′′ of the rhamnosyl unit was established by the correlations between H-2′′ (δ_H_ 6.02) of Rha′′ and C-1 (δ_C_ 173.3) of the jalapinolic acid moiety, respectively. NOESY: providing the diagnostic NOE correlations between the interglycosidic protons, H-1′/H-11, H-2′/H-1′′, H-3′′/H-1′′′′′, H-1′′/H-1′′′, and H-4′′′/H-1′′′′.	[49]
5	Calysolin XVIII (**188**) Hexasaccharides *Calystegia soldanella*	Mass spectrometry: finding the molecular formula to be C_72_H_120_O_35_ using high-resolution positive-ion FAB-MS. ^1^H-NMR: comparing the chemical shifts of signals arising from the sugar moieties in calysolin XVIII 1 and its methyl ester 3a, and revealing downfield shifts (Δδ = δ1–δ3a) due to the acylation of signals assignable to H-2 (Δδ = ca. 1.10) and H-4 (Δδ = 1.46) of the rhamnosyl residue (Rha), H-3 (Δδ = ca. 1.34) and H-4 (Δδ = ca. 1.56) of the second glucosyl residue (Glc′), and H-2 (Δδ = 1.46) of the fourth glucosyl residue (Glc′′′). Signals due to H2-2 of the aglycone (11*S*-jalapinolic acid) moiety (Jla) of calysolin XVIII were non-equivalent at δ 2.55 and 2.71, whereas those due to H2-2 of Jla and its methyl ester were equivalent at δ 2.32 (2H, t, *J* = 7.5 Hz) [45]. HMBC: observing the key cross peaks between the H-4 of Rha and C-1 of the 3-hydroxy-2-methylenebutyryl residue (Hma), H-3 of Glc′ and C-1 of the second 2-methylbutyryl residue (Mba′), H-4 of Glc′ and C-1 of the third 2-methylbutyryl residue (Mba′′), and H-2 of Glc′′′ and C-1 of Jla in the HMBC spectrum. Although no cross-peak was identified between the H-2 of Rha and C-1 of the first 2-methylbutyryl unit (Mba), the ester linkages of Mba, Mba′, Mba′′, Hma, and Jla were located at OH-2 of Rha, OH-3 of Glc′, OH-4 of Glc′, OH-4 of Rha, and OH-2 of Glc′′′, respectively. These linkages were supported by the fragment ion peaks observed at *m/z* 1399 [1543 (M−H)−144 (C H O)]−, 1301 684 [1399 − 98 (2-methylenebutyryl residue)]−, 1053 [1543 − 162 (hexosyl residue) − 146 (6-deox-yhexosyl residue) − 98 − 84 (2-methylbutyryl residue)]−, 579 [1053 − 162 − 144 − 84 × 2]−, 417 [579 − 162]−, and 271 [417 − 146]− in the negative-ion FAB-MS of 1 [72]. The absolute configuration of the component 2-methylbutyric acid of the crude resin glycoside fraction of this plant was previously determined to be *S* [45].	[14]
6	Arvensic acid A-D (**207**–**210**) Heptasaccharides *Convolvulus arvensis*	Mass spectrometry: determining molecular formula to be C_57_H_100_O_35_, C_58_H_102_O_35_, C_57_H_100_O_36_, and C_58_H_102_O_36_, respectively, according to the HRESIMS data of [M − H]− at *m/z* 1343.5999, 1357.6155, 1359.5959, 1373.6099, respectively. NMR: The ^1^H and ^13^C NMR spectra of compounds 1–4 were similar with each other, especially in the signal region of sugar moieties (δ_H_ 3.5–6.5, δ_C_ 60–110). ^1^H NMR: seven anomeric proton signals at δ 4.95 (1H, d, *J* = 7.5 Hz), 5.80 (1H, d, *J* = 7.5 Hz), 6.35 (1H, br s), 5.39 (1H, d, *J* = 7.5 Hz), 4.92 (1H, d, *J* = 7.5 Hz), 5.46 (1H, br s), and 4.91 (1H, d, *J* = 7.5 Hz), and three secondary methyl resonances at δ 1.78 (3H, d, *J* = 6.0 Hz), 1.48 (3H, d, *J* = 6.5 Hz), and 1.66 (3H, d, *J* = 6.0 Hz), were assignable to the CH_3_–6 of 6-deoxyhexoses. ^13^C NMR: seven anomeric carbon signals in the range δ 102.2–106.5. 2D NMR spectra (HSQC, HMBC, 1He1H COSY and TOCSY): allowing the assignment of the heptasaccharide skeleton, which was comprised of one D-fucose, two L-rhamnose, and four D-glucose units. ^1^H NMR: determining the β-configurations of the fucose and glucose units by the large coupling constants of the anomeric protons. ^13^C NMR: determining the α-configurations for L-rhamnose units by the chemical shifts of C-5 of rhamnose. HMBC: showing the connnectivities between the aglycones (Ag) and sugar chains, and among the monosaccharides in 1–4, which were established according to their HMBC spectra in which, also using one as an example, long range correlations were observed from δ_H_ 4.95 (H-1, Glc) to δ_C_ 81.6 (C-12, Ag), from δ_H_ 5.80 (H-1, Glc′) to δ_C_ 79.6 (C-2, Glc), from δ_H_ 6.35 (H-1, Rha) to δ_C_ 78.0 (C-2, Glc′), from δH 5.39 (H-1, Glc″) to δ_C_ 84.1 (C-3, Rha), from δ_H_ 4.92 (H-1, Fuc) to δ_C_ 81.9 (C-4, Glc ″), from δ_H_ 5.46 (H-1, Rha′) to δ_C_ 68.7 (C-6, Glc), and from δ_H_ 4.91 (H-1, Glc‴) to δ_C_ 90.3 (C-3, Glc′).	[64]
7	Jalapinoside I (**224**) Bidesmoside *Ipomoea purga*	Mass spectrometry (HRESIMS and MALDI-TOFMS): identifying quasi-molecular ions at *m/z* 1689.8448 [M + H]+ (C_80_H_137_O_37_). LC-ESIMS/MS showing a quasi-molecular ion of low abundance at *m/z* 1313 [M − H]−, corresponding to the molecular formula C_56_ H_97_O_34_, and an intense ion at *m/z* 1167 [M − H − 146]−, corresponding to the elimination of one methylpentose unit which, in turn, produced the fragmentation pattern of a hexasaccharide with the same glycosidation sequence as purgic acid A but with a difference of 16 amu based on a negative ion FAB with diagnostic ions at *m/z* 1021 [1167 − 146]−, 859 [1021 − 162]−, 713 [859 − 146]−, 567 [713 − 146]−, and 405 [567 − 162]−, indicating the presence of ipurolic acid as the aglycone. ^1^H and ^13^C NMR: the data were compared with purgic acids A and B. The main differences were in the esterifying residues and macrolactonization, as well as the presence of ipurolic acid as the aglycone, containing an additional quinovose which was glycosidically linked at C-3 to constitute a macrocyclic resin glycoside bisdesmoside. COSY and TOCSY-1: assigning chemical shift values after identifying and differentiating each of the ^1^H NMR signals. ^1^H−^13^C HSQC: sssigning the ^13^C NMR signals NMR: confirming seven anomeric signals at δ_H_ 4.90 (1H, d, *J* = 7.6 Hz; δ_C_ 102.9, Qui-1); 5.86 (1H, d, *J* = 7.8 Hz; δ_C_ 101.6, Glc-1); 6.47 (1H, d, *J* = 1.3 Hz; δ_C_ 96.8, Rha-1); 6.42 (1H, d, *J* = 7.8 Hz; δ_C_ 101.6, Glc′-1); 5.15 (1H, d, *J* = 7.8 Hz; δ_C_ 106.0, Qui′-1); 5.78 (1H, d, *J* = 7.6 Hz; δ_C_ 101.8, Fuc-1); and 4.82 (1H, d, *J* = 7.8 Hz; δ_C_ 103.0, Qui′′-1). COSY and TOCSY-2: distinguishing seven separate spin systems for sugar skeletons. HMBC: establishing the glycosylation sequence and the positions of the esterification, lactonization, and ester-type linkage. The following key correlations were observed: connectivities between H-1 (δ_H_ 4.90, Qui-1) and C-11 (δ_C_ 81.2, Ipur-11); H-1 (δ_H_ 4.82, Qui′′-1) and C-3 (δ_C_ 68.6, Ipur-3); H-1 (δ_H_ 5.86, Glc-1) and C-2 (δ_C_ 80.4, Qui-2); H-2 (δ_H_ 4.28−4.26, Glc-2) and C-1 (δ_C_ 96.8, Rha-1); H-1 δ_H_ 6.42, Glc′-1) and C-3 (δ_C_ 74.0, Rha-3); H-1 (δ_H_ 5.15, Qui′-1) and C-2 (δ_C_ 86.3, Glc′-2); and H-4 (δ_H_ 4.61, Rha-4) and C-1 (δ_C_ 101.8, Fuc-1). The locations of the acyl residues were established by the following HMBC correlations: H2-6 (δ_H_ 5.69 and 5.66, Glc-6) and the carbonyl of an acetate group (δ_C_ 168.5); H-2 (δ_H_ 5.91, Rha-2) and C-1 of a 2-methylbutyrate residue (δ_C_ 176.6); H-3 (δ_H_ 5.35, Qui′-3) and C-1 of a second methylbutyrate moiety (δ_C_ 176.2); and H-3 (δ_H_ 5.99, Fuc-3) with the carbonyl of a dodecanoate group (δ_C_ 173.1).	[66]
8	Batatin VII (**248**) Oligomer ester type dimer *Ipomoea batatas*	Mass spectrometry (ESI and FAB in the negative ion detection): allowing for the detection of the quasi-molecular ion [M − H]^−^, indicating a composistion of C_126_H_219_O_48_. A fragment [M/2 − H]− was observed in both C_126_H_219_O_48_ ionization techniques, which indicated the rupture of the ester type bond and represented the high-mass fragment ions for the two monomeric units (*m/z* 1249 [A − H]^−^ and *m/z* 1251 [B − H]) as previously reported for the batatin series. This technique provided an easily detectable and intense quasi-molecular ion [M − H]− as well as all of the characteristic ions resulting from the glycosidic cleavage. The FAB/MS of batatinosides VII showed a peak at *m/z* 1181 [M − H]−, indicating a molecular formula of C_58_H_102_O_24._ MALDI/MS: confirming the molecular formula. The fragmentation pattern was determined with the peaks observed at *m/z* 271, 417, 545, 837, and 999 that are common to resin glycosides containing operculinic acid A as their glycosidic acid. The fragment at *m/z* 999 [1181 − C_12_H_22_O] wasindicated by the loss of glucose as identified by the fragment at *m/z* 837 [999 − C_6_H_10_O_5_]. NMR: determining the presence of two units of simonic acid B, through a comparison of its spectroscopic constants with those previously reported for batatins I and II. This dimer illustrates the four-step approach for the identification of carbohydrate structural elements in the oligosacharaide core. Estimations of the sugar units were conducted through the anomeric protons around 4.8−6.4 ppm, which were used as “structural reporter groups” for the dimeric structure. COSY and TOCSY: identifying each constitutive monosaccharide through the assignment of chemical shift values, after identifying and differentiating the ^1^H NMR signals. HSQC: providing the assignments for the ^13^C NMR data. HMBC: observing the glycosylation sequence and the positions of esterification and lactonization. The carbonyl resonances at δ_C_ 175.2 were assigned to the unit A lactone functionality due to its ^2^JCH coupling with the C-2 diastereotopic methylene protons centered at δ_H_ 2.26−2.30. The macrolactonization site at C-3 of the first rhamnose unit was established by the observed ^3^J correlation between this CH carbonyl carbon and the H-3 of rhamnose (δ_H_ 5.63). The ester-type linkage established by the acyclic unit B at the macrocyclic unit A was identified by ^3^JCH correlation between the carbonyl group for the ester (δ_C_ 174.9; unit B) and H-2 of the terminal rhamnose (Rha′′′, δ_H_ 5.77) on unit A. The dodecanoyl residue on both units A and B (δ_C_ 173.4) was located at C-2 of the third saccharide unit (Rha′, δH 4−99). The methylbutanoyl residue (δ_C_ 166.9, unit A; δ_C_ 166.4, unit B) was located as the acylating group at positions C-4 (δ_H_ 5.86, unit A; and δ_H_ 5.93, unit B) of the terminal rhamnose unit (Rha′′).	[41]

## References

[1] Pereda-Miranda R., Rosas-Ramírez D., Castañeda-Gómez J. (2010). Resin Glycosides from the Morning Glory Family. Fortschritte der Chemie Organischer Naturstoffe/Progress in the Chemistry of Organic Natural Products.

[2] Ono M. (2017). Resin Glycosides from Convolvulaceae Plants. J. Nat. Med..

[3] Song W.B., Wang W.Q., Zhang S.W., Xuan L.J. (2015). Multidrug Resistance-Reversal Effects of Resin Glycosides from *Dichondra repens*. Bioorg. Med. Chem. Lett..

[4] Fan B.Y., Luo J.G., Gu Y.C., Kong L.Y. (2014). Unusual Ether-Type Resin Glycoside Dimers from the Seeds of *Cuscuta chinensis*. Tetrahedron.

[5] Ding W.B., Zhang D.G., Liu C.J., Li G.H., Li Y.Z. (2014). Resin Glycosides from *Porana duclouxii*. J. Asian Nat. Prod. Res..

[6] Castañeda-Gómez J., Figueroa-González G., Jacobo N., Pereda-Miranda R. (2013). Purgin II, a Resin Glycoside Ester-Type Dimer and Inhibitor of Multidrug Efflux Pumps from *Ipomoea purga*. J. Nat. Prod..

[7] Cruz-Morales S., Castañeda-Gómez J., Rosas-Ramírez D., Fragoso-Serrano M., Figueroa-González G., Lorence A., Pereda-Miranda R. (2016). Resin Glycosides from *Ipomoea alba* Seeds as Potential Chemosensitizers in Breast Carcinoma Cells. J. Nat. Prod..

[8] Yin Y.Q., Pan J.T., Yu B.W., Cui H.H., Yan Y.S., Chen Y.F. (2016). Two Pentasaccharide Resin Glycosides from *Argyreia acuta*. Nat. Prod. Res..

[9] Castañeda-Gómez J., Pereda-Miranda R. (2011). Resin Glycosides from the Herbal Drug Jalap (*Ipomoea purga*). J. Nat. Prod..

[10] León-Rivera I., Villeda-Hernández J., Campos-Peña V., Aguirre-Moreno A., Estrada-Soto S., Navarrete-Vázquez G., Rios M.Y., Aguilar-Guadarrama B., Castillo-España P., Rivera-Leyva J.C. (2014). Evaluation of the Neuroprotective Activity of Stansin 6, a Resin Glycoside from *Ipomoea stans*. Bioorg. Med. Chem. Lett..

[11] Zong G., Hu Z., Duah K.B., Andrews L.E., Zhou J., O’Keefe S., Whisenhunt L., Shim J.S., Du Y., High S. (2020). Ring Expansion Leads to a More Potent Analogue of Ipomoeassin F. J. Org. Chem..

[12] Castañeda-Gómez J., Lavias-Hernández P., Fragoso-Serrano M., Lorence A., Pereda-Miranda R. (2019). Acylsugar Diversity in the Resin Glycosides from *Ipomoea tricolor* Seeds as Chemosensitizers in Breast Cancer Cells. Phytochem. Lett..

[13] Rosas-Ramírez D., Escandón-Rivera S., Pereda-Miranda R. (2018). Morning Glory Resin Glycosides as α-Glucosidase Inhibitors: In Vitro and in Silico Analysis. Phytochemistry.

[14] Ono M., Kanemaru Y., Yasuda S., Okawa M., Kinjo J., Miyashita H., Yokomizo K., Yoshimitsu H., Nohara T. (2017). A New Resin Glycoside from *Calystegia soldanella* and Its Antiviral Activity towards Herpes. Nat. Prod. Res..

[15] Chen C., Ma T., Zhang C., Zhang H., Bai L., Kong L., Luo J. (2017). Down-Regulation of Aquaporin 5-Mediated Epithelial-Mesenchymal Transition and Anti-Metastatic Effect by Natural Product Cairicoside E in Colorectal Cancer. Mol. Carcinog..

[16] León-Rivera I., del Río-Portilla F., Enríquez R.G., Rangel-López E., Villeda J., Rios M.Y., Navarrete-Vázquez G., Hurtado-Días I., Guzmán-Valdivieso U., Núñez-Urquiza V. (2017). Hepta-, Hexa-, Penta-, Tetra-, and Trisaccharide Resin Glycosides from Three Species of *Ipomoea* and Their Antiproliferative Activity on Two Glioma Cell Lines. Magn. Reson. Chem..

[17] León-Rivera I., Castro J.M., Mirón-López G., del Río-Portilla F., Enríquez R.G., Reynolds W.F., Estrada-Soto S., Rendón-Vallejo P., del Carmen Gutiérrez M., Herrera-Ruiz M. (2014). Resin Glycosides from *Ipomoea tyrianthina* and Their Sedative and Vasorelaxant Effects. J. Nat. Med..

[18] Yoshikawa K., Yagi C., Hama H., Tanaka M., Arihara S., Hashimoto T. (2010). Ipomotaosides A-D, Resin Glycosides from the Aerial Parts of *Ipomoea batatas* and Their Inhibitory Activity against COX-1 and COX-2. J. Nat. Prod..

[19] Mirón-López G., Herrera-Ruiz M., Estrada-Soto S., Aguirre-Crespo F., Vázquez-Navarrete L., León-Rivera I. (2007). Resin Glycosides from the Roots of *Ipomoea tyrianthina* and Their Biological Activity. J. Nat. Prod..

[20] Zhu D., Chen C., Bai L., Kong L., Luo J. (2019). Downregulation of Aquaporin 3 Mediated the Laxative Effect in the Rat Colon by a Purified Resin Glycoside Fraction from Pharbitis Semen. Evid.-Based Complement. Altern. Med..

[21] Nguyen H.T., Yu N.H., Park A.R., Park H.W., Kim I.S., Kim J.C. (2017). Antibacterial Activity of Pharbitin, Isolated from the Seeds of Pharbitis Nil, against Various Plant Pathogenic Bacteria. J. Microbiol. Biotechnol..

[22] Tasdemir D., Brun R., Franzblau S.G., Sezgin Y., Çalis I. (2008). Evaluation of Antiprotozoal and Antimycobacterial Activities of the Resin Glycosides and the Other Metabolites of *Scrophularia cryptophila*. Phytomedicine.

[23] Wang W.Q., Liu S.S., Song W.B., Li J., Xuan L.J. (2018). Resin Glycosides from the Seeds of *Ipomoea muricata* and Their Multidrug Resistance Reversal Activities. Phytochemistry.

[24] Corona-Castañeda B., Rosas-Ramírez D., Castañeda-Gómez J., Aparicio-Cuevas M.A., Fragoso-Serrano M., Figueroa-González G., Pereda-Miranda R. (2016). Resin Glycosides from *Ipomoea wolcottiana* as Modulators of the Multidrug Resistance Phenotype in Vitro. Phytochemistry.

[25] Fan B.Y., Jiang X., Li Y.X., Wang W.L., Yang M., Li J.L., Wang A.D., Chen G.T. (2022). Chemistry and Biological Activity of Resin Glycosides from Convolvulaceae Species. Med. Res. Rev..

[26] Ono M., Kishida M., Ikegami Y., Takaki Y., Okawa M., Kinjo J., Yoshimitsu H., Nohara T., Miyahara K. (2011). Components of Convolvulin from *Quamoclit × multifida*. J. Nat. Med..

[27] Ono M., Takagi-Taki Y., Honda-Yamada F., Noda N., Miyahara K. (2010). Components of Ether-Insoluble Resin Glycoside (Convolvulin) from Seeds of *Quamoclit pennata*. Regul. Artic. Chem. Pharm. Bull..

[28] Lira-Ricárdez J., Pereda-Miranda R., Castañeda-Gómez J., Fragoso-Serrano M., Simas R.C., Leitão S.G. (2019). Resin Glycosides from the Roots of *Operculina macrocarpa* (Brazilian Jalap) with Purgative Activity. J. Nat. Prod..

[29] Fan B.Y., Gu Y.C., He Y., Li Z.R., Luo J.G., Kong L.Y. (2014). Cytotoxic Resin Glycosides from *Ipomoea aquatica* and Their Effects on Intracellular Ca^2+^ Concentrations. J. Nat. Prod..

[30] Takigawa A., Muto H., Kabata K., Okawa M., Kinjo J., Yoshimitsu H., Nohara T., Ono M. (2011). Calysolins I-IV, Resin Glycosides from *Calystegia soldanella*. J. Nat. Prod..

[31] Ono M., Kawakami G., Takigawa A., Kabata K., Okawa M., Kinjo J., Yokomizo K., Yoshimitsu H., Nohara T. (2014). Calysolins X-XIII, Resin Glycosides from *Calystegia soldanella*, and Their Antiviral Activity toward Herpes Simplex Virus. Chem. Pharm. Bull..

[32] Xu J.Y., He Y., Zhang A.W., Lu Y., Chen G.T., Yang M., Fan B.Y. (2021). Isolation of Evolvulic Acids B and C, Two New Components of Crude Resin Glycoside Fraction from *Evolvulus alsinoides*. Nat. Prod. Res..

[33] Ono M., Taketomi S., Kakiki Y., Yasuda S., Okawa M., Kinjo J., Miyashita H., Yoshimitsu H., Nohara T. (2021). A New Glycosidic Acid, Muricatic Acid D, and Resin Glycosides, Muricatins X and XI, from the Crude Resin Glycoside Fraction of the Seeds of *Ipomoea muricata*. Chem. Pharm. Bull..

[34] Ono M., Taketomi S., Kakiki Y., Yasuda S., Okawa M., Kinjo J., Yoshimitsu H., Nohara T. (2016). A New Resin Glycoside, Muricatin IX, from the Seeds of *Ipomoea muricata*. Chem. Pharm. Bull..

[35] Reynolds W.F., Yu M., Enriquez R.G., Gonzalez H., Leon I., Magos G. (1995). Isolation and Characterization of Cytotoxic and Antibacterial Tetrasaccharide Glycosides from *Ipomoea stans*. J. Nat. Prod..

[36] Wang L., Yan Y.S., Cui H.H., Yin Y.Q., Pan J.T., Yu B.W. (2017). Three New Resin Glycosides Compounds from *Argyreia acuta* and Their α-Glucosidase Inhibitory Activity. Nat. Prod. Res..

[37] Yu B.W., Sun J.J., Pan J.T., Wu X.H., Yin Y.Q., Yan Y.S., Hu J.Y., McPhee D.J. (2017). Four Pentasaccharide Resin Glycosides from *Argyreia acuta*. Molecules.

[38] Cruz-Morales S., Castañeda-Gómez J., Figueroa-González G., Mendoza-García A.D., Lorence A., Pereda-Miranda R. (2012). Mammalian Multidrug Resistance Lipopentasaccharide Inhibitors from *Ipomoea alba* Seeds. J. Nat. Prod..

[39] Fan B.Y., Li Z.R., Ma T., Gu Y.C., Zhao H.J., Luo J.G., Kong L.Y. (2015). Further Screening of the Resin Glycosides in the Edible Water Spinach and Characterisation on Their Mechanism of Anticancer Potential. J. Funct. Foods.

[40] Lu Y., He Y., Yang M., Fan B.Y. (2021). Arvensic Acids K and L, Components of Resin Glycoside Fraction from *Convolvulus arvensis*. Nat. Prod. Res..

[41] Rosas-Ramírez D., Pereda-Miranda R. (2013). Resin Glycosides from the Yellow-Skinned Variety of Sweet Potato (*Ipomoea batatas*). J. Agric. Food Chem..

[42] Yu B., Luo J., Wang J., Zhang D., Yu S., Kong L. (2013). Pentasaccharide Resin Glycosides from *Ipomoea cairica* and Their Cytotoxic Activities. Phytochemistry.

[43] Pan J.T., Yu B.W., Yin Y.Q., Li J.H., Wang L., Guo L.B., Shen Z. (2015). Four New Pentasaccharide Resin Glycosides from *Ipomoea cairica* with Strong α-Glucosidase Inhibitory Activity. Molecules.

[44] Ono M., Ichihara Y., Saito N., Yamada M., Yuuki K., Nawata M., Tsutsumi S., Yasuda S., Tsuchihashi R., Okawa M. (2020). Identification and Characterization of Organic and Glycosidic Acids in Crude Resin Glycoside Fraction from *Calystegia hederacea*. J. Nat. Med..

[45] Takigawa A., Setoguchi H., Okawa M., Kinjo J., Miyashita H., Yokomizo K., Yoshimitsu H., Nohara T., Ono M. (2011). Identification and Characterization of Component Organic and Glycosidic Acids of Crude Resin Glycoside Fraction from *Calystegia soldanella*. Chem. Pharm. Bull..

[46] Ono M., Takigawa A., Kanemaru Y., Kawakami G., Kabata K., Okawa M., Kinjo J., Yokomizo K., Yoshimitsu H., Nohara T. (2014). Calysolins V-IX, Resin Glycosides from *Calystegia soldanella* and Their Antiviral Activity toward Herpes. Chem. Pharm. Bull..

[47] Fan B.Y., Lu Y., Yang M., Li J.L., Chen G.T. (2019). Evolvulins I and II, Resin Glycosides with a Trihydroxy Aglycone Unit from *Evolvulus alsinoides*. Org. Lett..

[48] Fan B.Y., Lu Y., Xu J.Y., Zhang A.W., Yang M., Chen G.T. (2020). Evolvulin III, a New Resin Glycoside Isolated from *Evolvulus alsinoides*. Phytochem. Lett..

[49] Sura M.B., Ponnapalli M.G., Annam S.C.V.A.R., Bobbili V.V.P. (2019). Ipomeolides A and B, Resin Glycosides from *Ipomoea pes-caprae* and Combination Therapy of Ipomeolide A with Doxorubicin. J. Nat. Prod..

[50] Wang W.Q., Song W.B., Lan X.J., Huang M., Xuan L.J. (2014). Merremins A-G, Resin Glycosides from *Merremia hederacea* with Multidrug Resistance Reversal Activity. J. Nat. Prod..

[51] Ono M., Azuchi M., Ichio M., Jiyoubi Y., Tsutsumi S., Yasuda S., Tsuchihasi R., Okawa M., Kinjo J., Yoshimitsu H. (2019). Seven New Resin Glycosides from the Seeds of *Quamoclit × multifida*. J. Nat. Med..

[52] Ono M., Teramoto S., Naito S., Takahashi A., Yoneda A., Shinkai M., Taga N., Yasuda S., Tsuchihasi R., Okawa M. (2018). Four New Resin Glycosides, Murasakimasarins I–IV, from the Tuber of *Ipomoea batatas*. J. Nat. Med..

[53] Yu B.W., Luo J.G., Wang J.S., Zhang D.M., Yu S.S., Kong L.Y. (2011). Pentasaccharide Resin Glycosides from *Ipomoea pes-caprae*. J. Nat. Prod..

[54] Ono M., Takigawa A., Mineno T., Yoshimitsu H., Nohara T., Ikeda T., Fukuda-Teramachi E., Noda N., Miyahara K. (2010). Acylated Glycosides of Hydroxy Fatty Acid Methyl Esters Generated from the Crude Resin Glycoside (Pharbitin) of Seeds of *Pharbitis nil* by Treatment with Indium(III) Chloride in Methanol. J. Nat. Prod..

[55] Ono M., Takaki Y., Takatsuji M., Akiyama K., Okawa M., Kinjo J., Miyashita H., Yoshimitsu H., Nohara T. (2012). Three New Resin Glycosides and a New Tetrahydropyran Derivative from the Seeds of *Quamoclit pennata*. Chem. Pharm. Bull..

[56] Ding W., Jiang Z.H., Wu P., Xu L., Wei X. (2012). Resin Glycosides from the Aerial Parts of *Operculina turpethum*. Phytochemistry.

[57] Koryudzu K., Arai M.A., Ahmed F., Sadhu S.K., Ishibashi M. (2012). A New Resin Glycoside from *Ipomoea maxima*. Nat. Prod. Commun..

[58] Ono M., Saito N., Minamishima H., Yasuda S., Tsuchihashi R., Okawa M., Kinjo J., Miyashita H., Yoshimitsu H., Nohara T. (2021). Two New Glycosidic Acids, Calyhedic Acids E and F, in Crude Resin Glycoside Fraction from *Calystegia hederacea*. Nat. Prod. Res..

[59] Ono M., Yuhara N., Shimohara T., Matsubara S., Yasuda S., Tsuchihashi R., Okawa M., Kinjo J., Zhou J.R., Yoshimitsu H. (2021). Calyhedins I–VI: Resin Glycosides from the Rhizomes of *Calystegia hederacea*. Phytochemistry.

[60] Ono M., Shimohara T., Yuhara N., Matsubara S., Yasuda S., Tsuchihashi R., Okawa M., Kinjo J., Yoshimitsu H., Nohara T. (2021). Four New Resin Glycosides, Calyhedins VII–X, from the Rhizomes of *Calystegia hederacea*. Nat. Prod. Res..

[61] Lv K.Q., Ji H.Y., Du G.X., Peng S., Guo P.J., Wang G., Zhu Y., Wang Q., Wang W.Q., Xuan L.J. (2022). Calysepins I-VII, Hexasaccharide Resin Glycosides from *Calystegia sepium* and Their Cytotoxic Evaluation. J. Nat. Prod..

[62] Ono M., Takigawa A., Muto H., Kabata K., Okawa M., Kinjo J., Yokomizo K., Yoshimitsu H., Nohara T. (2015). Antiviral Activity of Four New Resin Glycosides Calysolins XIV-XVII from *Calystegia soldanella* against Herpes Simplex Virus. Chem. Pharm. Bull..

[63] Akiyama K., Mineno T., Okawa M., Kinjo J., Miyashita H., Yoshimitsu H., Nohara T., Ono M. (2013). Three Acylated Glycosidic Acid Methyl Esters and Two Acylated Methyl Glycosides Generated from the Convolvulin Fraction of Seeds of *Quamoclit pennata* by Treatment with Indium(III) Chloride in Methanol. Chem. Pharm. Bull..

[64] Fan B.Y., Lu Y., Yin H., He Y., Li J.L., Chen G.T. (2018). Arvensic Acids A-D, Novel Heptasaccharide Glycosidic Acids as the Alkaline Hydrolysis Products of Crude Resin Glycosides from *Convolvulus arvensis*. Fitoterapia.

[65] Akiyama K., Yamamoto K., Mineno T., Okawa M., Kinjo J., Yoshimitsu H., Nohara T., Ono M. (2014). Five New Resin Glycoside Derivatives Isolated from the Convolvulin Fraction of Seeds of *Quamoclit pennata* after Treatment with Indium(III) Chloride in Methanol. Chem. Pharm. Bull..

[66] Bautista E., Fragoso-Serrano M., Pereda-Miranda R. (2015). Jalapinoside, a Macrocyclic Bisdesmoside from the Resin Glycosides of *Ipomea purga*, as a Modulator of Multidrug Resistance in Human Cancer Cells. J. Nat. Prod..

[67] Bautista E., Fragoso-Serrano M., Pereda-Miranda R. (2016). Jalapinoside II, a Bisdesmoside Resin Glycoside, and Related Glycosidic Acids from the Officinal Jalap Root (*Ipomoea purga*). Phytochem. Lett..

[68] Ono M., Akiyama K., Kishida M., Okawa M., Kinjo J., Yoshimitsu H., Miyahara K. (2013). Two New Glycosidic Acids, Multifidinic Acids F and G, of the Ether-Insoluble Resin Glycoside (Convolvulin) from the Seeds of *Quamoclit × multifida*. J. Nat. Med..

[69] Ono M., Akiyama K., Yamamoto K., Mineno T., Okawa M., Kinjo J., Miyashita H., Yoshimitsu H., Nohara T. (2014). Four New Acylated Glycosidic Acid Methyl Esters Isolated from the Convolvulin Fraction of Seeds of *Quamoclit pennata* after Treatment with Indium(III) Chloride in Methanol. Chem. Pharm. Bull..

[70] Ono M., Imao M., Miyahara K. (2010). Two New Glycosidic Acids, Quamoclinic Acids G and H, of the ResinGlycosides (Convolvulin) from the Seeds of Quamoclit pennata. Chem. Pharm. Bull. (Tokyo).

[71] Rosas-Ramírez D., Escalante-Sánchez E., Pereda-Miranda R. (2011). Batatins III-VI, Glycolipid Ester-Type Dimers from *Ipomoea batatas*. Phytochemistry.

[72] Bah M., Pereda-Miranda R. (1996). Detailed FAB-Mass Spectrometry and High Resolution NMR Investigations of Tricolorins A-E, Individual Oligosaccharides from the Resins of *Ipomoea tricolor* (Convolvulaceae) 1. Tetrahedron.

[73] Noda N., Tsuji K., Kawasaki T., Miyahara K., Hanazono H., Yang C.-R. (1995). A Novel Resin Glycoside, Merremin (Tuguajalapin X Dimer), from *Merremia hungaiensis*. Chem. Pharm. Bull..

[74] Ono M., Kawasaki T., Miyahara K. (1989). Resin Glycosides. V. Identification and Characterization of the Component Organic and Glycosidic Acids of the Ether-Soluble Crude Resin Glycosides (“Jalapin”) from Rhizoma Jalapae Braziliensis (Roots of *Ipomoea operculata*). Chem. Pharm. Bull..

[75] Chérigo L., Pereda-Miranda R. (2006). Resin Glycosides from the Flowers of *Ipomoea murucoides*. J. Nat. Prod..

[76] Maria Gaspar E.M. (2001). Soldanelline B The First Acylated Nonlinear Tetrasaccharide Macrolactone from the European Convolvulaceae *Calystegia soldanella*. Eur. J. Org. Chem..

[77] Zhu D., Chen C., Xia Y., Kong L.Y., Luo J. (2019). A Purified Resin Glycoside Fraction from Pharbitidis Semen Induces Paraptosis by Activating Chloride Intracellular Channel-1 in Human Colon Cancer Cells. Integr. Cancer Ther..

[78] Li J.H., Pan J.T., Yin Y.Q. (2016). Two Novel Resin Glycosides Isolated from *Ipomoea cairica* with α-Glucosidase Inhibitory Activity. Chin. J. Nat. Med..

[79] León-Rivera I., Herrera-Ruiz M., Estrada-Soto S., Gutiérrez M.D.C., Martínez-Duncker I., Navarrete-Vázquez G., Rios M.Y., Aguilar B., Castillo-España P., Aguirre-Moreno A. (2011). Sedative, Vasorelaxant, and Cytotoxic Effects of Convolvulin from *Ipomoea tyrianthina*. J. Ethnopharmacol..

[80] León-Rivera I., Mirón-López G., Molina-Salinas G.M., Herrera-Ruiz M., Estrada-Soto S., Gutiérrez M.D.C., Alonso-Cortes D., Navarrete-Vázquez G., Ríos M.Y., Said-Fernández S. (2008). Tyrianthinic Acids from Ipomoea Tyrianthina and Their Antimycobacterial Activity, Cytotoxicity, and Effects on the Central Nervous System. J. Nat. Prod..

[81] Ono M., Fukuda H., Murata H., Miyahara K. (2009). Resin Glycosides from the Leaves and Stems of Ipomoea Digitata. J. Nat. Med..

[82] Çaliş I., Sezgin Y., Dönmez A.A., Rüedi P., Tasdemir D. (2007). Crypthophilic Acids A, B, and C: Resin Glycosides from Aerial Parts of *Scrophularia crypthophila*. J. Nat. Prod..

[83] Yin Y.-Q., Huang X.-F., Kong L.-Y., Niwa M. (2008). Three New Pentasaccharide Resin Glycosides from the Roots of Sweet Potato (*Ipomoea batatas*). Chem. Pharm. Bull..

[84] Tao H., Hao X., Liu J., Ding J., Fang Y., Gu Q., Zhu W. (2008). Resin Glycoside Constituents of *Ipomoea pes-caprae* (Beach Morning Glory). J. Nat. Prod..

[85] Escalante-Sánchez E., Rosas-Ramírez D., Linares E., Bye R., Pereda-Miranda R. (2008). Batatinosides II-VI, Acylated Lipooligosaccharides from the Resin Glycosides of Sweet Potato. J. Agric. Food Chem..

[86] Escobedo-Martínez C., Pereda-Miranda R. (2007). Resin Glycosides from *Ipomoea pes-caprae*. J. Nat. Prod..

[87] Bah M., Chérigo L., Cardoso Taketa A.T., Fragoso-Serrano M., Hammond G.B., Pereda-Miranda R. (2007). Intrapilosins I-VII, Pentasaccharides from the Seeds of *Ipomoea intrapilosa*. J. Nat. Prod..

[88] Noda N., Horiuchi Y. (2008). The Resin Glycosides from the Sweet Potato (*Ipomoea batatas* L. LAM.). Chem. Pharm. Bull..

[89] Zhu S.Y., Huang J.S., Zheng S.S., Zhu K., Yang J.S. (2013). First Total Synthesis of the Proposed Structure of Batatin VI. Org. Lett..

[90] Nawój M., Grobelny A., Mlynarski J. (2020). Macrolide Core Synthesis of Calysolin IX Using an Intramolecular Glycosylation Approach. Eur. J. Org. Chem..

[91] Huang H., Yang C.S., Zeng L.N., Zhang L.L. (2015). Synthesis of Key Macrolactone Structure of Resin Glycosides Using a Keck Macrolactonization Method. J. Asian Nat. Prod. Res..

[92] Xie L., Zhu S.Y., Shen X.Q., He L.L., Yang J.S. (2010). Total Synthesis of Batatoside L. J. Org. Chem..

[93] Sun J., Fang J., Xiao X., Cai L., Zhao X., Zeng J., Wan Q. (2020). Total Synthesis of Tricolorin A via interrupted Pummerer Reaction-Mediated Glycosylation and One-Pot Relay Glycosylation. Org. Biomol. Chem..

[94] Fang J., Zeng J., Sun J., Zhang S., Xiao X., Lu Z., Meng L., Wan Q. (2019). Total Syntheses of Resin Glycosides Murucoidins IV and V. Org. Lett..

[95] Son S.H., Yanagiya N., Furukawa J.I., Sakairi N. (2009). Intramolecular Glycosylation Approach toward Constructing the Macrocyclic Structure of Resin Glycosides. Synlett.

[96] Nagano T., Pospíšil J., Chollet G., Schulthoff S., Hickmann V., Moulin E., Herrmann J., Müller R., Fürstner A. (2009). Total Synthesis and Biological Evaluation of the Cytotoxic Resin Glycosides Ipomoeassin A-F and Analogues. Chem. - A Eur. J..

[97] Noda N., Kobayashi H., Miyahara K., Kawasaki T. (1988). Resin Glycosides. III. Isolation and Structural Study of the Genuine Resin Glycosides, Muricatins I-VI, from the Seeds of Ipomoea Muricata. Chem. Pharm. Bull..

